# Highly Activated Terminal Carbon Monoxide Ligand in an Iron–Sulfur Cluster Model of FeMco with Intermediate Local Spin State at Fe

**DOI:** 10.1021/jacs.3c12025

**Published:** 2024-02-15

**Authors:** Linh N. V. Le, Justin P. Joyce, Paul H. Oyala, Serena DeBeer, Theodor Agapie

**Affiliations:** † Division of Chemistry and Chemical Engineering, California Institute of Technology, Pasadena, California 91125, United States; ‡ Max Planck Institute for Chemical Energy Conversion, Stiftstraße 34-36, 45470 Mülheim an der Ruhr, Germany

## Abstract

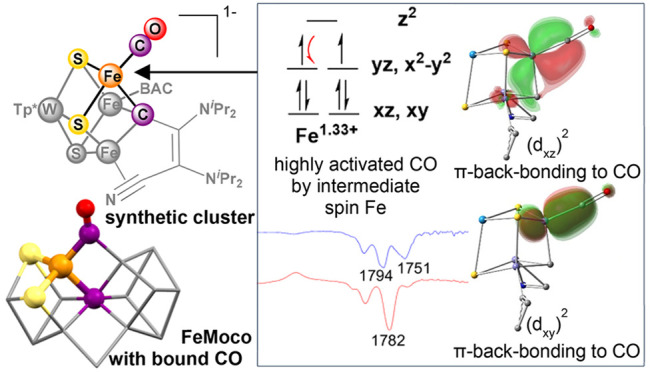

Nitrogenases, the enzymes that convert N_2_ to NH_3_, also catalyze the reductive coupling of CO to yield hydrocarbons. CO-coordinated species of nitrogenase clusters have been isolated and used to infer mechanistic information. However, synthetic FeS clusters displaying CO ligands remain rare, which limits benchmarking. Starting from a synthetic cluster that models a cubane portion of the FeMo cofactor (FeMoco), including a bridging carbyne ligand, we report a heterometallic tungsten–iron–sulfur cluster with a single terminal CO coordination in two oxidation states with a high level of CO activation (ν_CO_ = 1851 and 1751 cm^–1^). The local Fe coordination environment (2S, 1C, 1CO) is identical to that in the protein making this system a suitable benchmark. Computational studies find an unusual intermediate spin electronic configuration at the Fe sites promoted by the presence the carbyne ligand. This electronic feature is partly responsible for the high degree of CO activation in the reduced cluster.

Substrate activation at complex inorganic cofactors in enzyme active sites has raised fundamental questions about the role of the cluster structure on reactivity. For example, the challenging conversion of N_2_ to NH_3_ by nitrogenase enzymes occurs at FeMo cofactor (FeMoco) (M = Mo, V, or Fe), which comprises complex double cubane clusters with the MFe_7_S_9_C composition.^1,2^ Nitrogenases also catalyze the reductive coupling of CO to form hydrocarbons for M = Mo and V.^3,4^ Despite interest in these transformations, the characterization of substrate-bound clusters is very rare, which limits insight into the site of small molecule activation and reaction mechanism.^5−11^ Only two CO-bound species of FeMoco and FeVco have been characterized structurally.^9,10,12,13^ Structural characterization of N_2_-derived species remains debated.^14−16^

Synthetic models promise to facilitate a better understanding of the impact of cluster structure on substrate binding and level of activation.^17−22^ However, few examples of synthetic iron–sulfur clusters with terminal or bridging N_2_ or CO ligands have been reported, many of which possess multiple CO ligands that drastically alter the electronic structure of the cluster and complicate comparisons to FeMoco (Figure 1).^23−29^ Only one type of FeS cluster with a single terminal CO ligand has been characterized, ligated by three carbene ligands.^30,31^

**Figure 1:**
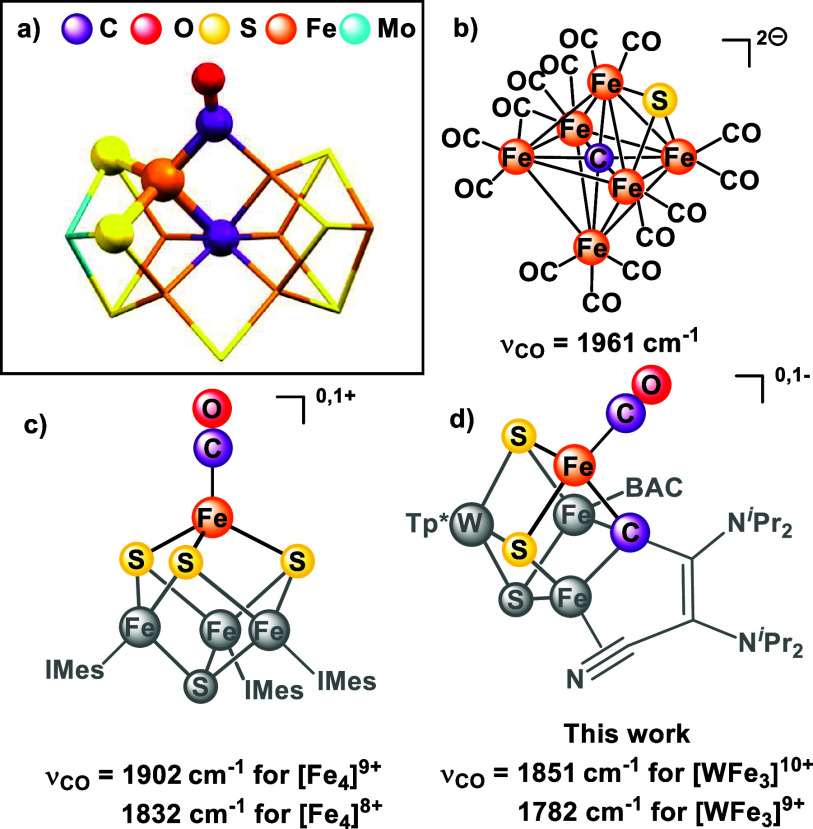
Structures of FeS clusters with CO coordination: (a) CO-bound FeMoco (PDB: 4TKV); (b) synthetic cluster with carbide ligand;^26,27^ (c) Fe_4_S_4_ cluster with a single terminal CO;^30^ (d) present report. Local coordination sphere of Fe–CO moiety highlighted in (a), (c), and (d).

Having accessed a partial synthetic analogue **1** of the cluster core of FeMoco displaying a μ_3_-carbyne ligand with the WFe_3_S_3_CR composition, where W is the isoelectronic analogue of Mo,^32^ we targeted the coordination of nitrogenase substrates (Scheme 1).^33^ Herein, we report the reactivity of **1** with isocyanides and CO, which affords an FeS cubane with a single terminal CO. We characterize this cluster in two oxidation states, which show a high level of CO activation, as observed in the low CO stretching frequency (1751–1851 cm^–1^) by IR spectroscopy.

**Scheme 1:**
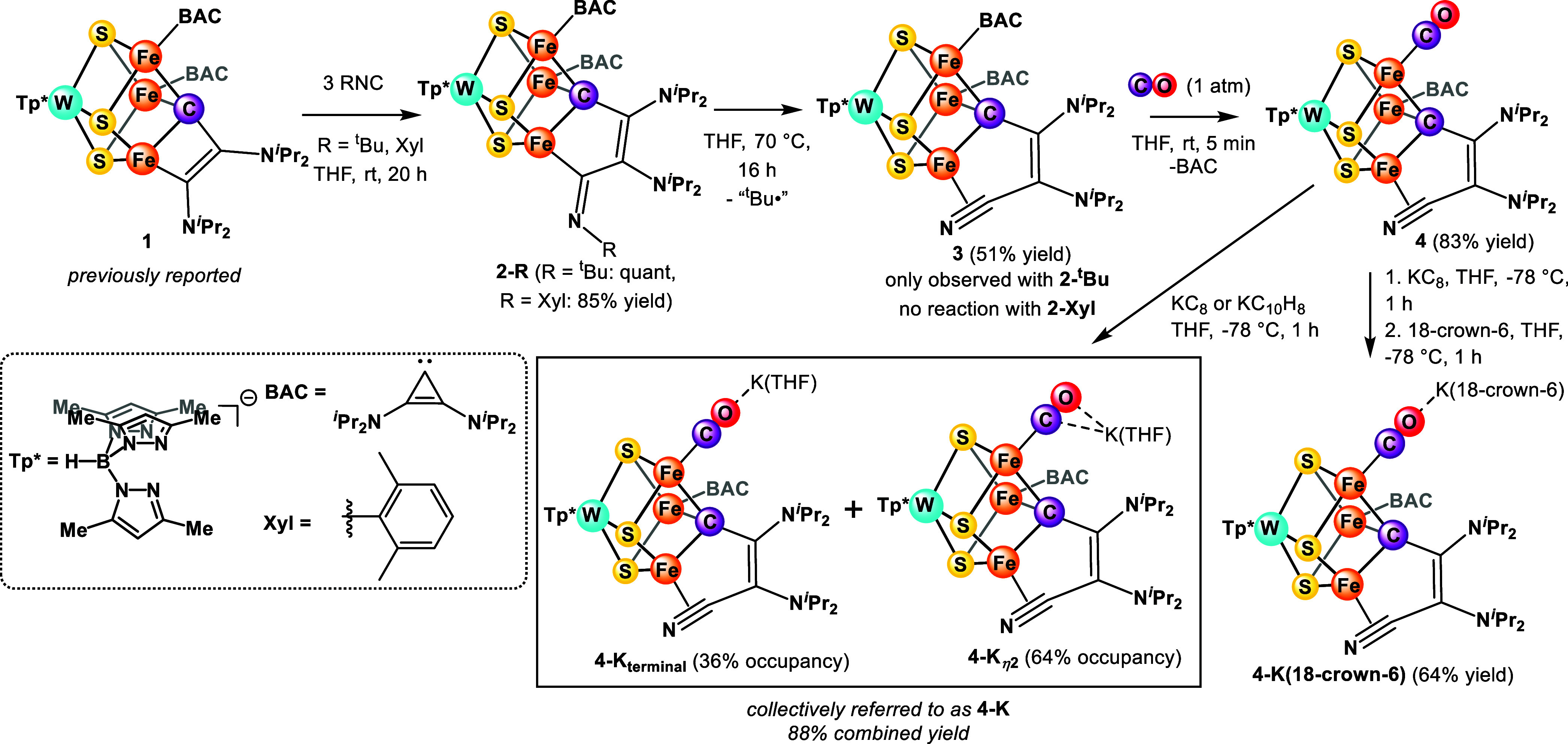
Syntheses of Clusters

We employed isocyanides as isoelectronic analogues of CO and substrates of nitrogenase^34^ that also allow for a more controlled reactivity. Treating **1** with ^*t*^BuNC or XylNC (Xyl = 2,6-dimethylphenyl) gives **2-**^***t***^**Bu** or **2-Xyl** (Scheme 1), respectively, through the insertion of isocyanide into the Fe–C(vinyl) bond, which demonstrates rare examples of C–C bond formation at an FeS cluster.^35−38^ Heating **2-**^***t***^**Bu** in THF at 70 °C for 16 h leads to the formation of **3**, where XRD and NMR studies are consistent with the loss of a ^*t*^Bu radical (leaving an η^2^-nitrile ligand).^39^ While determining the protonation state of the N atom solely on the basis of XRD is inconclusive, the short C–N bond length of 1.205(6) Å compared with ∼1.25 Å for η^2^-iminoacyl (see the Supporting Information for additional literature comparison and support by ATR IR spectroscopy) is indicative of an η^2^-nitrile motif.^40^ Loss of the ^*t*^Bu radical suggests a propensity for side-on nitrile binding, which is an intriguing observation in the context of the nitrogenase substrates displaying triple bonds, including N_2_, acetylene, and isocyanides.^41^ The conversion from **2-**^***t***^**Bu** to **3**, which involves the loss of a ^*t*^Bu radical, formally represents one-electron oxidation of the WFe_3_ metal core. In contrast to **2-**^***t***^**Bu**, **2-Xyl** is stable under the same conditions, which is consistent with a lower tendency to lose the more reactive aryl radical.^42^

With **3** in hand, we explored reactions with CO. Cluster **3** reacts with 1 atm CO to form **4** within 5 min, which shows substitution of one bis(diisopropylamino)cyclopropenylidene (BAC) ligand with CO (83% yield, Scheme 1) in an uncommon instance of carbene lability.^43^ The average Fe–C(μ_3_) distance remains similar to **2-**^***t***^**Bu** and **3** at 1.95 Å, but the range for the individual bond lengths increases to 1.88–2.00 Å (compared with 1.92–1.95 Å in **2-**^***t***^**Bu** and 1.95–1.96 Å in **3**), which suggests that the carbyne ligand, and potentially the carbide in FeMoco, has the ability to accommodate distinct electronic demands of different Fe centers through structural changes.^44^ This is in contrast to spectroscopic studies suggesting that the central carbide serves to maintain the rigid core structure.^8,45^

To the best of our knowledge, **4** is the only well-characterized example of a heterometallic MFe_3_S_3_(CR) cubane cluster bearing a single terminal CO ligand. This provides an opportunity for benchmarking the impact of structure and coordination environment relative to FeMoco. The THF solution IR spectrum of **4** displays a prominent peak at 1851 cm^–1^, assigned as the C–O stretch (Figure 3) and confirmed by ^13^CO labeling (ν_13CO exp_ = 1807 cm^–1^, ν_13CO calc_ = 1810 cm^–1^), thereby suggesting highly activated CO.

To study the effects of cluster oxidation state on the level of CO activation, we reduced **4** with one equivalent of KC_8_ or potassium naphthalenide to yield **4-K** (S = 3/2, see the Supporting Information) (Scheme 1). As expected, the CO bond length increases upon reduction from 1.15(1) to 1.198(3) Å. The solution IR spectrum of **4-K** shows two C–O bands at 1794 and 1751 cm^–1^ (Figure 3), which is consistent with the crystal structure of **4-K** displaying CO–K^+^ interactions disordered over two positions: terminal (36% occupancy) (assigned as **4-K**_**terminal**_) and η^2^ (64% occupancy) (assigned as **4-K**_**η2**_). These isomers are collectively referred to as **4-K**. Chelation of K^+^ with 18-crown-6 results in the formation of **4-K(18-crown-6)**. XRD shows that the K^+^ ion is present in only one location and interacts end-on with the O atom of CO (Figure 2). In agreement, the IR spectrum shows a single band at 1782 cm^–1^ (Figure 3; ν_13CO exp_ = 1740 cm^–1^_;_ ν_13CO calc_ = 1742 cm^–1^). The same band is observed upon treatment with [2.2.2]cryptand, thereby suggesting that the K^+^ ion in **4-K(18-crown-6)** does not impact CO activation substantially.^46^

**Figure 2:**
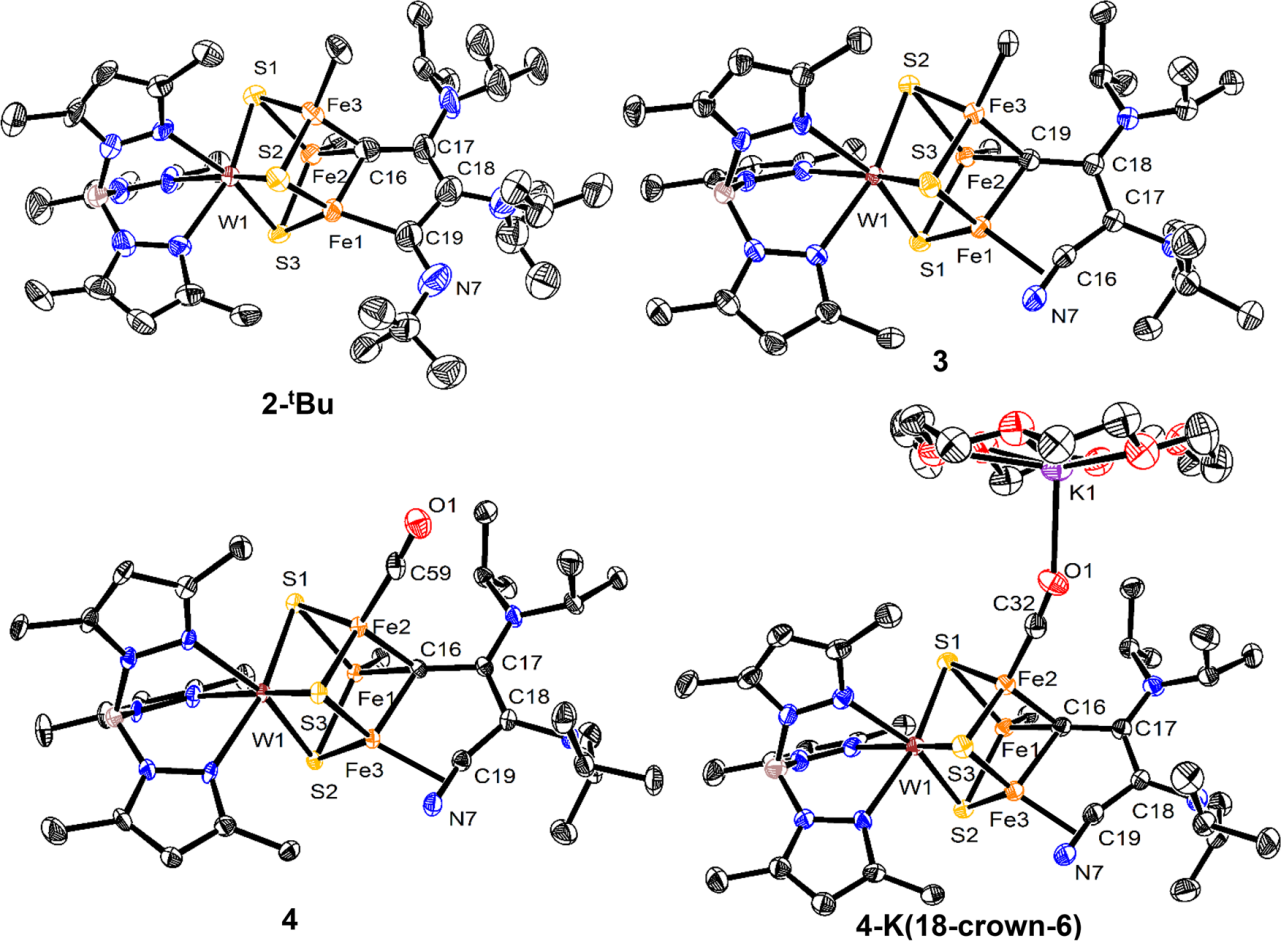
Crystal structures of **2-**^***t***^**Bu**, **3**, **4**, and **4-K(18-crown-6)**. Ellipsoids are shown at 50% probability level. Hydrogen atoms, solvent molecules, and the BAC ligand, except for the carbene C, are omitted for clarity.

**Figure 3:**
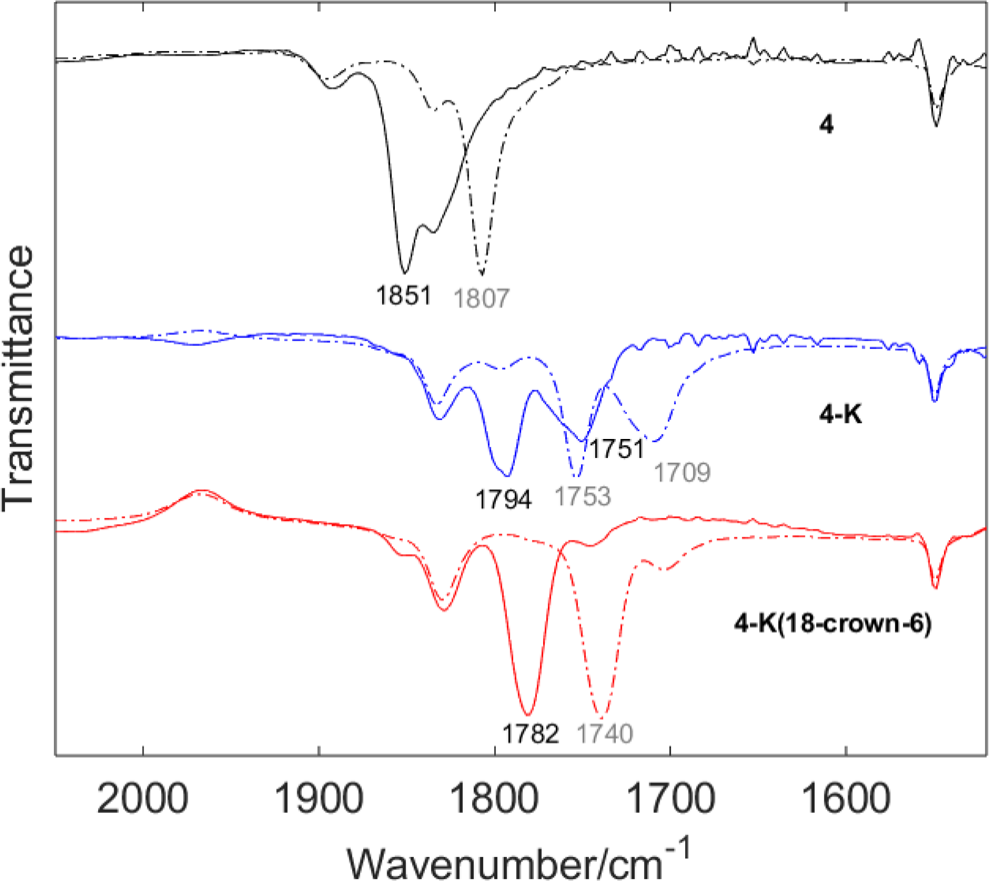
IR spectra of **4**, **4-K**, and **4-K(18-crown-6)** (THF solution) with ν_CO_ values shown. Dashed spectra correspond to ^13^CO-labeled species with ν_13CO_ in gray. The feature at 1830 cm^–1^ unchanged upon ^13^CO labeling is assigned to BAC.

Both **4-K** and **4-K(18-crown-6)** exhibit highly activated CO ligands coordinated to Fe in a terminal fashion. The interaction with K^+^ in different binding modes affects the level of CO activation in the 1794 and 1751 cm^–1^ range. Previous computational work describes a semibridging CO ligand at Fe2 in FeMoco with a frequency of 1718 cm^–1^,^47^ very close to that assigned to the bridging CO in lo-CO at 1715 cm^–1^.^48^ This is slightly lower than the typical values observed for μ_2_-CO ligands, which lie in the 1720–1850 cm^–1^ range.^49^ Hydrogen bonding between the carbonyl oxygen and the nearby His195 residue is proposed to further activate CO.^47^ Similarly, in **4-K**, the K^+^ cation can play the same role as the hydrogen bonding network and lower the C–O stretching frequency. Nevertheless, ν_CO_ values below 1800 cm^–1^ are unprecedented for FeS clusters. For comparison, the CO adducts of *N*-heterocyclic carbene (NHC)-supported Fe_4_S_4_ clusters reported by Suess and co-workers display C–O stretching frequencies of 1832 cm^–1^ for the [Fe_4_S_4_]^0^ and 1902 cm^–1^ for the [Fe_4_S_4_]^+^ states.^30^ The local coordination environment at each Fe (FeS_2_C in **4** and **4-K** and FeS_3_ in [Fe_4_S_4_]^+,0^) and oxidation state distribution between different metal sites can contribute to the level of diatomic activation.^30,50,51^

In order to understand the electronic structure origin of the profound CO activation in these clusters, we employed computational methods using broken symmetry density functional theory (BS-DFT). Our computational procedure detailed in the Supporting Information accurately assigns the geometric, Mössbauer, and vibrational properties of **4** and **4-K**. Here, we highlight the impact of the carbyne, W^3+^ center, and a K^+^ countercation with respect to the strong CO activation in **4-K**.

The carbyne has three anionic lone pairs oriented along the Fe-bonding axes in its μ_3_-binding mode. The localized orbitals characterize the carbyne lone pairs as σ-donors that stabilize the intermediate spin (IS) state of the three formal Fe^2+^ (*S* = 1) centers. Observing the IS state at the Fe sites that do not bind CO suggests that it is an innate property of the μ_3_-carbyne ligand. The IS state in Fe^2+^ centers give full occupation of its π-backbonding orbitals, consistent with the increased CO activation in **4-K**. In agreement, hyperfine sublevel correlation (HYSCORE) spectra of **4-K(**^**13**^**CO)** show small hyperfine coupling to the ^13^C center of CO {*A*(^13^C) = [−0.5, 1.0, −0.5] MHz; see the Supporting Information}. A partially occupied Fe–CO backbonding orbital is expected to result in larger coupling.^5,52,53^ In comparison, Fe centers in FeS clusters are routinely assigned as high-spin because of their weak ligand field environment, such as the *S* = 3/2 state assigned to the CO-bound Fe^1+^ by Suess and co-workers.^30^

Furthermore, the Fe centers are preferentially ferromagnetically coupled, which results in the equal delocalization of two electrons among the three Fe atoms (Figure 4). This formally lowers the oxidation state of the CO-bound Fe site from its formal 2+ to 1.33+ charge and proportionately increases the other Fe centers to 2.33+; their resonance states are illustrated in the Supporting Information. This is analogous to the net Fe^2.5+^ oxidation state resulting from the equal delocalization of one electron between two Fe sites in formal Fe^2+^–Fe^3+^ dimers.^54^ This pairwise delocalization supports a reduced state at the CO-bound center that is otherwise inaccessible under biological conditions. Similarly, redox disproportionation has been proposed in previously reported [Fe_6_(μ_6_-C)(CO)_18_] and Fe_4_S_4_(CO)(IMes)_3_ clusters, where Fe sites of different oxidation states are within close proximity.^30,55^

**Figure 4:**
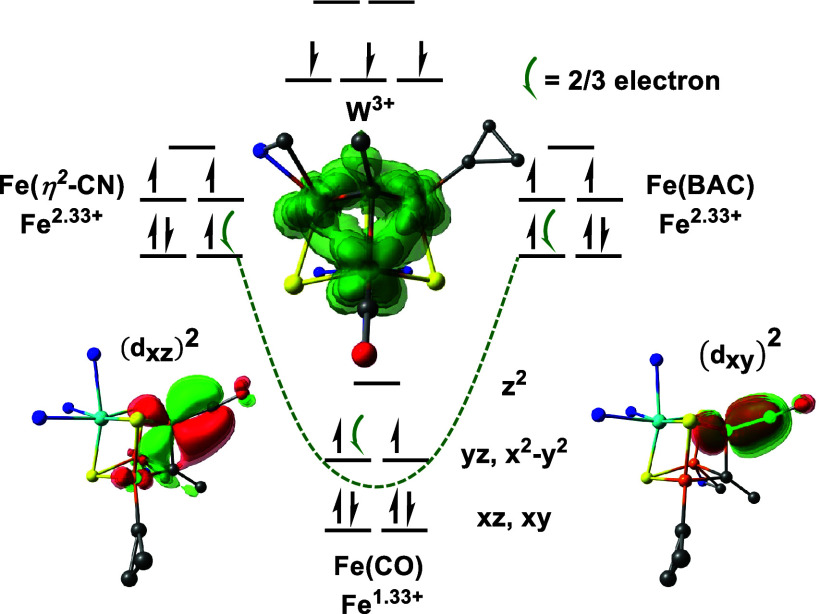
Local oxidation and spin states of the metal centers of **4**^–^ (*S* = 3/2) with respect to the Mulliken spin population of their PM-localized orbitals (Figures S34–36).The curved green arrow denotes a pair of electrons that are equally delocalized among the Fe centers (illustrated in the inset) with respect to its localized spin density. The degenerate Fe–CO π-bonding interactions are shown at the bottom with respect to their localized orbitals.

The anionic charge of **4**^**–**^ supports strong noncovalent interactions with its countercation. The geometry optimization of **4-K** preferentially binds K^+^ in an η^2^-conformation with respect to the CO bond. The calculated CO stretching frequency decreases from 1800 cm^–1^ without K^+^ to 1756 cm^–1^, which is consistent with the distinct vibrational modes observed in the IR spectrum of **4-K**. The electronic structure of the cluster is not impacted by K coordination, thereby suggesting that it is a purely ionic interaction that stabilizes the π-bonding of the CO ligand.

The CO lone pair can overlap with orbitals arising from the Fe–W interaction assigned as purely covalent in **4**^**–**^ on the basis of the localized orbitals (see Figure S34 for a graphical representation). The Fe–W covalent interaction redistributes electron density between the metal centers promoting the electrostatic attraction with the CO lone pair and consequently also enhances the π*-backbonding discussed above.^56,57^ The other Fe centers exhibit bonding characters that are intermediate of a covalent and magnetic interaction, analogous to bonding properties detailed in the Mo^3+^ heteroatom of FeMoco.^58,59^ In contrast, this is not observed for the cluster reported by Suess and co-workers^30^ because of the comparatively weak bonding interactions between Fe sites. Overall, these factors contribute to the stronger CO activation in **4**^**–**^ compared with these reported clusters with an average metal oxidation state of 2+, despite the higher average metal oxidation state of 2.25+ in **4**^**–**^.^30^

In summary, we have reported a series of heterometallic WFe_3_S_3_CR cubanes and demonstrated several types of organometallic transformations and binding modes that are rare for iron–sulfur clusters. These compounds show C–C coupling, along with side-on binding of an organic nitrile moiety at one Fe site. Furthermore, we characterized the first example of a heterometallic iron–sulfur cluster with a single terminally bound, highly activated CO ligand in two oxidation states. Computation suggests an unusual carbyne-promoted intermediate spin electronic configuration at all Fe sites, along with a low oxidation state of 1.33+ for Fe(CO) in **4**^**–**^. This electron configuration affords full occupancy of the two π-back-bonding orbitals to CO, which are responsible for the high level of CO activation in the reduced clusters. The negative charge of the cluster and the metal–metal covalency were found computationally to also impact CO activation. These findings provide a set of parameters to evaluate in future studies for the conversion of substrates in nitrogenase.

## Supporting Information Available

General methods, synthetic procedures, product isolation and characterization, NMR spectra, structural information, and computational methods (PDF)
