# Supplementary material for: Highly Activated Terminal Carbon Monoxide Ligand in an Iron–Sulfur Cluster Model of FeMco with Intermediate Local Spin State at Fe
Source: J Am Chem Soc. 2024 Feb 15;146(8):5045–50. doi: 10.1021/jacs.3c12025 (PMC10910499; doi:10.1021/jacs.3c12025)
Supplement: Supplementary file 1 — ja3c12025_si_001.pdf [file ja3c12025_si_001.pdf]

Supporting Information for

Highly Activated Terminal Carbon Monoxide Ligand in an Iron-Sulfur Cluster Model of FeMoco

Linh N. V. Le[1], Justin P. Joyce[2], Serena DeBeer[2],* and Theodor Agapie[1]*

[1]Division of Chemistry and Chemical Engineering, California Institute of Technology, Pasadena, California 91125, United States
[2]Max Planck Institute for Chemical Energy Conversion, Stiftstraße 34-36, 45470 Mülheim an der Ruhr, Germany

*To whom correspondence may be addressed. **Email:** agapie@caltech.edu serena.debeer@cec.mpg.de

Table of Contents:

A) Synthetic details and characterization **2**
1. General considerations 2
2. Procedures 2
Synthesis of **2-^{t}Bu** 2
Synthesis of **2-Xyl** 3
Synthesis of **3** 3
Synthesis of **4** 4
Synthesis of **4-K** 5
Synthesis of **4-K(18-crown-6)** 5
Synthesis of **5** 6
Reaction of **1** with 1 atm CO 6
3. NMR spectra 8
4. Support for assignment of 3 **17**
5. Physical methods **19**
Mössbauer spectroscopy 19
Electrochemical measurements 23
Additional IR spectra 26
Evans method for **4** 30
EPR spectroscopy 30
B) Crystallographic information **37**
C) Computational details **47**
D) References **56**

A) Synthetic details and characterization:

1. *General considerations:*

All reactions were performed at room temperature in a N_2-filled MBraun glovebox or using standard Schlenk techniques unless otherwise specified. Glassware was oven-dried at 140 °C for at least 2 h prior to use and allowed to cool under vacuum. **1** was prepared according to literature procedures.[1] Diethyl ether, benzene, tetrahydrofuran (THF), and pentane were dried by sparging with N_2 for at least 15 min and then passing through a column of activated A2 alumina under positive N_2 pressure, and stored over 3 Å molecular sieves prior to use. ^{1}H spectra were recorded on a Varian 300 MHz spectrometer. Deuterated benzene (C_6D_6) was purchased from Cambridge Isotope Laboratories, dried over sodium/benzophenone ketyl, degassed by three freeze–pump–thaw cycles, and vacuum-transferred prior to use. IR spectra were obtained as either solution samples using a KBr window cell on a Thermo Scientific Nicolet 6700 FT-IR spectrometer or thin films formed by evaporation of solutions using a Bruker Alpha Platinum ATR spectrometer with OPUS software in a glovebox under an N_2 atmosphere.

2. *Procedures:*

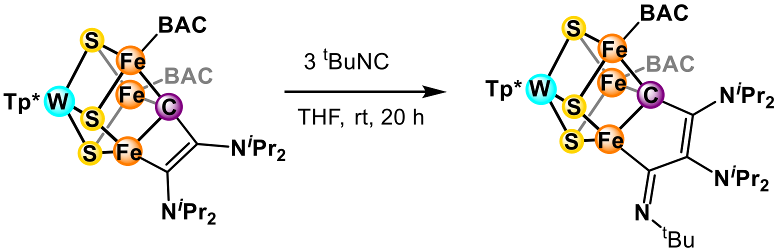

1 **2-^{t}Bu**

Synthesis of **2-^{t}Bu**. In a glovebox, **1** (300.0 mg, 0.206 mmol, 1 equiv) was dissolved in THF (15 mL). To this solution, ^{t}BuNC (70 μL, 0.619 mmol, 3 equiv) was added to the reaction using a microsyringe. The reaction was stirred at room temperature for 20 h, after which the volatiles were removed *in vacuo*. The crude material was used without further purification. Yield: 317 mg (quant). X-ray quality crystals of **2-^{t}Bu** were grown by first washing the crude material with pentane and Et_2O, extracting the product into C_6H_6 and diffusing HMDSO into a concentrated C_6H_6 solution for several days. ^{1}H NMR (400 MHz, THF-h_8, solvent suppression) δ 12.91, 7.28, 6.89, 6.14, 5.56, 5.13, 0.42, -1.57, -2.77, -2.95, -3.35, -3.76, -4.98, -6.36. Anal. calcd (%) $C_{65}H_{115}BFe_3N_{13}S_3W$ (M_r = 1537.09): C, 50.79; H, 7.54; N, 11.85. Found: C, 50.55; H, 8.44; N, 11.54.

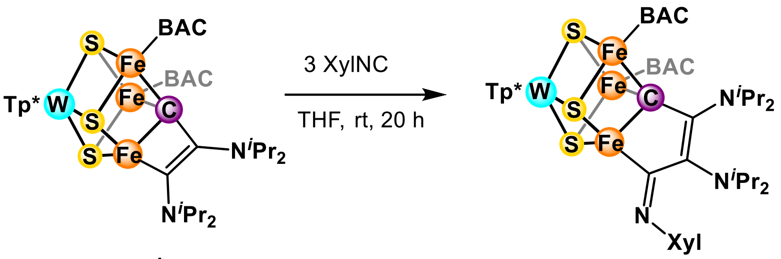

1 **2-Xyl**

Synthesis of **2-Xyl**. In a glovebox, **1** (210.0 mg, 0.144 mmol, 1 equiv) and XylNC (56.8 mg, 0.433 mmol, 3 equiv) were combined in THF (5 mL). The reaction was stirred at room temperature for 20 h, after which the volatiles were removed *in vacuo*. The crude material was triturated three times with Et_2O and washed with Et_2O. The solid was redissolved in a minimal amount of THF, filtered and crystallized by THF/pentane vapor diffusion. Yield: 195 mg (85%). X-ray quality crystals were grown by diffusing Et_2O into a concentrated solution of **2-Xyl** in THF. ^{1}H NMR (400 MHz, THF-h_8, solvent suppression) δ 15.53, 11.59, 11.10, 9.63, 7.26, 6.99, 6.45, 6.01, 5.54, 0.24, -0.66, -1.80, -1.86, -2.77, -3.36. Anal. calcd (%) $C_{69}H_{115}BFe_3N_{13}S_3W$ (M_r = 1585.14): C, 52.28; H, 7.31; N, 11.49. Found: C, 51.24; H, 7.35; N, 12.44.

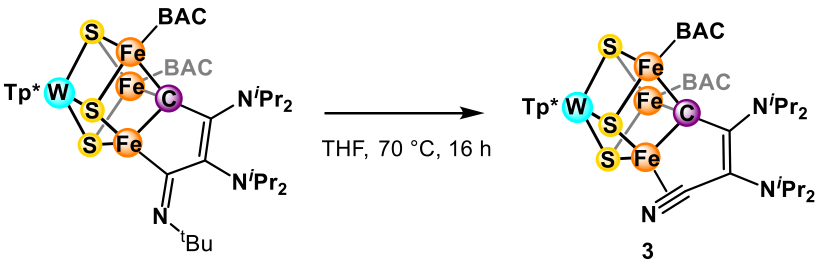

2-^{t}Bu **3**

Synthesis of **3**. In a glovebox, crude **2-^{t}Bu** (300.0 mg, 0.195 mmol) was added to a Schlenk tube and dissolved in THF (6 mL). The tube was capped, taken out of the box and heated in an oil bath at 70 °C for 16 h. The tube must be closed while heated to give **3** (***NOTE: heating a closed system can lead to an explosion, so make sure the amount of solvent is much smaller than the flask volume and that the reaction does not boil). The tube was then cooled, brought back into the box and the solvent removed *in vacuo*. The resultant solid was triturated in pentane, washed with pentane and Et_2O, then redissolved in THF and crystallized by THF/pentane vapor diffusion to yield X-ray quality crystals. Yield: 148 mg (51%). The mother liquor still contains some **3** although less pure, but it can be used to prepare **4**. ^{1}H NMR (400 MHz, THF-h_8, solvent suppression) δ 14.89, 8.24, 6.91, 6.43, 5.16, 1.31, 1.07, 0.84, 0.59, 0.02, -0.58, -3.17, -3.48, -4.48. Anal. calcd (%) $C_{61}H_{106}BFe_3N_{13}S_3W \cdot THF$ (M_r = 1552.08): C, 50.30; H, 7.40; N, 11.73. Found: C, 50.17; H, 7.80; N, 11.04.

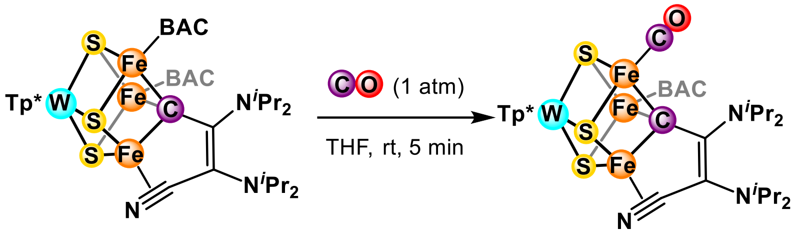

Synthesis of **4**. In a glovebox, **3** (132.0 mg, 0.089 mmol) was added to a Schlenk tube and dissolved in THF (5 mL). The tube was capped and degassed by three freeze-pump-thaw cycles on a Schlenk line. Then, the headspace of the tube was pressurized with 1 atm CO. The tube was capped again and inverted over a period of 5 minutes, after which the solution changed from green-brown to red-brown. NMR spectroscopy typically indicates the complete consumption of **3** at this point. The volatiles were removed *in vacuo* and the tube was brought back into the box. The resultant solid was washed with Et_2O then redissolved in THF to crystallize by THF/pentane vapor diffusion. Yield: 94.2 mg (83%). X-ray quality crystals can be grown by washing the crude material with C_6H_6, followed by vapor diffusion of Et_2O into a concentrated solution of **4** in THF. ^{1}H NMR (400 MHz, THF-h_8, solvent suppression) δ 10.18, 8.48, 7.24, 6.91, 6.81, 5.81, 5.58, 1.40, 1.23, 1.10, 1.06, 0.83, -0.28, -1.58, -3.73. Anal. calcd (%) $C_{47}H_{78}BFe_3N_{11}OS_3W$ (M_r = 1271.58): C, 44.39; H, 6.18; N, 12.12. Found: C, 44.49; H, 6.91; N, 11.15.

Synthesis of **4** with ^{13}CO. In a glovebox, **3** (20.0 mg, 0.013 mmol) was added to a 20 mL Schlenk tube with a stir bar and dissolved in THF (2 mL). The tube was capped and degassed by three freeze-pump-thaw cycles on a Schlenk line, capped tightly, then connected to one end of a glass solvent transfer bridge (as small as possible to minimize the amount of unused ^{13}CO), which is connected to the Schlenk line. The other end of the tube was connected to a ^{13}CO flask (~1 atm in 500 mL). The system was evacuated, then the solution was frozen in liquid nitrogen to prevent solvent contamination to the ^{13}CO flask. Then, the transfer bridge was closed to vacuum (similarly to a solvent vacuum transfer), and the ^{13}CO flask was opened to fill the system with ^{13}CO. The reaction tube was opened for about 5 minutes to fill the headspace with ^{13}CO while still frozen, then capped again and thawed while stirring vigorously. The solution changed from green-brown to red-brown after about 10 minutes. The tube was left to stir for 2 h, after which the volatiles were removed *in vacuo* and the tube was brought back into the box. The resultant solid was washed with Et_2O then redissolved in THF to crystallize by THF/pentane vapor diffusion. Yield: 7.1 mg (41%). NMR data are identical to **4** prepared from regular CO.

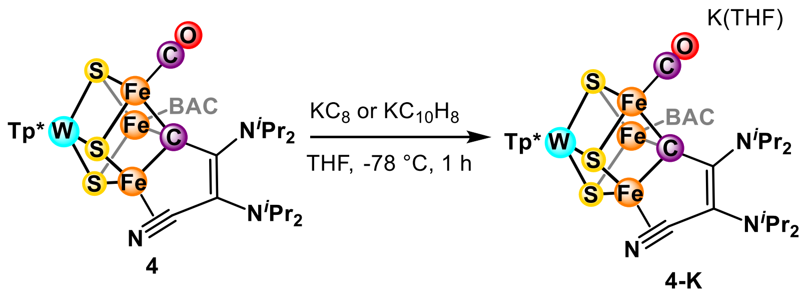

Synthesis of **4-K**. In a glovebox, **4** (20.0 mg, 0.016 mmol, 1 equiv) was dissolved in THF (2 mL) in a 20 mL scintillation vial with a pre-reduced Teflon stir bar to form a dark red-brown solution and cooled to -78 °C in the cold well. To this solution was added KC_8 (2.5 mg, 0.019 mmol, 1.2 equiv) or potassium naphthalenide (0.157 mL, 0.1 M in THF, 0.016 mmol, 1 equiv) and the dark green-brown reaction was stirred at -78 °C. After 2 h, the solution was filtered through Celite and the solvent removed *in vacuo*. The resultant solid was washed with Et_2O, then redissolved in THF and crystallized by THF/pentane vapor diffusion. Yield: 19 mg (88%). X-ray quality crystals were grown by vapor diffusion of Et_2O into a concentrated solution of **4-K** in THF. ^{1}H NMR (400 MHz, THF-h_8, solvent suppression) δ 15.24, 14.71, 10.86, 8.88, 6.43, 6.12, 5.13, 2.83, 2.42, 1.04, 0.79, 0.10, -0.42, -3.31, -8.89, -12.96. Anal. calcd (%) $C_{47}H_{78}BFe_3N_{11}OS_3WK{\cdot}THF$ (M_r = 1382.79): C, 44.30; H, 6.27; N, 11.14. Found: C, 44.82; H, 6.30; N, 10.96. The ^{13}CO-labeled version was prepared identically from ^{13}CO-labeled **4** for IR spectroscopy.

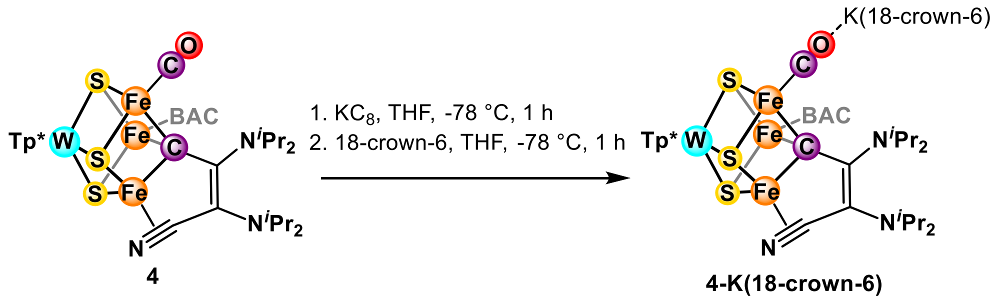

Synthesis of **4-K(18-crown-6)**. In a glovebox, **4** (12.8 mg, 0.010 mmol, 1 equiv) was dissolved in THF (2 mL) in a 20 mL scintillation vial with a pre-reduced Teflon stir bar to form a dark red-brown solution and cooled to -78 °C in the cold well. To this solution was added excess KC_8 (2.3 mg, 0.020 mmol, 2 equiv) and the dark green-brown reaction was stirred at -78 °C. After 2 h, IR spectroscopy indicated the disappearance of the starting material, and excess 18-crown-6 (5.4 mg, 0.020 mmol, 2 equiv) was added to the reaction. The solution was stirred at -78 °C for another 2 h before taking an aliquot for IR spectroscopy and concentrated under vacuum, then filtered through Celite and crystallized by THF/pentane vapor diffusion. Yield: 10 mg (64%). X-ray quality crystals were grown by vapor diffusion of Et_2O into a concentrated solution of **4-K(18-crown-6)** in DME. ^{1}H NMR (400 MHz, THF-h_8, solvent suppression) δ 19.10, 11.39, 9.07, 6.44, 6.04, 5.41, 2.94, 2.54, 1.07, 0.84, 0.10, -0.64, -3.82, -9.78, -14.87. Anal. calcd (%) $C_{59}H_{102}BFe_3KN_{11}O_7S_3W$ (M_r = 1575.00): C, 44.99; H, 6.53; N, 9.78. Found: C, 43.14; H, 6.31; N, 9.57. The ^{13}CO-labeled version was prepared identically from ^{13}CO-labeled **4**.

The reaction was also carried out identically using [2.2.2]cryptand instead of 18-crown-6 for IR spectroscopy, which shows the same C-O stretch.

The following clusters were not discussed in the main text but were also prepared/isolated to provide more support for structural assignment or reactivity pattern.

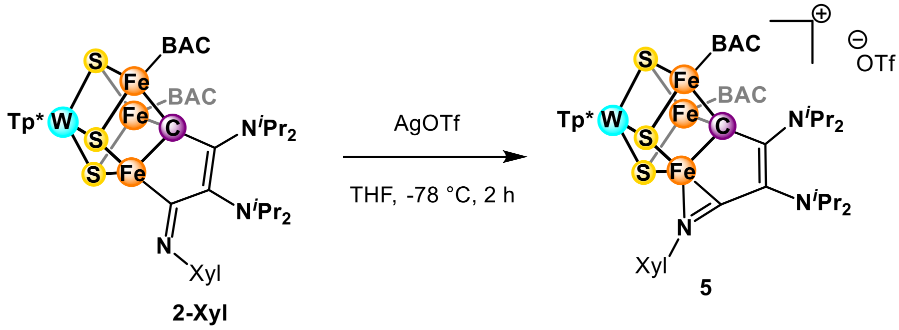

Synthesis of **5**. In a glovebox, **2-Xyl** (52.1 mg, 0.033 mmol, 1 equiv) was dissolved in THF (2 mL) in a 20 mL scintillation vial with a stir bar to form a dark green-brown solution and cooled to -78 °C in the cold well. To this solution was added AgOTf (8.4 mg, 0.033 mmol, 1 equiv) and the dark red-brown reaction was stirred at -78 °C. After 2 h, the solution was filtered through Celite and the solvent removed *in vacuo*. The resultant solid was washed with Et_2O, then redissolved in THF and crystallized by THF/pentane vapor diffusion. Yield: 40 mg (70%). X-ray quality crystals were grown by vapor diffusion of $^{i}Pr_2O$ into a concentrated solution of **5** in THF. ^{1}H NMR (400 MHz, THF-h_8, solvent suppression) δ 13.29, 11.09, 10.22, 7.33, 6.97, 6.38, 5.88, 5.75, 4.99, 1.08, 0.38, -0.27, -1.10, -1.88. Anal. calcd (%) $C_{70}H_{115}BFe_3N_{13}S_4F_3O_3W$ (M_r = 1734.20): C, 48.48; H, 6.68; N, 10.50. Found: C, 48.71; H, 6.60; N, 12.91.

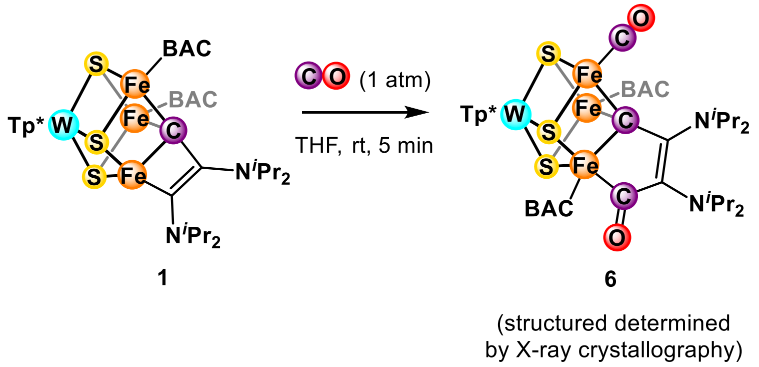

Reaction of **1** *with 1 atm CO*. Treatment of **1** with 1 atm CO results in a complex reaction mixture by ^{1}H NMR spectroscopy (see SI), but one product could be characterized by crystallography. In a glovebox, **1** (20.0 mg, 0.014 mmol) was dissolved in THF (0.7 mL) and transferred to a J. Young NMR tube. The tube was capped and degassed by three freeze-pump-thaw cycles on a Schlenk line. Then, the headspace of the tube was pressurized with 1 atm CO. The tube was capped again and inverted over a period of 5 minutes, after which NMR spectroscopy indicated the complete consumption of **1** and the appearance of new peaks between -2 and -12 ppm. The tube was brought back into the glovebox and the solvent was removed *in vacuo* to yield a dark film. The film was

washed with pentane and the product was extracted into Et_2O and filtered through a pad of Celite before the solvent was removed *in vacuo*. The resultant material was redissolved in a minimal amount of Et_2O and placed at -35 °C for several days to yield X-ray quality crystals, whose structure is determined to be **6**. Despite multiple trials, only a few crystals of **6** were observed each time, which precludes bulk characterization.

For all the reactions that result in products that can be isolated and characterized, spectroscopic yields were also measured by NMR spectroscopy. Each reaction was carried out on a small scale, and a known amount of an internal standard (4-phenylbenzaldehyde or cobaltocene) was added at the end of the reaction mixture without working up. Separately, a known amount of the same internal standard was added to a known amount of purified material. Comparison of the integrations between a pair of non-overlapping peaks (one each for the standard and the analyte) in both cases allows for the determination of reaction yields by NMR spectroscopy. The table below displays the results.

Table S1: Measured NMR spectroscopic yields for reactions

**Product**	**Spectroscopic yield/%**	**Isolated yield/%**	**Standard**
**2-^{t}Bu**	98	Quant	4-phenylbenzaldehyde
**2-Xyl**	94	85	4-phenylbenzaldehyde
**3**	61	51	4-phenylbenzaldehyde
**4**	87	83	4-phenylbenzaldehyde
**4-K**	90	88	cobaltocene
**4-K(18-crown-6)**	92	64	cobaltocene
**5**	93	70	4-phenylbenzaldehyde

3. NMR spectra:

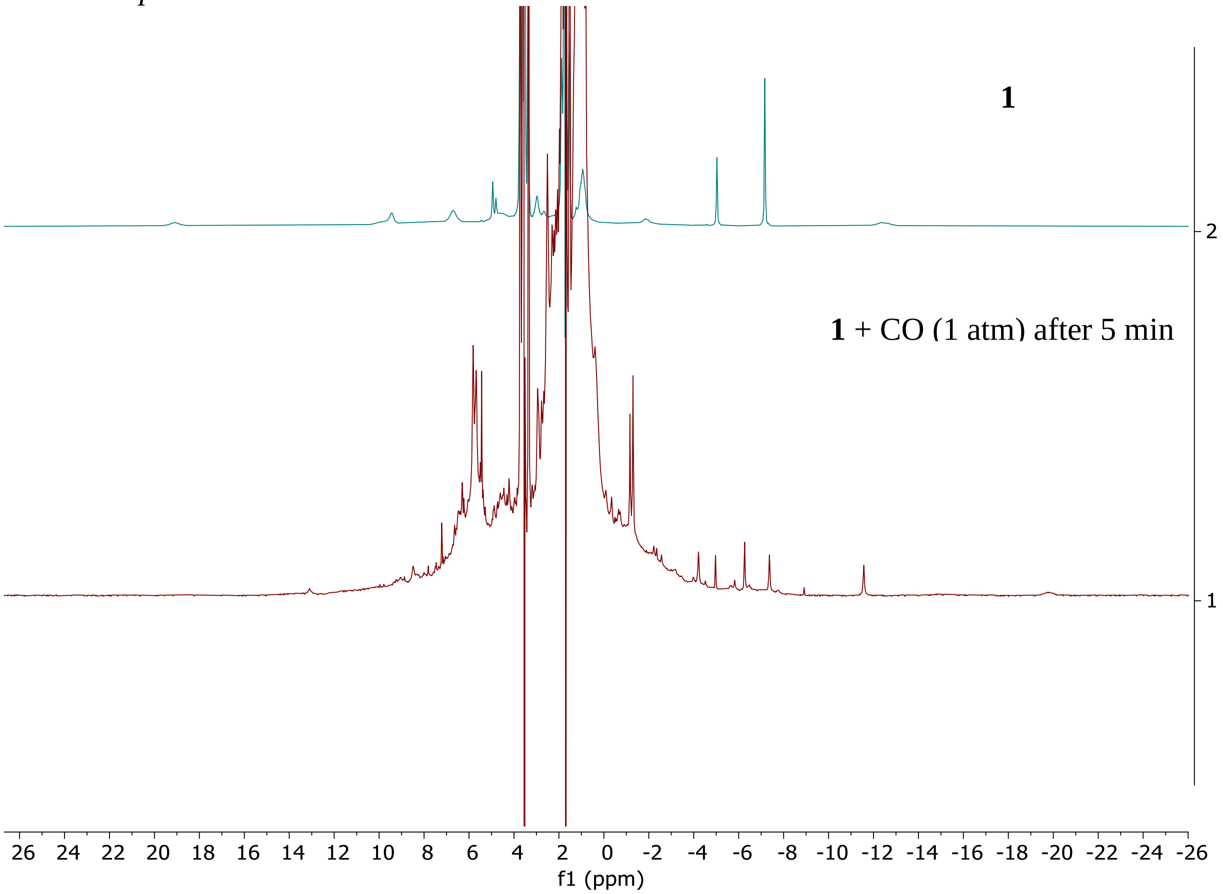

Figure S1: ^{1}H NMR spectrum (400 MHz, THF-h_8, solvent suppression) of **1** (top) and **1** + CO (1 atm) after mixing for 5 min (bottom). The peaks corresponding to the starting material disappears and new peaks appear within the -2 to -12 ppm region, assigned to **6** (structure below as determined by X-ray crystallography).

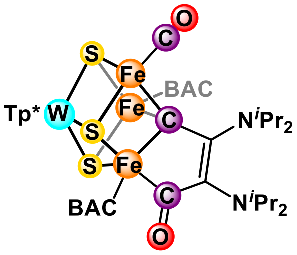

6

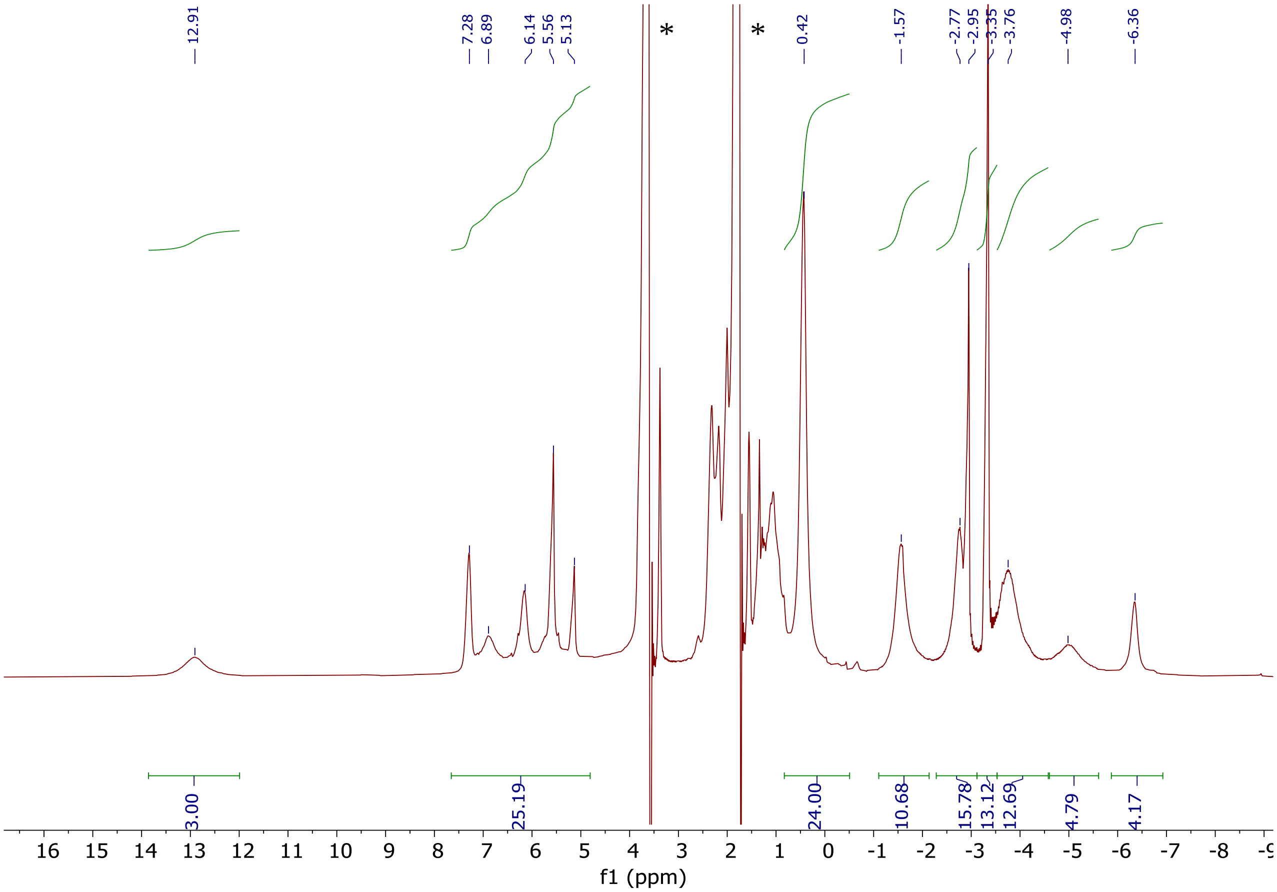

Figure S2: ^{1}H NMR spectrum (400 MHz, THF-h_8, solvent suppression) of **2-^{t}Bu**. Solvent peaks are indicated by asterisks (*).

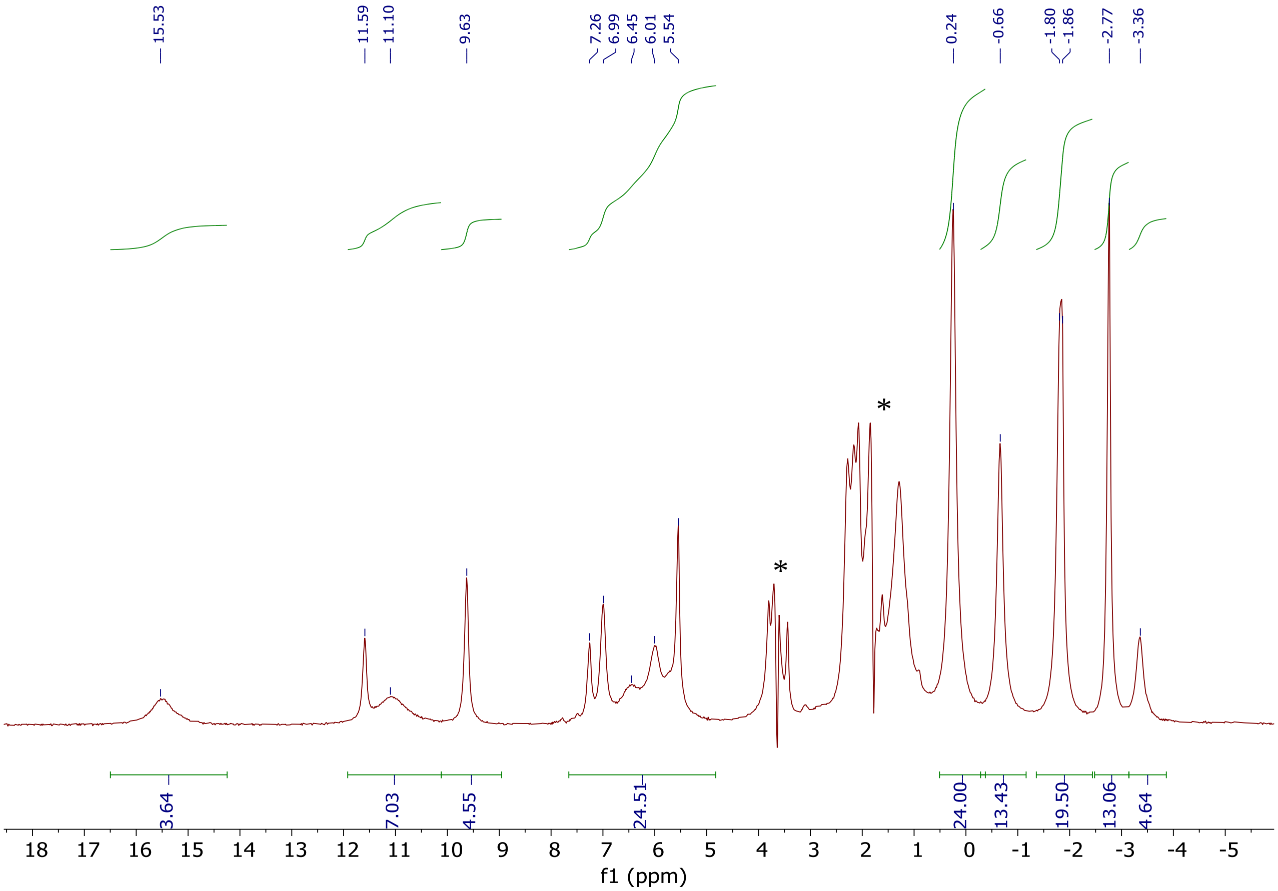

Figure S3: ^{1}H NMR spectrum (400 MHz, THF-h_8, solvent suppression) of **2-Xyl**. Solvent peaks are indicated by asterisks (*).

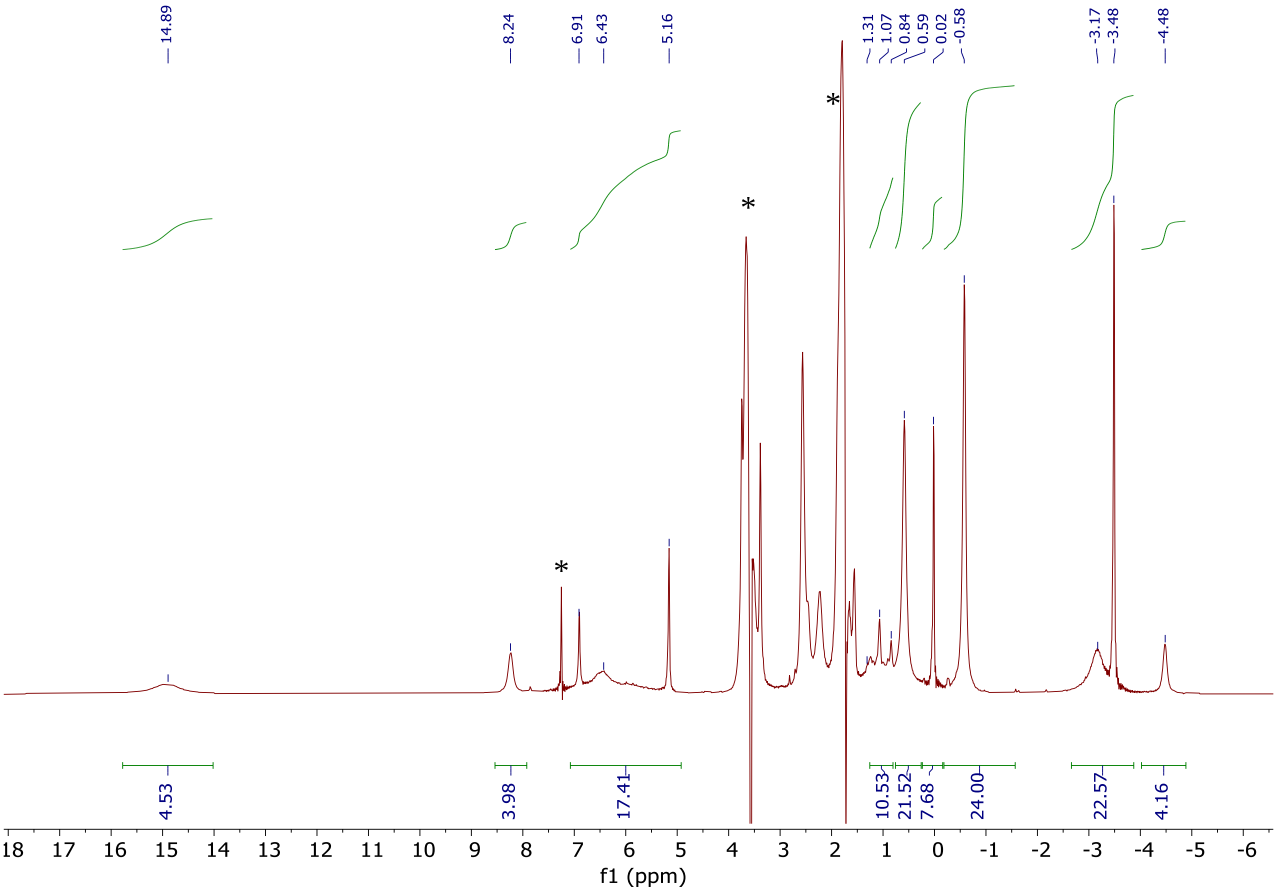

Figure S4: ^{1}H NMR spectrum (400 MHz, THF-h_8, solvent suppression) of **3**. Solvent peaks are indicated by asterisks (*).

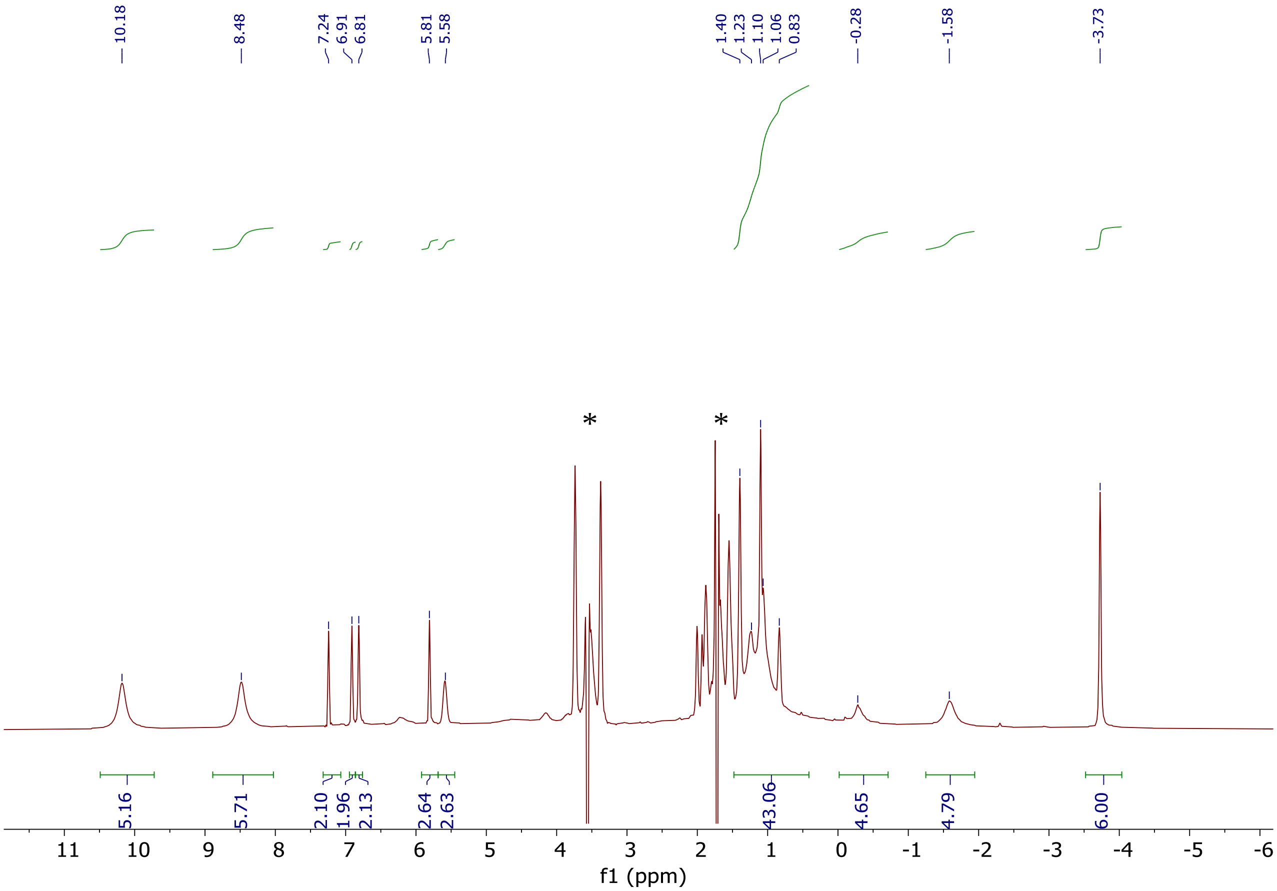

Figure S5: ^{1}H NMR spectrum (400 MHz, THF-h_8, solvent suppression) of **4**. Solvent peaks are indicated by asterisks (*).

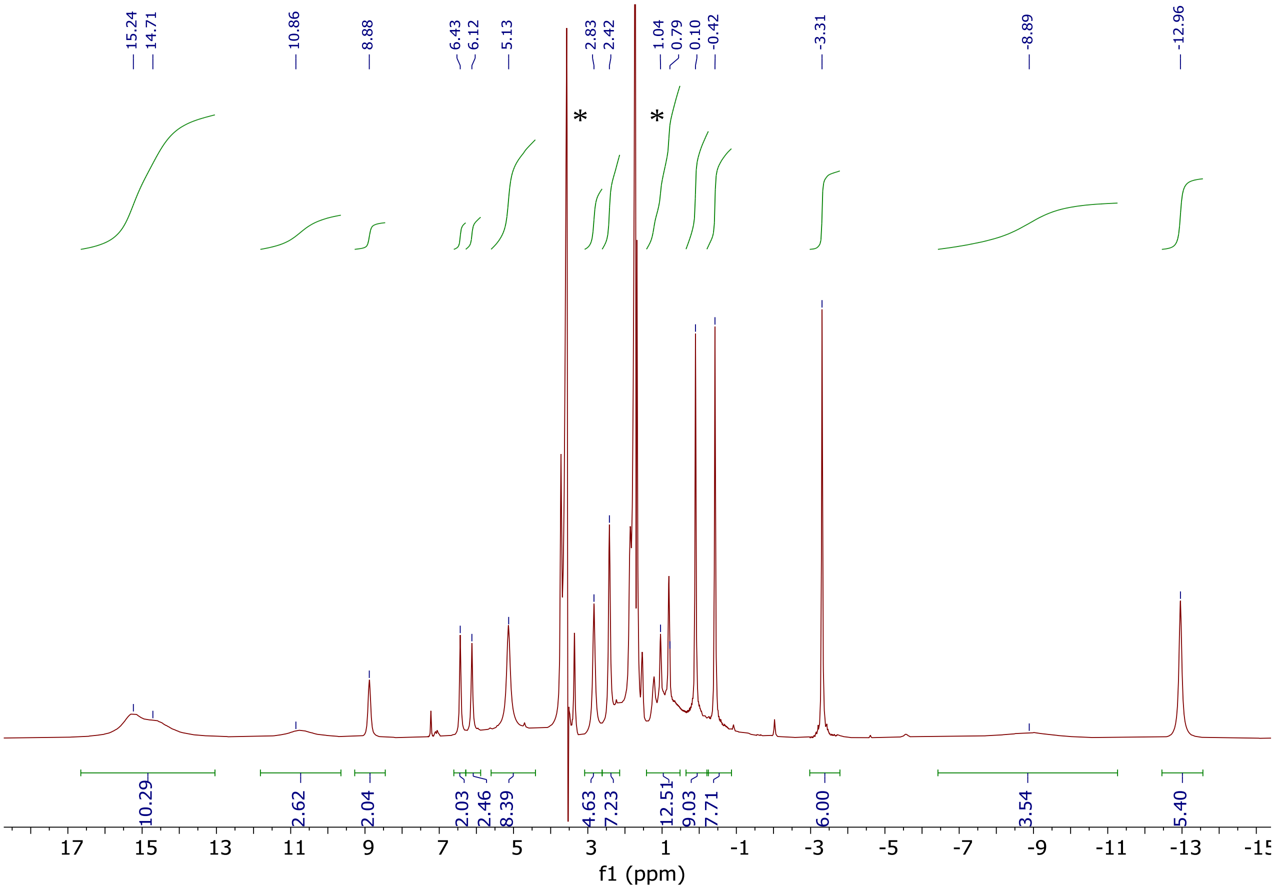

Figure S6: ¹H NMR spectrum (400 MHz, THF-h_8, solvent suppression) of **4-K**. Solvent peaks are indicated by asterisks (*).

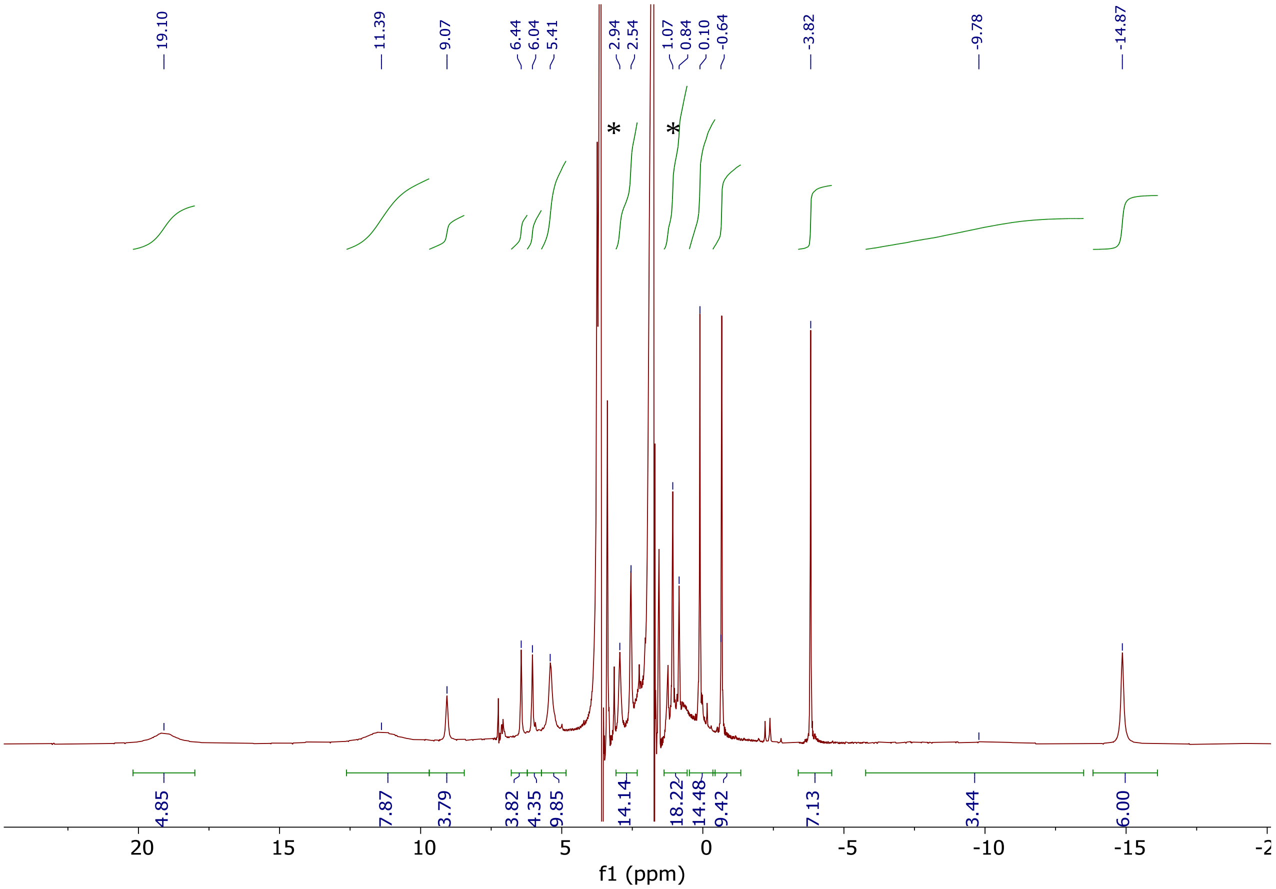

Figure S7: ^{1}H NMR spectrum (400 MHz, THF-h_8, solvent suppression) of **4-K(18-crown-6)**. Solvent peaks are indicated by asterisks (*).

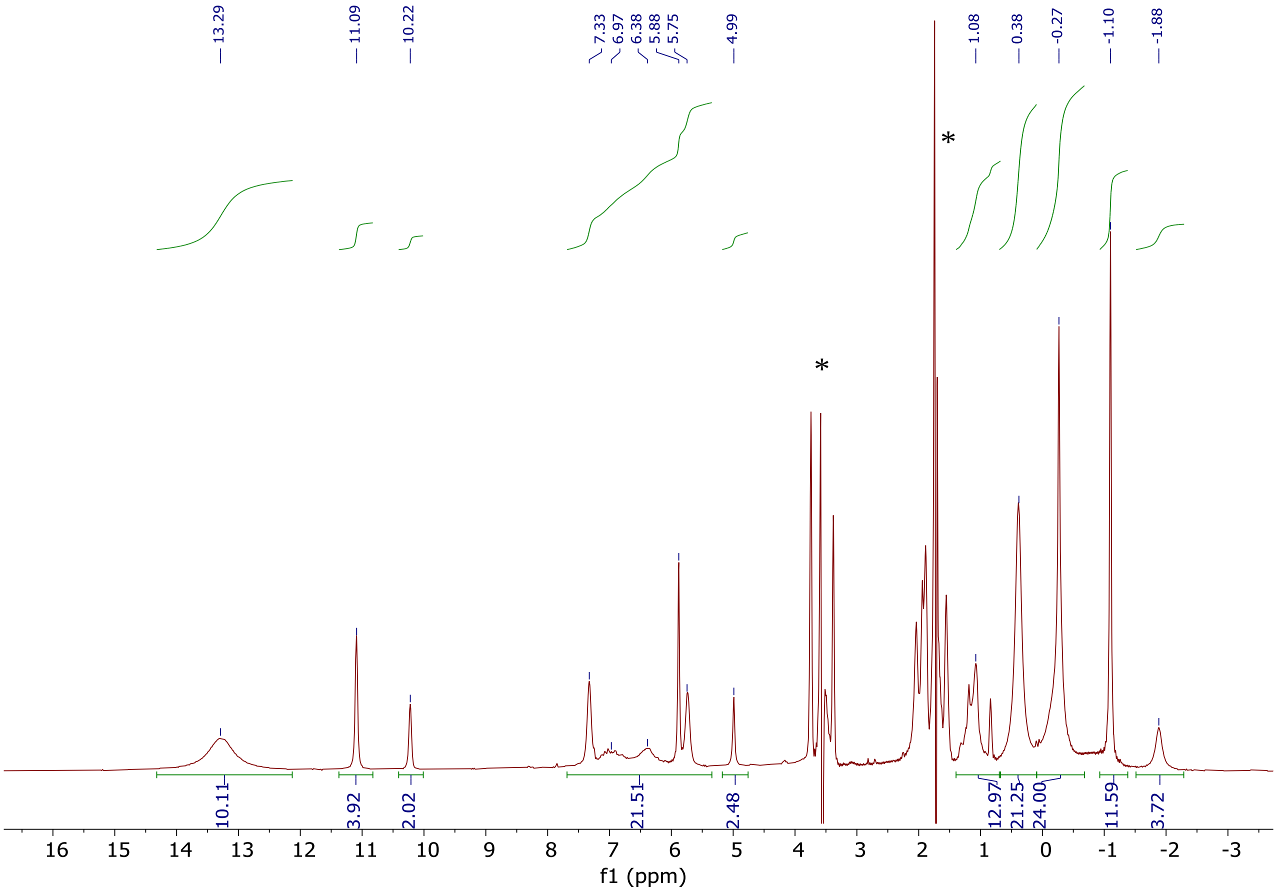

Figure S8: ^{1}H NMR spectrum (400 MHz, THF-h_8, solvent suppression) of **5**. Solvent peaks are indicated by asterisks (*).

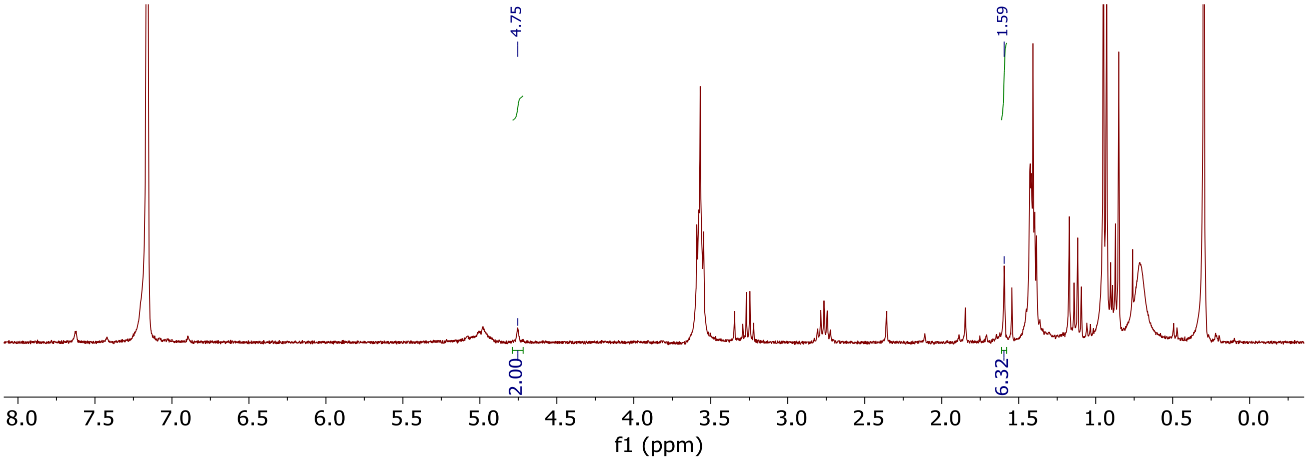

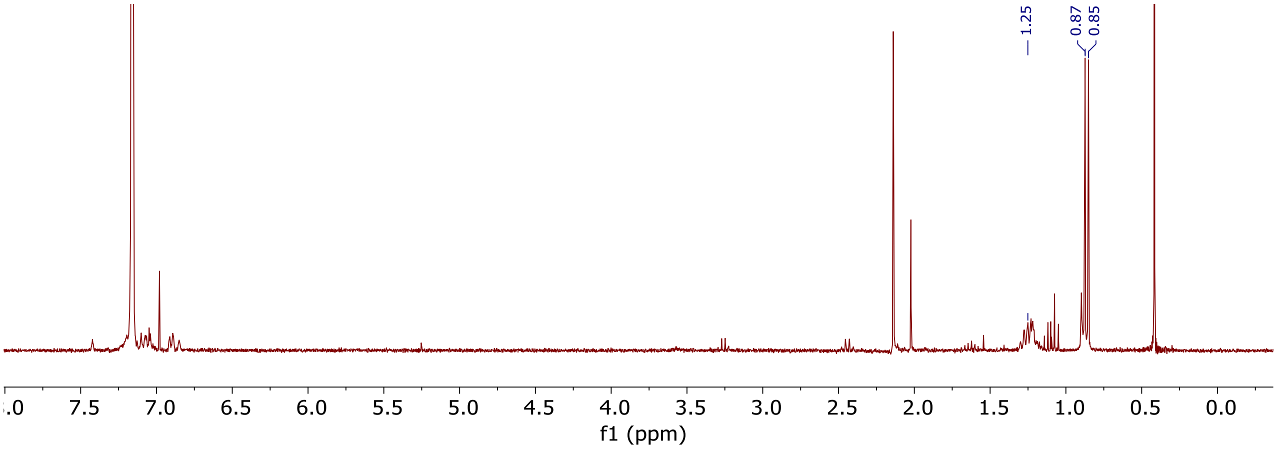

Figure S9: Top: A sample of **2-^{t}Bu** (25 mg) in C_6D_6 (0.7 mL) was heated at 70 °C for 16 h, after which the entire content was vacuum transferred into an empty J. Young tube cooled in liquid N_2. The ^{1}H NMR spectrum (300 MHz, C_6D_6) of the J. Young tube was recorded, where the labeled peaks are assigned to isobutene, but isobutane cannot be identified due to the various side products due to undesired reactions. Bottom: A sample of **2-^{t}Bu** (40 mg) in xylenes (5 mL) was heated at 70 °C for 16 h (the reaction is somewhat cleaner when diluted, to avoid side products when vacuum transferred), after which the reaction flask was cooled to 0 °C to minimize xylenes transfer, and the volatiles were vacuum transferred onto a J. Young tube containing degassed C_6D_6 (0.7 mL) cooled in liquid N_2. The ^{1}H NMR spectrum (300 MHz, C_6D_6) of the J. Young tube was recorded, where the labeled peaks are assigned to isobutane. Isobutene was not observed, which could be because it still remained in the original reaction flask, as only part of the reaction products could be transferred.

*4. Literature comparison and IR spectroscopy supporting side-on nitrile assignment for **3**:*

An η^2-iminoacyl motif is expected to be substantially bent at C18, with literature examples around 130° (see Figure S20 for crystal structure and atom labels).[2] Side-bound organic nitriles are also significantly bent at C, but typically have more obtuse angles in non-chelated versions.[3] Chelated nitriles show much larger angles, above 140° with a Ru example of the same size chelate as **3** displaying a similarly obtuse angle (167.7°).[3] The C-N bond length of 1.205(6) Å is shorter than observed for iminoacyl (1.25 Å),[2] and in the range typically observed for side-on nitriles. **3** displays a very similar C-N distance to the one observed (1.194(4) Å) in the only previously structurally characterized Fe analog.[4] This C-N distance, elongated from free nitrile (1.16 Å for CH_3CN),[5] is indicative of significant π-backbonding from Fe.

We have also prepared **5** from **2-Xyl** as discussed above. Treatment of **2-Xyl** with AgOTf in THF at -78 °C leads to the clean formation of **5** as determined by XRD. The iminoacyl moiety in **5** coordinates to Fe in an η^2 manner, with the C-N distance of 1.266(7) Å within the range (1.26 – 1.28 Å) of previously reported complexes bearing η^2-iminoacyl moieties.[6–9] Moreover, this C-N distance is longer than in **3** (1.205(6) Å), consistent with a higher bond order in **3** and further supporting its assignment as a nitrile. Iminoacyl ligands coordinate to metals in η^1 or η^2 fashion depending on the nature of the metal center. [6,7,10] In **5**, the decrease in electron density at the iminoacyl-bound Fe center upon oxidation promotes binding of the N lone pair.

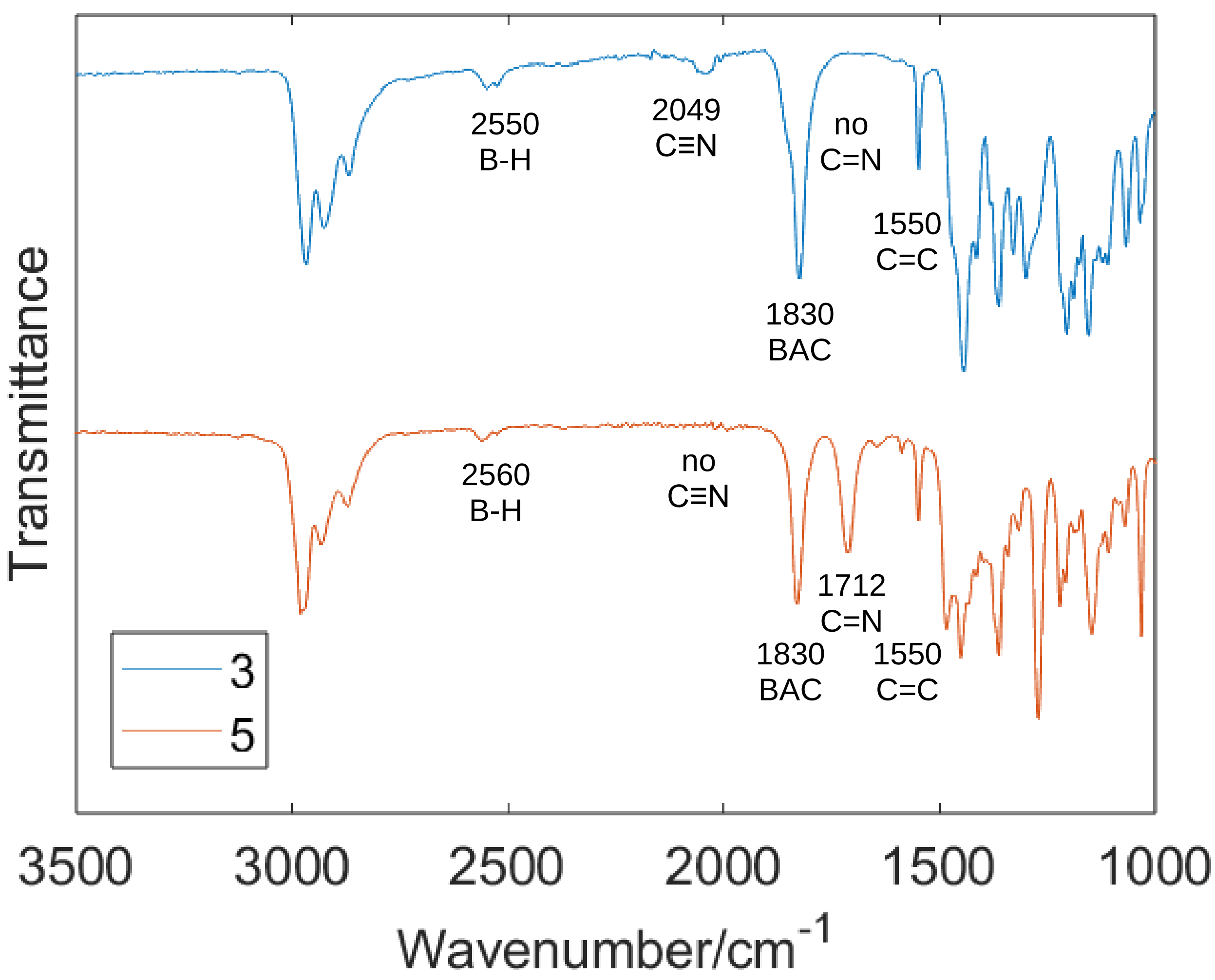

Figure S10: ATR-IR spectra of **3** (top, blue) and **5** (bottom, orange).

The IR spectra of **3** and **5** (thin film, ATR mode, Figure S10) are consistent with the structural assignments. For **3**, a feature at 2049 cm^{-1} assigned to the C≡N motif is observed.[3] In contrast, for **5**, no peak in this region is seen but instead a peak at 1712 cm^{-1} is present, assigned to the C=N motif.

5. Physical methods:

Mössbauer spectroscopy:

Zero field ^{57}Fe Mössbauer spectra were recorded in constant acceleration at 80 K on a spectrometer from See Co (Edina, MN) equipped with an SVT-400 cryostat (Janis, Woburn, MA). The quoted isomer shifts are relative to the centroid of the spectrum of α-Fe foil at room temperature. Samples were ground with boron nitride into a fine powder and transferred to a Delrin cup. The data were fitted to Lorentzian lineshapes using the program WMOSS (www.wmoss.org).

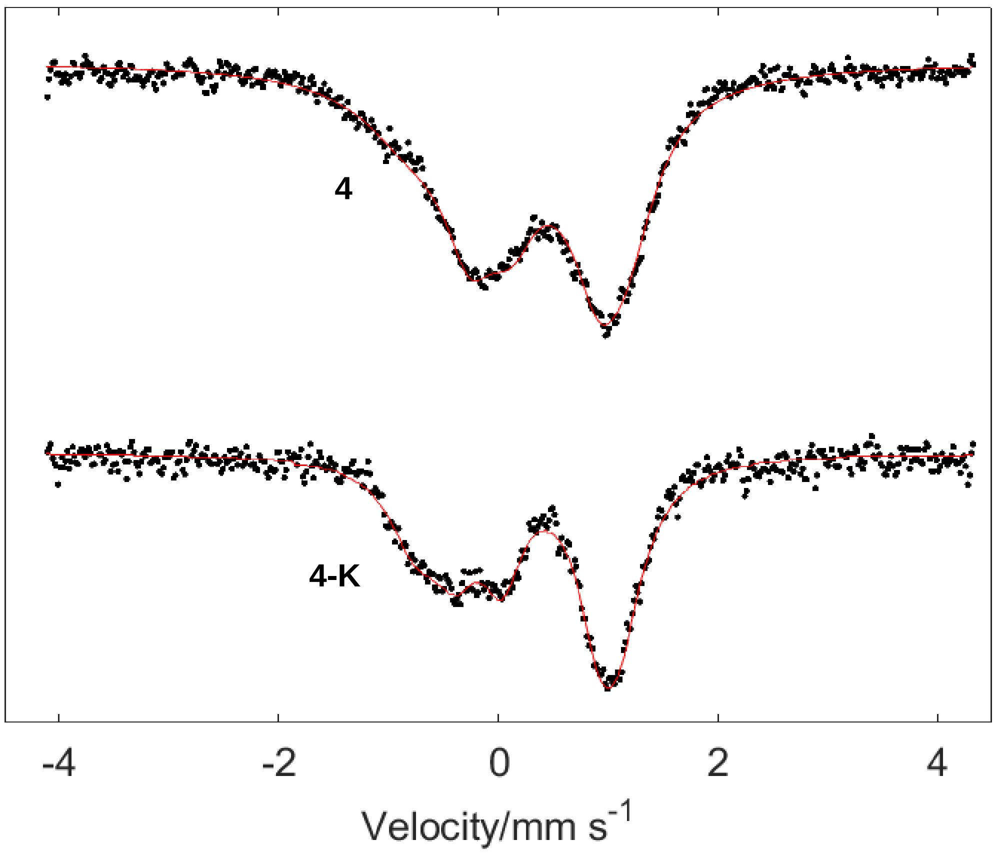

Figure S11: Mössbauer spectra of **4** and **4-K** (80 K, no applied field). Average isomer shifts: δ_{ave} = 0.33 mm s^{-1} for both clusters.

The Mössbauer spectra of **4** and **4-K** consist of broad quadrupole doublets (Figure 2), owing to valence delocalization on the Mössbauer timescale that does not resolve the individual Fe signals. This has been observed for other synthetic iron-sulfur clusters,[11,12] and because the broadness precludes the definite assignment of isomer shifts, we only present one set of values for the fit parameters. Other fits are possible for both systems.

Mössbauer fit parameters:

For **4**: The Mössbauer spectrum of **4** can be fit with a three-site model using the following parameters:

Site 1: δ = 0.02 mm s^{-1} $|E_Q|$ = 1.57 mm s^{-1} Linewidth = 1.14 mm s^{-1} Area = 33%

Site 2: δ = 0.65 mm s^{-1} $|E_Q|$ = 1.07 mm s^{-1} Linewidth = 0.64 mm s^{-1} Area = 33%

Site 3: δ = 0.33 mm s^{-1} $|E_Q|$ = 1.19 mm s^{-1} Linewidth = 0.56 mm s^{-1} Area = 33%

For **4-K**: The Mössbauer spectrum of **4-K** can be fit with a three-site model using the following parameters:

Site 1: δ = 0.47 mm s^{-1} $|E_Q|$ = 0.87 mm s^{-1} Linewidth = 0.45 mm s^{-1} Area = 33%

Site 2: δ = 0.17 mm s^{-1} $|E_Q|$ = 1.84 mm s^{-1} Linewidth = 0.60 mm s^{-1} Area = 33%

Site 3: δ = 0.35 mm s^{-1} $|E_Q|$ = 1.47 mm s^{-1} Linewidth = 0.54 mm s^{-1} Area = 33%

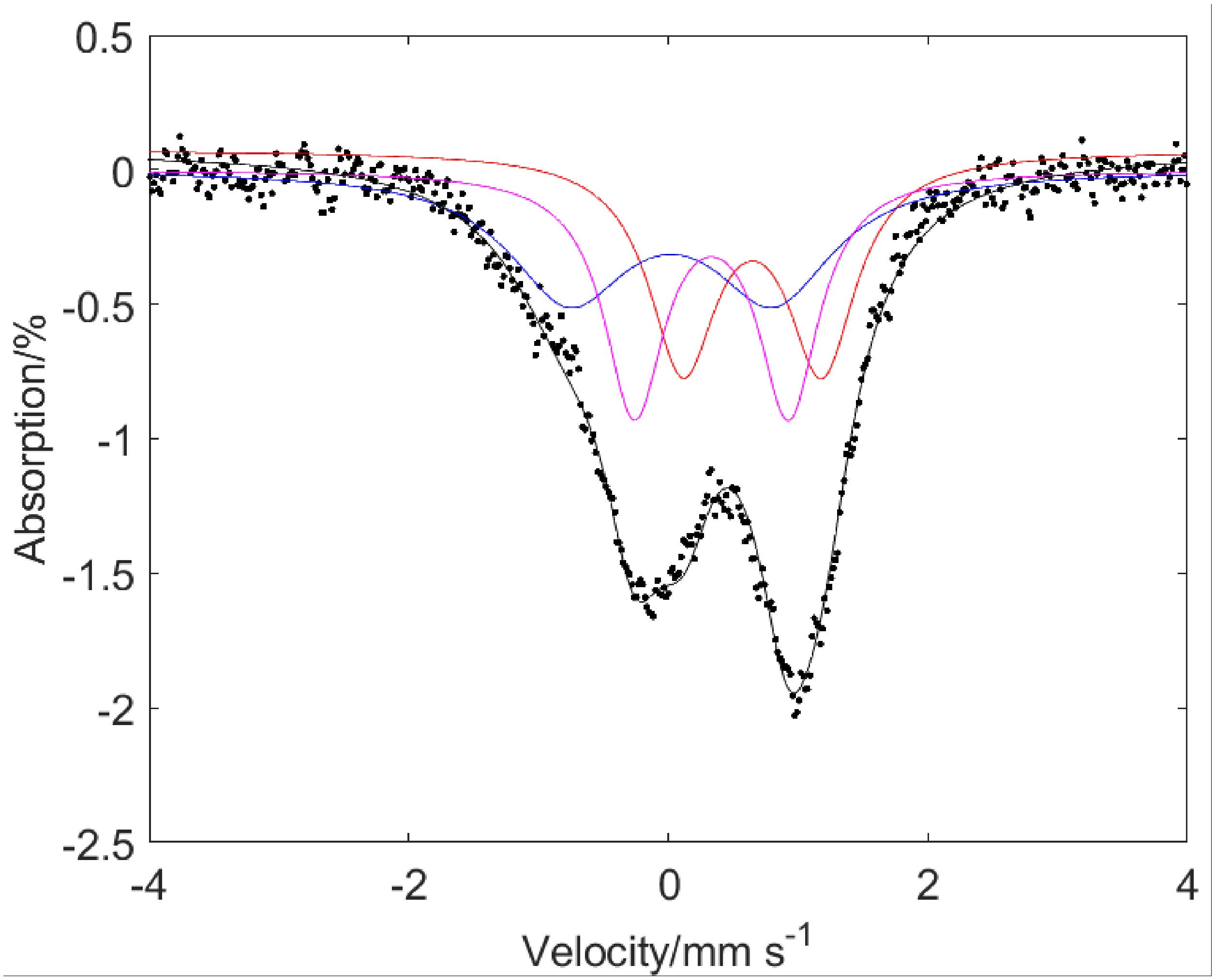

Figure S12: Fitting for the Mössbauer spectrum of **4** (80 K, no applied field) using a three-site model, with the total fit shown by the black trace.

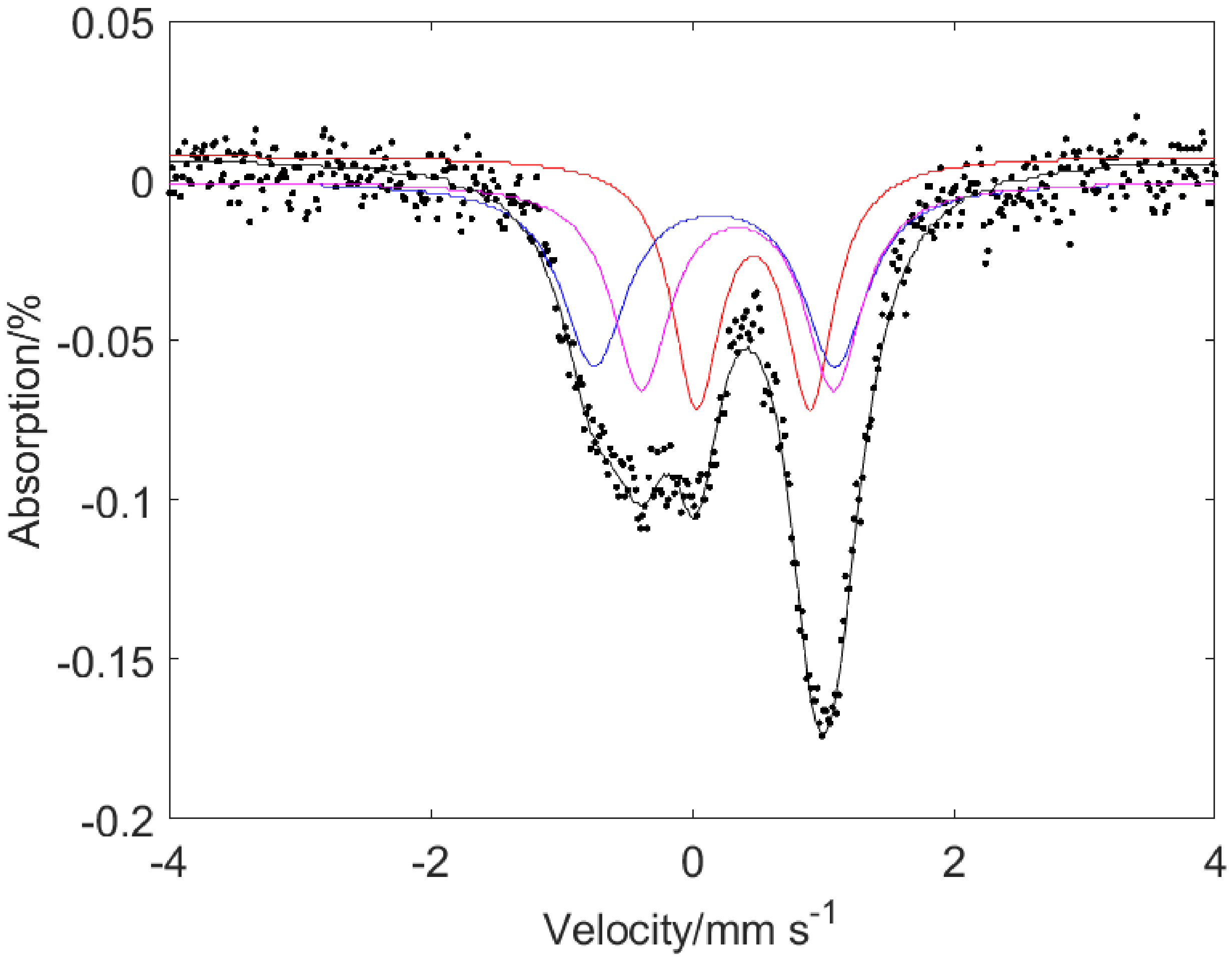

Figure S13: Fitting for the Mössbauer spectrum of **4-K** (80 K, no applied field) using a three-site model, with the total fit shown by the black trace.

Electrochemical measurements:

Cyclic voltammetry experiments were performed with a Pine Instrument Company AFCBP1 biopotentiostat with the *AfterMath* software package. All measurements were performed in a three-electrode cell, which consisted of glassy carbon (working; ⌀ = 3.0 mm), Ag wire (reference), and bare Pt wire (counter), in a N_2-filled MBraun glovebox at room temperature. Dry CH_3CN that contained ~0.2 M $[Bu_4N][PF_6]$ was used as the electrolyte solution. Redox potentials are reported relative to the ferrocene/ferrocenium redox wave (Fc^+/Fc; ferrocene added as an internal standard). The open circuit potential was measured prior to each voltammogram being collected. Voltammograms were scanned reductively in order to minimize the oxidative damage that was frequently observed on scanning more oxidatively.

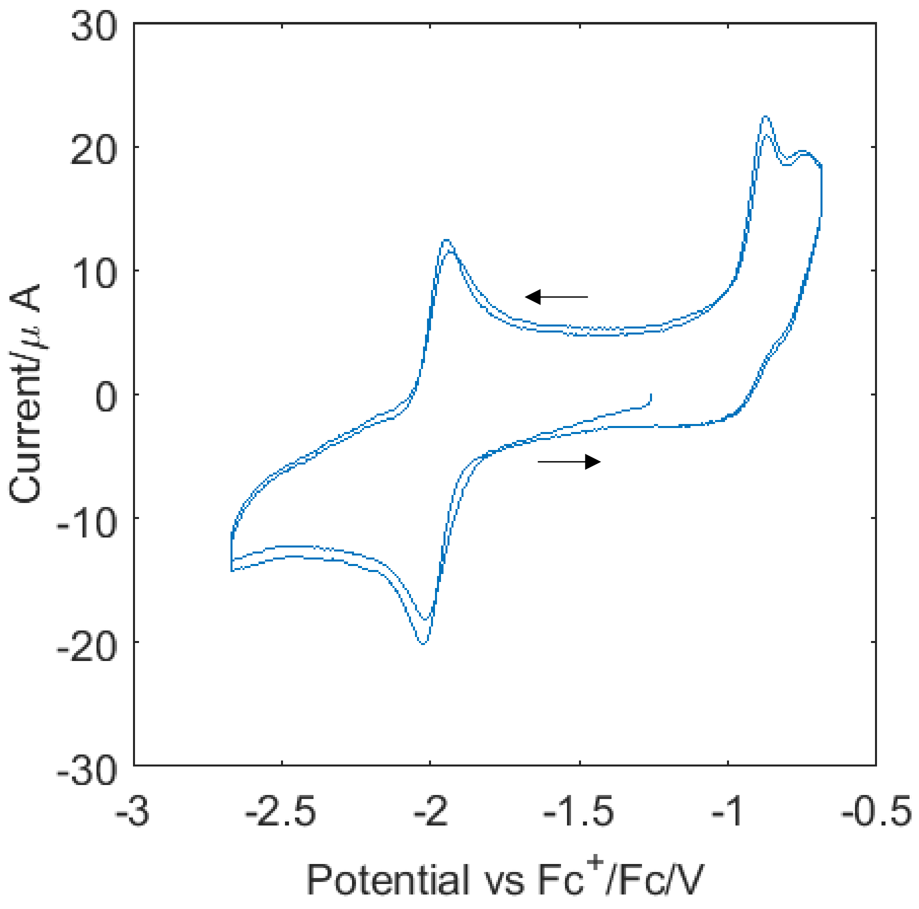

Figure S14: Cyclic voltammetry (CV) scan for **4**, starting from the open circuit potential, showing the reversible feature at -1.99 V vs. Fc^+/Fc. Conditions: 2.5 mM cluster in in MeCN with 0.2 M $TBAPF_6$, scan rates of 200 mV s^{-1}.

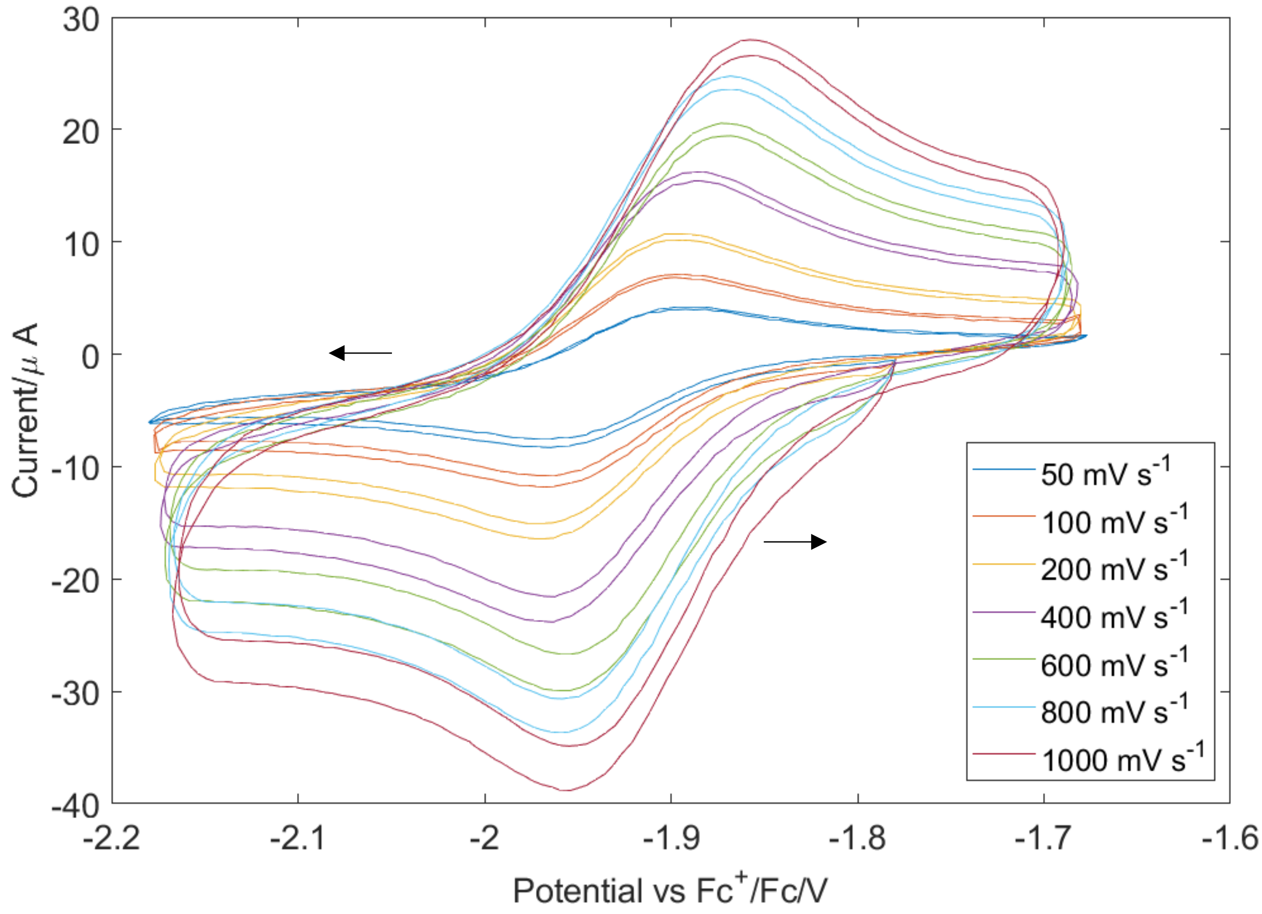

Figure S15: CV of **4** at different scan rates, showing the reversible redox event.

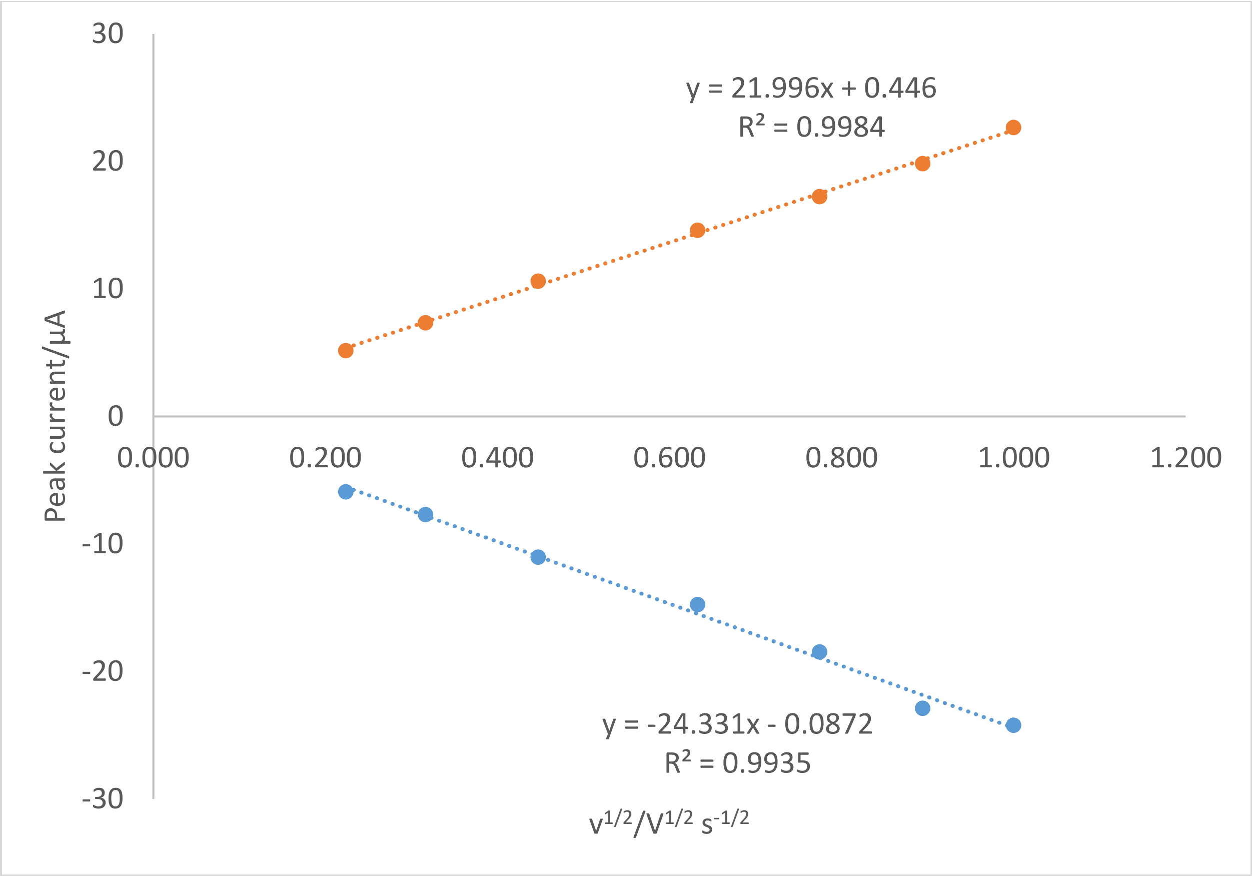

Figure S16: Peak current vs. square root of scan rate for the reversible redox feature in **4**.

Additional IR spectra:

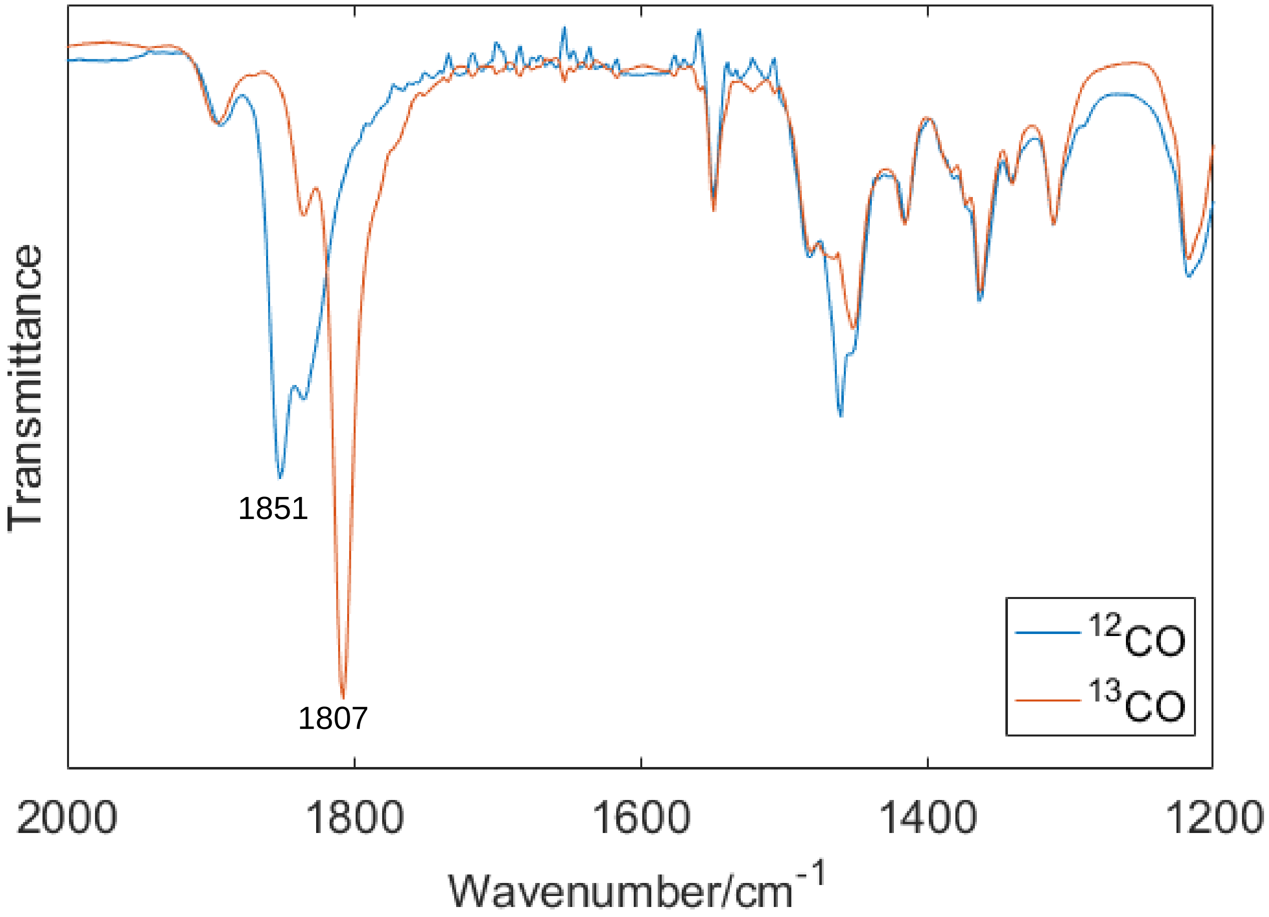

Figure S17: IR spectra of **4** in THF with ^{12}CO and ^{13}CO over a wider window, with the CO peaks indicated.

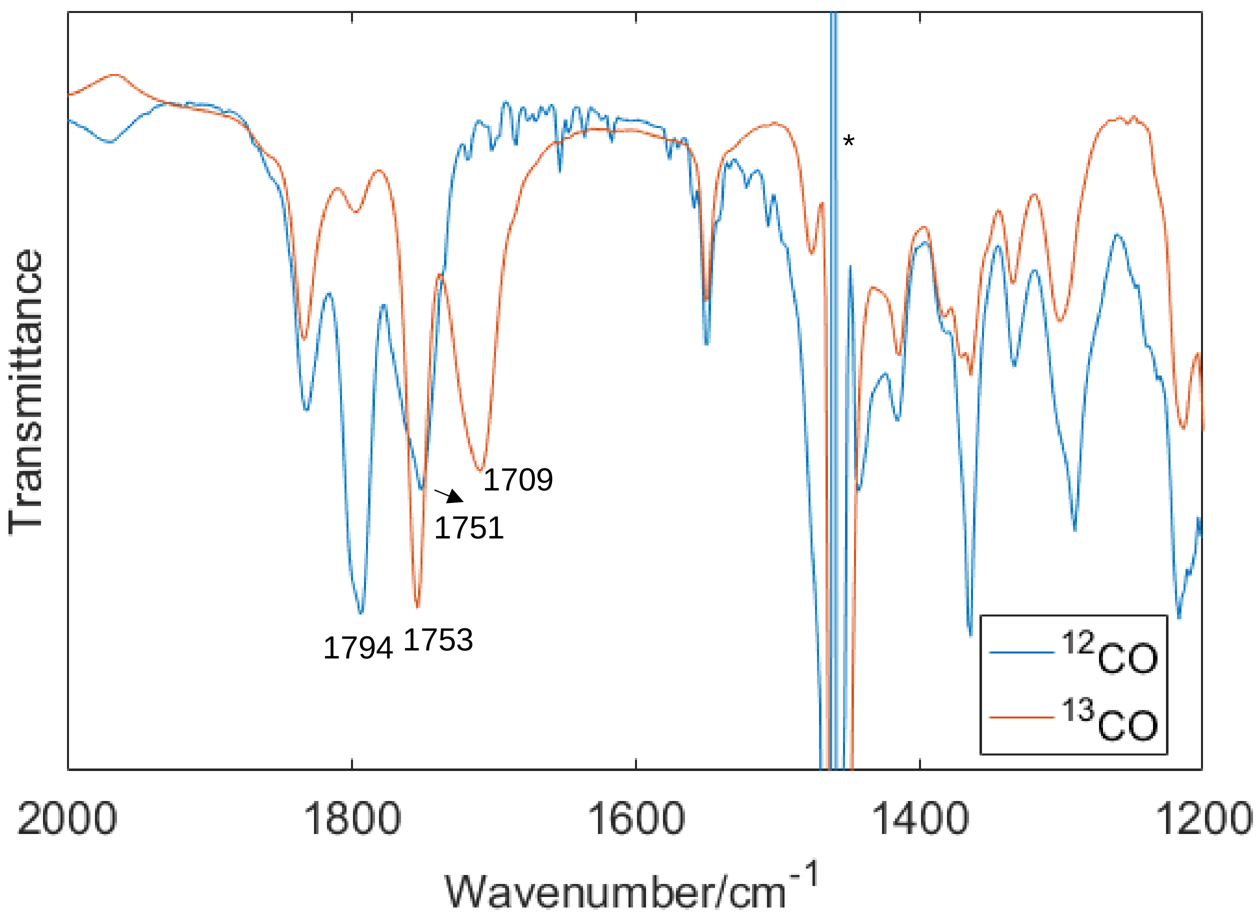

Figure S18: IR spectra of **4-K** in THF with ^{12}CO and ^{13}CO over a wider window, with the CO peaks indicated. The feature marked with an asterisk (*) is an artifact due to the solvent.

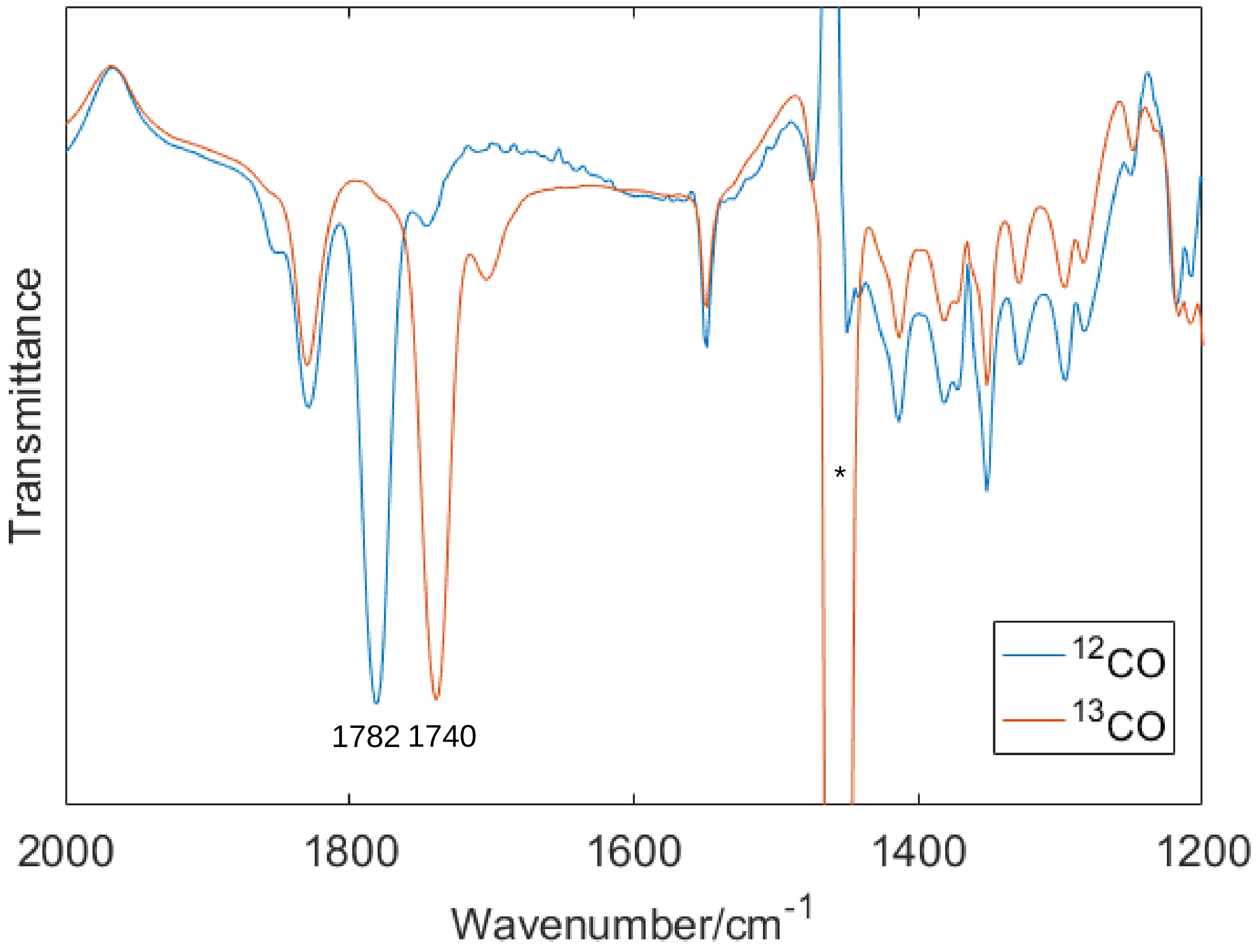

Figure S19: IR spectra of **4-K(18-crown-6)** in THF with ^{12}CO and ^{13}CO over a wider window, with the CO peaks indicated. The feature marked with an asterisk (*) is an artifact due to the solvent.

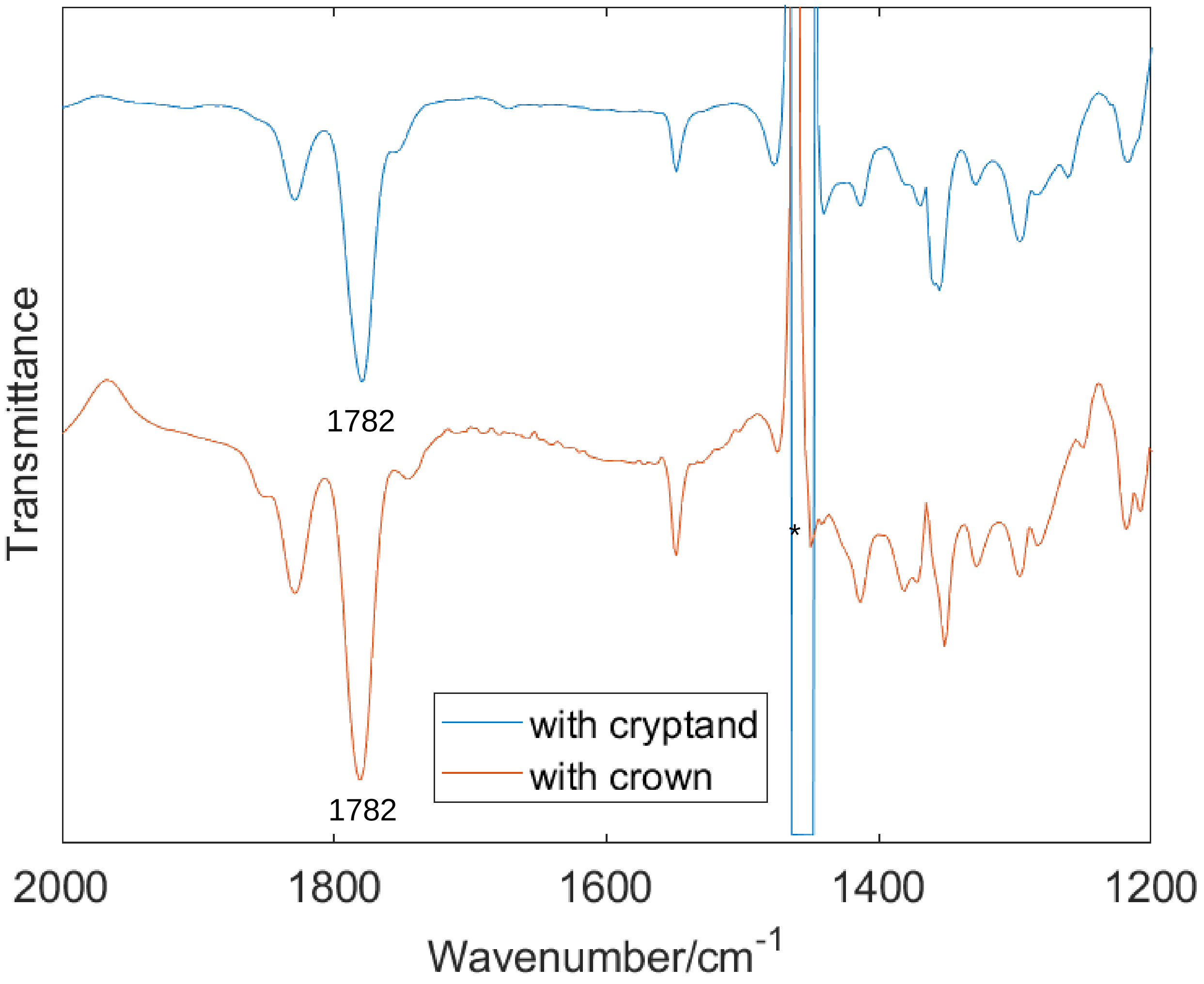

Figure S20: IR spectra of **4-K** in the presence of 18-crown-6 and [2.2.2]cryptand in THF, with the CO peaks indicated. The feature marked with an asterisk (*) is an artifact due to the solvent.

Table S2: Summary of CO stretching frequencies and comparison with calculated values

Cluster	ν_{12CO}/cm^{-1}	ν_{13CO}/cm^{-1} (exp)	ν_{13CO}/cm^{-1} (calc from harmonic oscillator model)
**4**	1851	1807	1810
**4-K**	1794, 1751	1753, 1709	1754, 1712
**4-K(18-crown-6)**	1782	1740	1742

Evans method for 4:

The magnetic susceptibility of **4** was measured using Evans method on a THF solution of the cluster with 3% added C_6H_6 as a reference between 25 °C and -100 °C. The variable-temperature data suggest that the cluster possesses a spin state of S = 1 (theoretical μ_{eff} = 2.83μ_B).

Table S3: Variable-temperature Evans method data for **4**

**Temperature/°C**	**Measured μ_{eff}/μ_B**
25	2.71
0	2.78
-20	2.71
-40	2.79
-60	2.72
-80	2.69
-100	2.61

EPR spectroscopy:

Samples were prepared as solutions (ca. 2 mM) in 2-MeTHF and rapidly cooled in liquid nitrogen to form a frozen glass. All X-band EPR experiments presented in this study were acquired at the Caltech EPR facility. X-band CW EPR spectra were acquired on a Bruker (Billerica, MA) EMX spectrometer using Bruker Xenon software (ver. 1.2). Temperature control was achieved using liquid helium and an Oxford Instruments (Oxford, UK) ESR-900 cryogen flow cryostat and an ITC-503 temperature controller. Spectra were simulated using EasySpin5 (release 5.2.35)[13] with Matlab R2021b.

EPR spectroscopy was employed to determine the spin state of odd-electron clusters **4-K** and **4-K(18-crown-6)**. Both species possess a spin state of S = 3/2, with very similar spectra.

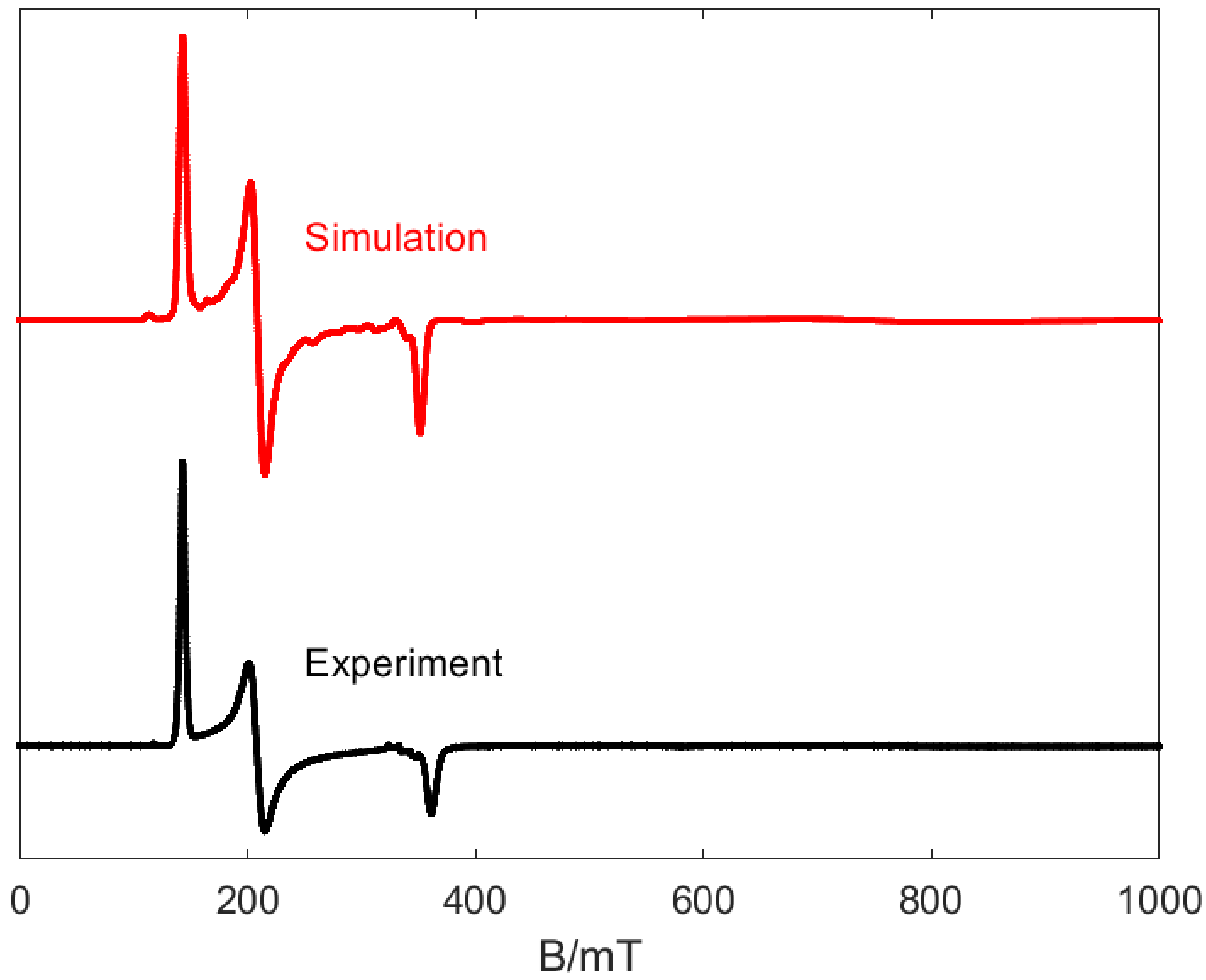

Figure S21: X-band EPR spectrum of **4-K** as a frozen glass in 2-MeTHF at 5 K. Acquisition parameters: frequency = 9.64 MHz, power = 2.18 mW, conversion time = 10 ms, modulation amplitude = 8 G. Simulation parameters: S = 3/2, g = 2.05, large D (D = 2 cm^{-1}), E/D = 0.13, DStrain = 0.047 cm^{-1}.

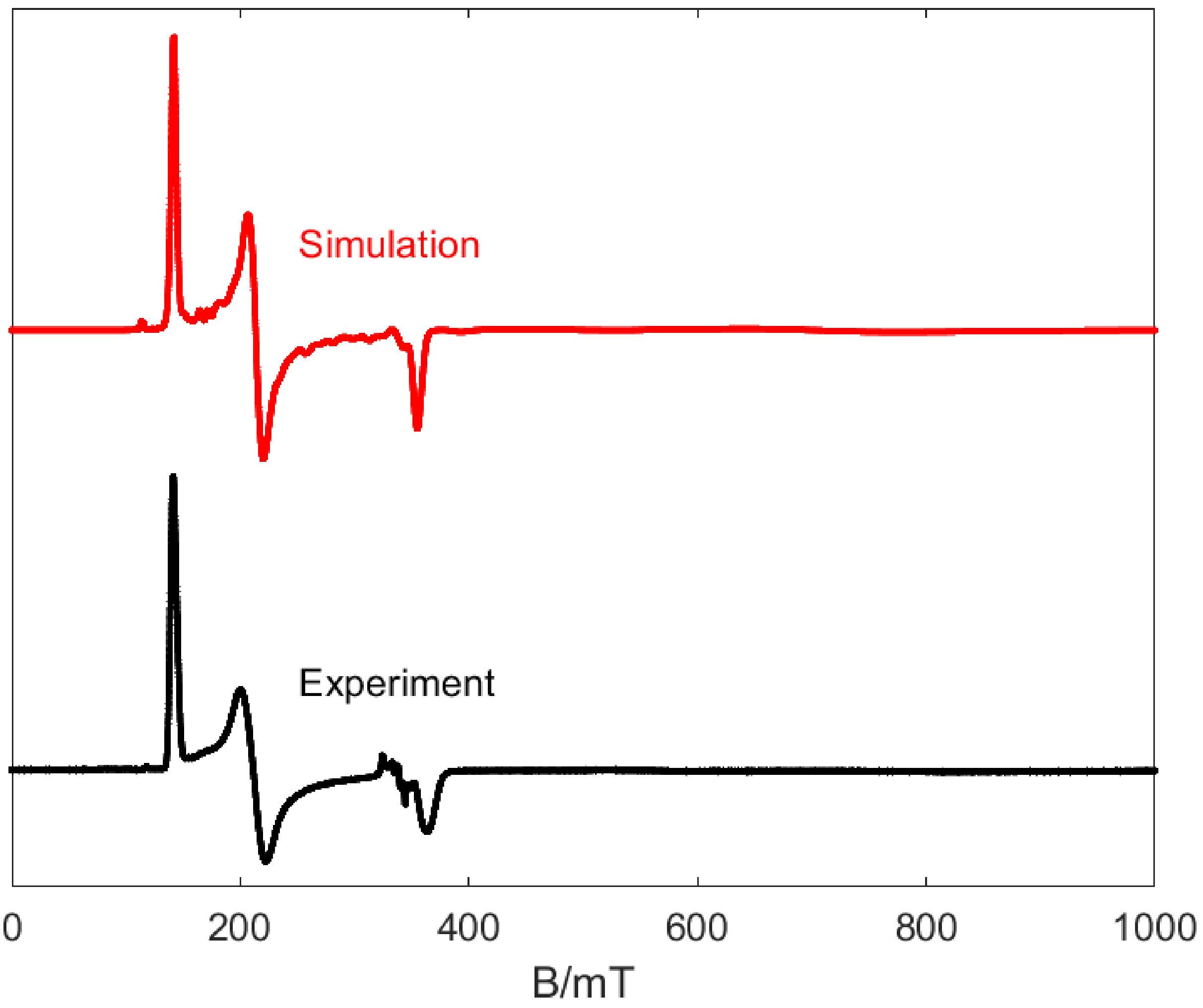

Figure S22: X-band EPR spectrum of **4-K(18-crown-6)** as a frozen glass in 2-MeTHF at 5 K. Acquisition parameters: frequency = 9.64 MHz, power = 2.18 mW, conversion time = 10 ms, modulation amplitude = 8 G. Simulation parameters: S = 3/2, g = 2.05, large D (D = 2 cm^{-1}), E/D = 0.14, DStrain = 0.054 cm^{-1}.

Pulse EPR spectroscopy. All pulse EPR and electron nuclear double resonance (ENDOR) experiments were acquired using a Bruker (Billerica, MA) ELEXSYS E580 pulse EPR spectrometer. All X-band data was acquired using a Bruker MD-4 resonator. Temperature control was achieved using an Oxford Instruments CF935 and Mercury ITC.

X-band HYSCORE spectra were acquired using the 4-pulse sequence ($\pi/2 - \tau - \pi/2 - t_1 - \pi - t_2 - \pi/2$ – echo), where τ is a fixed delay, while t_1 and t_2 are independently incremented by Δt_1 and Δt_2, respectively. At each field, the fixed delay τ was selected to be a multiple of the time interval equivalent to the inverse of the ^{1}H Larmor frequency, in order to selectively suppress contributions from solvent matrix protons. A 16-step phase cycle was used to eliminate contributions from secondary/tertiary spin echoes and associated artifacts in the time domain. The time domain data was baseline-corrected (third-order polynomial) to eliminate the exponential decay in the echo intensity, apodized with a Hamming window function, zero-filled to eight-fold points, and fast Fourier-transformed to yield the 2-dimensional frequency domain. For ^{13}C-minus-Natural Abundance (N.A). difference spectra, the time domain of the HYSCORE spectrum of the sample prepared using natural abundance CO was subtracted from that of the ^{13}CO sample, and the same data processing procedure detailed above was used to generate the frequency spectrum. Contour plots of the 2D frequency spectra are plotted in logarithmic scale, with contours plotted in colors ranging from blue → yellow → red in increasing intensity.

In general, the ENDOR spectrum for a given nucleus with spin $I = ½$ (^{1}H, ^{13}C, ^{31}P) coupled to the $S = ½$ electron spin exhibits a doublet at frequencies

$$\nu_{\pm} = \left|\frac{A}{2} \pm \nu_N\right| \tag{1}$$

Where ν_N is the nuclear Larmor frequency and A is the hyperfine coupling. For nuclei with $I \geq 1$ (^{14}N, ^{2}H), an additonal splitting of the $\nu_{\pm}$ manifolds is produced by the nuclear quadrupole interaction (P)

$$\nu_{\pm,m_I} = \left|\nu_N \pm \frac{3P(2m_I - 1)}{2}\right| \tag{2}$$

In HYSCORE spectra, these signals manifest as cross-peaks or ridges in the 2-D frequency spectrum which are generally symmetric about the diagonal of a given quadrant. This technique allows hyperfine levels corresponding to the same electron-nuclear submanifold to be differentiated, as well as separating features from hyperfine couplings in the weak-coupling regime ($|A| < 2|\nu_I|$) in the (+,+) quadrant from those in the strong coupling regime ($|A| > 2|\nu_I|$) in the (−,+) quadrant. The (−,−) and (+,−) quadrants of these frequency spectra are symmetric to the (+,+) and (−,+) quadrants, thus only two of the quadrants are typically displayed in literature.
For systems with appreciable hyperfine anisotropy in frozen solutions or solids, HYSCORE spectra typically do not exhibit sharp cross peaks, but show ridges that represent the sum of cross peaks from selected orientations within the excitation bandwidth of the MW pulses at the magnetic

field position at which the spectrum is collected. The length and curvature of these correlation ridges can allow for the separation and estimation of the magnitude of the isotropic and dipolar components of the hyperfine tensor, as shown in Fig. S23.

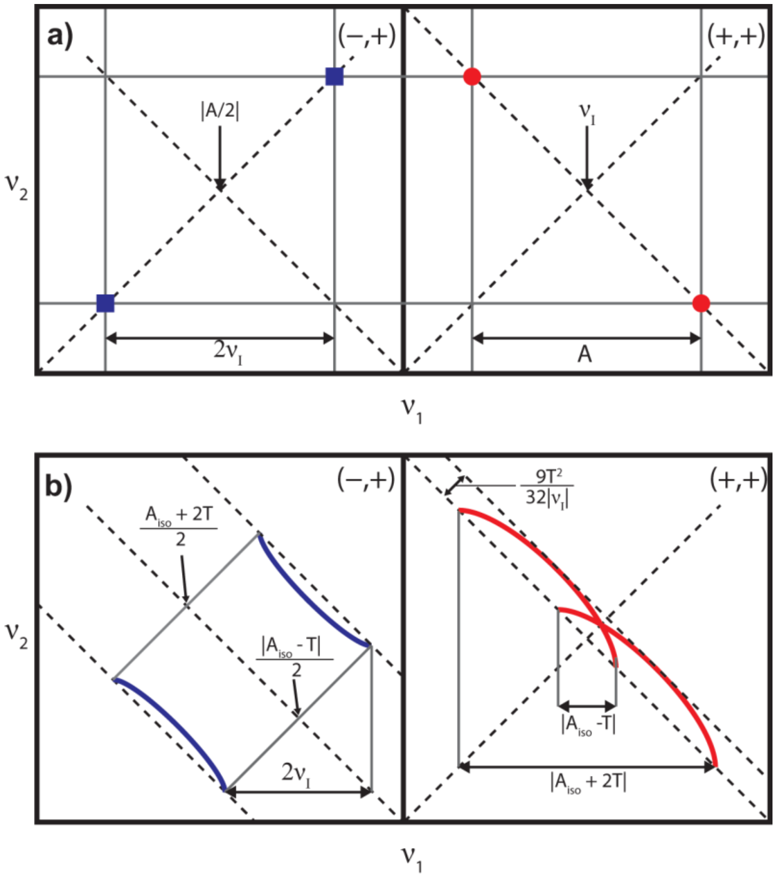

Figure S23—a) HYSCORE powder patterns for an S = ½, I = ½ spin system with an isotropic hyperfine tensor A. b) HYSCORE powder patterns for an S = ½, I = ½ spin system with an axial hyperfine tensor that contains isotropic (a_{iso}) and dipolar (T) contributions. Blue correlation ridges represent the strong coupling case; red correlation ridges represent the weak coupling case.

EPR Simulations. Simulations of all CW and pulse EPR data were achieved using the EasySpin simulation toolbox (release 5.2.25)[13] with Matlab 2019a using the following Hamiltonian:

$$\hat{H} = \mu_B \vec{B}_0 g \hat{S} + \mu_N g_N \vec{B}_0 \hat{I} + h\hat{S} \cdot \boldsymbol{A} \cdot \hat{I} + h\hat{I} \cdot \boldsymbol{P} \cdot \hat{I} \quad (3)$$

In this expression, the first term corresponds to the electron Zeeman interaction term where μ_B is the Bohr magneton, g is the electron spin g-value matrix with princial components g = [g_{xx} g_{yy} g_{zz}], and $\hat{S}$ is the electron spin operator; the second term corresponds to the nuclear Zeeman interaction term where μ_N is the nuclear magneton, g_N is the characteristic nuclear g-value for each nucleus (e.g. ^{1}H, ^{13}C) and $\hat{I}$ is the nuclear spin operator; the third term corresponds to the electron-nuclear hyperfine term, where $\boldsymbol{A}$ is the hyperfine coupling tensor with principal components $\boldsymbol{A}$ = [A_{xx}, A_{yy}, A_{zz}]; and for nuclei with $I \geq 1$, the final term corresponds to the nuclear quadrupole (NQI) term

which arises from the interaction of the nuclear quadrupole moment with the local electric field gradient (efg) at the nucleus, where $\boldsymbol{P}$ is the quadrupole coupling tensor. In the principal axis system (PAS), $\boldsymbol{P}$ is traceless and parametrized by the quadrupole coupling constant e^2Qq/h and the asymmetry parameter η such that:

$$\boldsymbol{P} = \begin{pmatrix} P_{xx} & 0 & 0 \\ 0 & P_{yy} & 0 \\ 0 & 0 & P_{zz} \end{pmatrix} = \frac{e^2Qq/h}{4I(2I-1)} \begin{pmatrix} -(1-\eta) & 0 & 0 \\ 0 & -(1+\eta) & 0 \\ 0 & 0 & 2 \end{pmatrix} \quad (4)$$

where $\frac{e^2Qq}{h} = 2I(2I-1)P_{zz}$ and $\eta = \frac{P_{xx}-P_{yy}}{P_{zz}}$. The asymmetry parameter may have values between 0 and 1, with 0 corresponding to an electric field gradient (EFG) with axial symmetry and 1 corresponding to a fully rhombic EFG.

The orientations between the hyperfine and NQI tensor principal axis systems and the g-matrix reference frame are defined by the Euler angles (α, β, γ), with rotations performed within the zyz convention where α rotates xyz counterclockwise about z-axis to give x'y'z', β rotates x'y'z' counterclockwise about y'-axis to give x",y",z", γ rotates xyz counterclockwise about z"-axis to give final frame orientation.

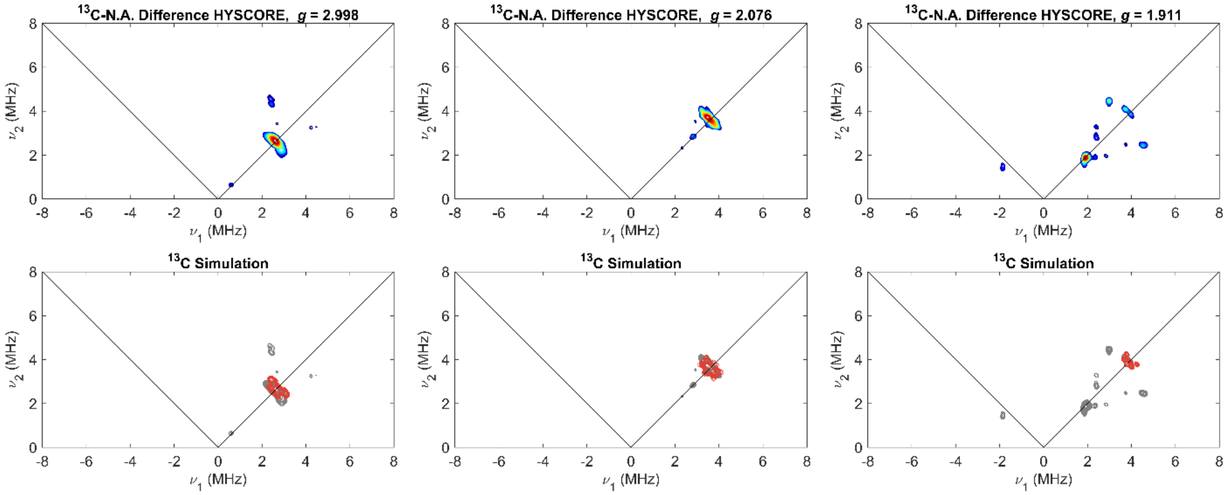

Figure S24. Top Panels: Field-dependent X-band ^{13}C-minus-Natural Abundance (N.A.) HYSCORE of **4-K**(^{13}CO). Bottom Panels: Experimental HYSCORE spectrum (gray contours) with overlay of simulated ^{13}C HYSCORE spectrum (A(^{13}C) = [-0.5, 1.0, -0.5] MHz) in red. Acquisition parameters: Temperature = 3.6 K; B_0 = 242 mT (g = 2.998), 335 mT (g = 2.076), 364 mT (g = 1.911); MW Frequency = 9.736 GHz; MW pulse lengths ($\pi/2, \pi$) = 8 ns, 16 ns; τ = 98 ns (g = 2.998), 140 ns (g = 2.076), 130 ns (g = 1.911); t_1 = t_2 = 100 ns; Δt_1 = Δt_2 =12 ns; shot repetition time (srt) = 1 ms.

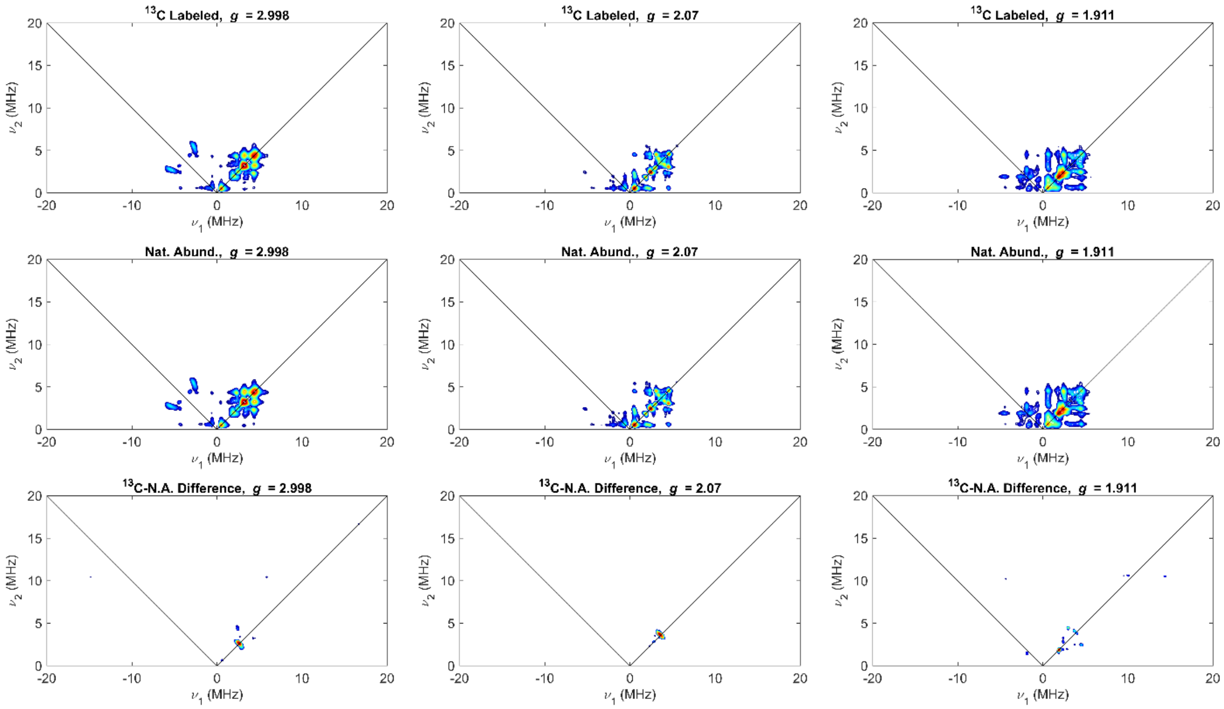

Fig S25. Top Panels: Field-dependent X-band HYSCORE of **4-K**(^{13}CO) (top panels)**, 4-K** (middle panels) , and the ^{13}C-N.A. difference spectra plotted in the bottom panels. Acquisition parameters: Temperature = 3.6 K; B_0 = 242 mT (g = 2.998), 335 mT (g = 2.076), 364 mT (g = 1.911); MW Frequency = 9.736 GHz; MW pulse lengths ($\pi/2, \pi$) = 8 ns, 16 ns; τ = 98 ns (g = 2.998), 140 ns (g = 2.076), 130 ns (g = 1.911); t_1 = t_2 = 100 ns; Δt_1 = Δt_2 =12 ns; shot repetition time (srt) = 1 ms.

B) Crystallographic information:

1. *X-ray crystallography:*

XRD data were collected at 100 K on a Bruker AXS D8 KAPPA or Bruker AXS D8 VENTURE diffractometer [microfocus sealed X-ray tube, λ(Mo Kα) = 0.71073 Å or λ(Cu Kα) = 1.54178 Å]. All manipulations, including data collection, integration, and scaling, were carried out using the Bruker *APEX3* software.[14] Absorption corrections were applied using *SADABS*.[15] Structures were solved by direct methods using *Sir92*[16] or *SUPERFLIP*[17] and refined using full-matrix least-squares on *CRYSTALS*[18] to convergence. All non-H atoms were refined using anisotropic displacement parameters. H atoms were placed in idealized positions and refined using a riding model. Because of the size of the compounds some crystals included solvent-accessible voids that contained disordered solvent. The solvent could be either modeled satisfactorily, or accounted for using either the *SQUEEZE* procedure in the *PLATON* software package.[19]

2. *Additional information:*

Special refinement details for **2-^{t}Bu**. The asymmetric unit of the structure contains 3 co-crystallized C_6H_6 solvent molecule, which can be modeled satisfactorily using bond lengths and similarity restraints for ADPs. The backbone of the five-membered chelate portion containing the two N^iPr_2 groups is disordered over 2 positions, with occupancies of 43% and 57%.

Special refinement details for **3**. The asymmetric unit of the structure contains two co-crystallized THF solvent molecule which can be modeled satisfactorily using bond lengths and similarity restraints for ADPs. Two N-^{i}Pr fragments on one BAC ligand are disordered over two positions, with occupancies of 38% and 62%, and 33% and 67%.

Special refinement details for **4**. The asymmetric unit of the structure contains half of a co-crystallized C_6H_6 and 2 Et_2O solvent molecules, which can be modeled satisfactorily using bond lengths and similarity restraints for ADPs.

Special refinement details for **4-K**. The asymmetric unit of the structure contains two co-crystallized Et_2O solvent molecules, which can be modeled satisfactorily using bond lengths and similarity restraints for ADPs. The K(THF) fragments are disordered over two positions, with occupancies of 36% and 64%. In one position, the positions of the atoms within the THF molecule tend to oscillate, so a shift-limiting restrain was applied to stabilize them.

Special refinement details for **4-K(18-crown-6)**. The asymmetric unit of the structure contains heavily disordered solvent molecules and cannot be modeled satisfactorily. Therefore, the electron density for co-crystallized solvent molecules were accounted for using the *SQUEEZE* procedure in *PLATON*[19], whereby 37 electrons were found in a volume of 235 $Å^3$, consistent with the presence of 0.5[$C_4H_{10}O$] in the asymmetric unit.

Special refinement details for **5**. The asymmetric unit of the structure contains two co-crystallized THF solvent molecules, which can be modeled satisfactorily using bond lengths and similarity restraints for ADPs.
Special refinement details for **6**. The asymmetric unit of the structure contains one co-crystallized Et_2O solvent molecule, which can be modeled satisfactorily using bond lengths and similarity

restraints for anisotropic displacement parameters (ADPs). The remaining solvent molecules are heavily disordered and cannot be modeled satisfactorily. Therefore, the electron density for co-crystallized solvent molecules were accounted for using the *SQUEEZE* procedure in *PLATON*[19], whereby 42 electrons were found in a volume of 474 Å^3, consistent with the presence of 0.5[$C_4H_{10}O$] in the asymmetric unit.

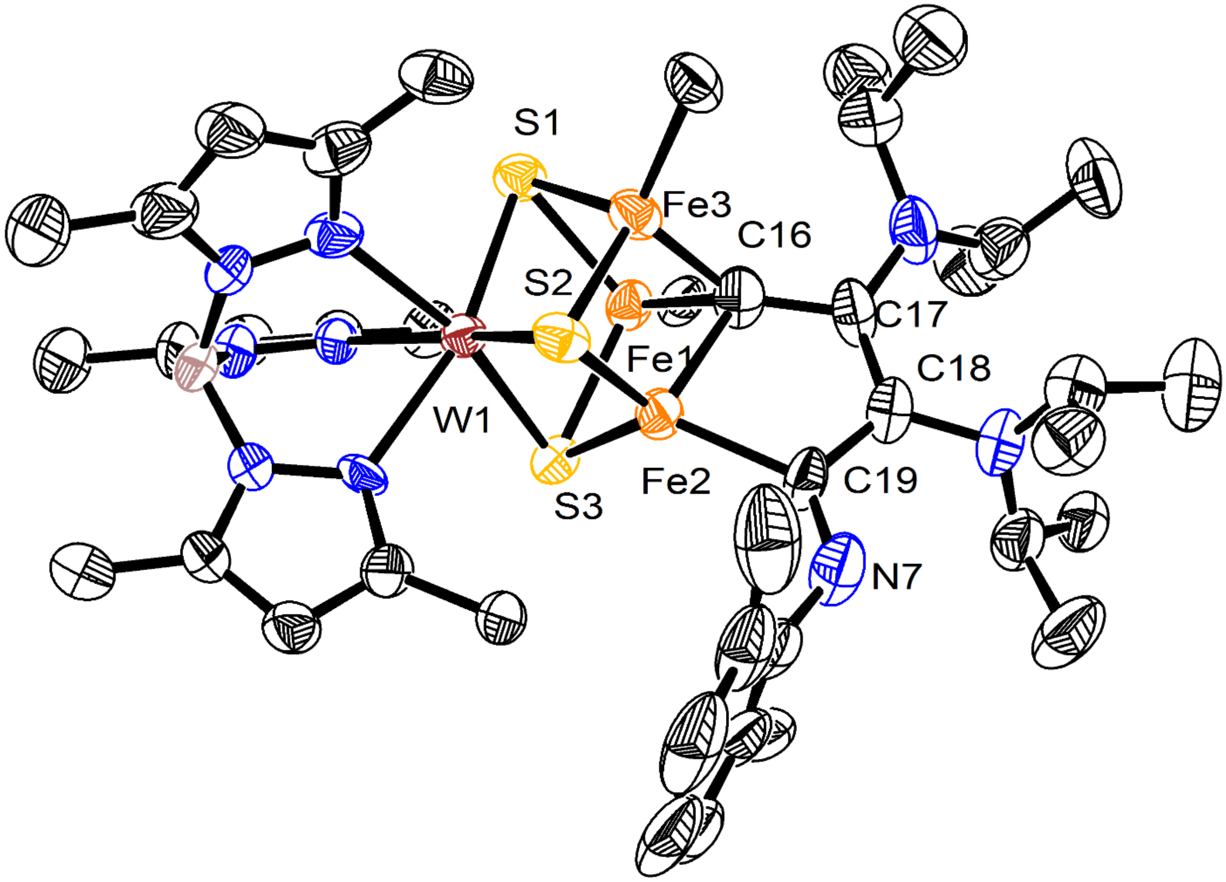

Figure S26: Crystal structure of **2-Xyl**. Ellipsoids are shown at 50% probability level. Hydrogen atoms, solvent molecules, and the BAC ligand except for the carbene C are omitted for clarity.

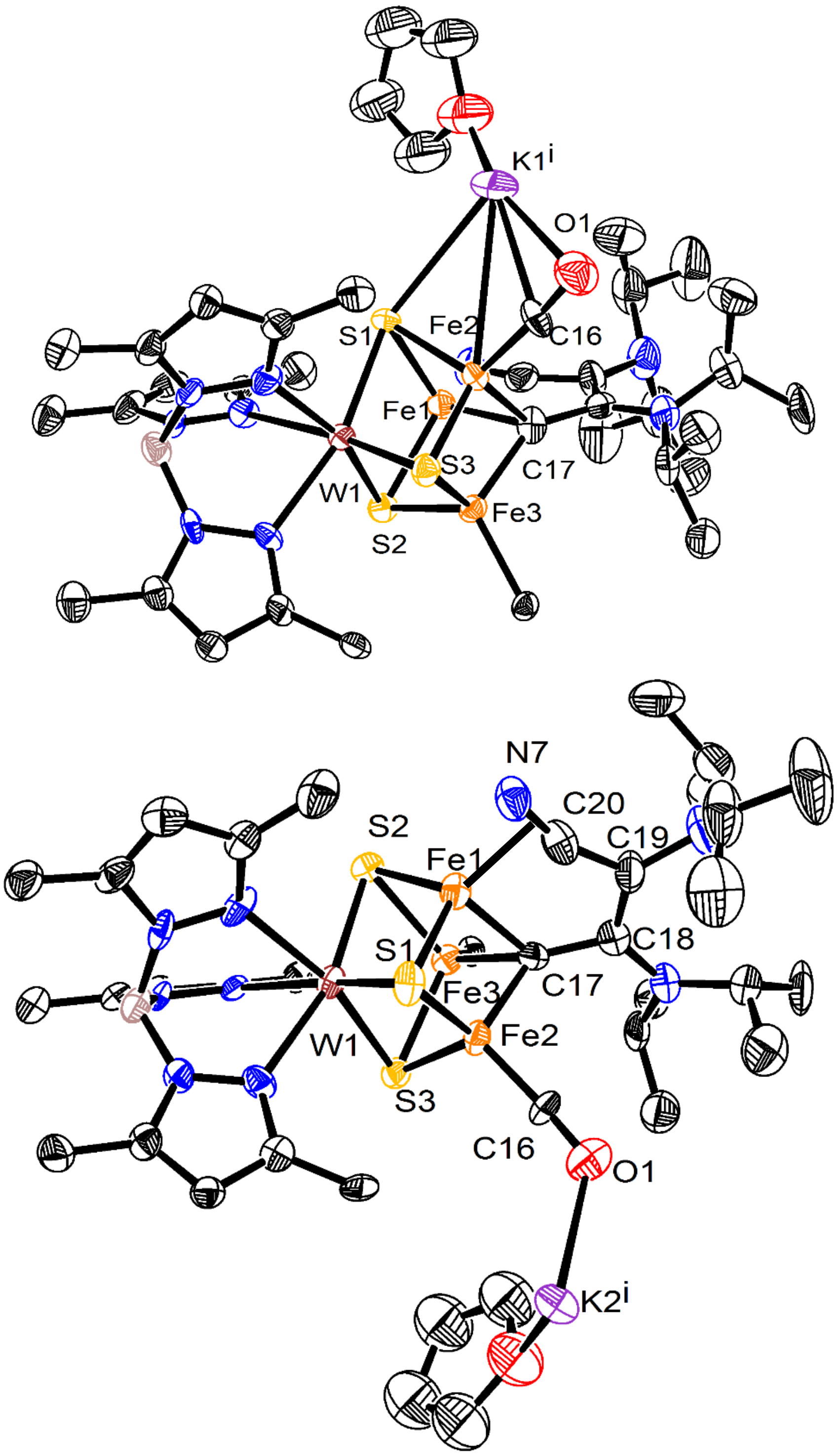

Figure S27: Crystal structure of **4-K** in two views showing the two disordered positions of the K atom. Ellipsoids are shown at 50% probability level. Hydrogen atoms, solvent molecules, and the BAC ligand except for the carbene C are omitted for clarity.

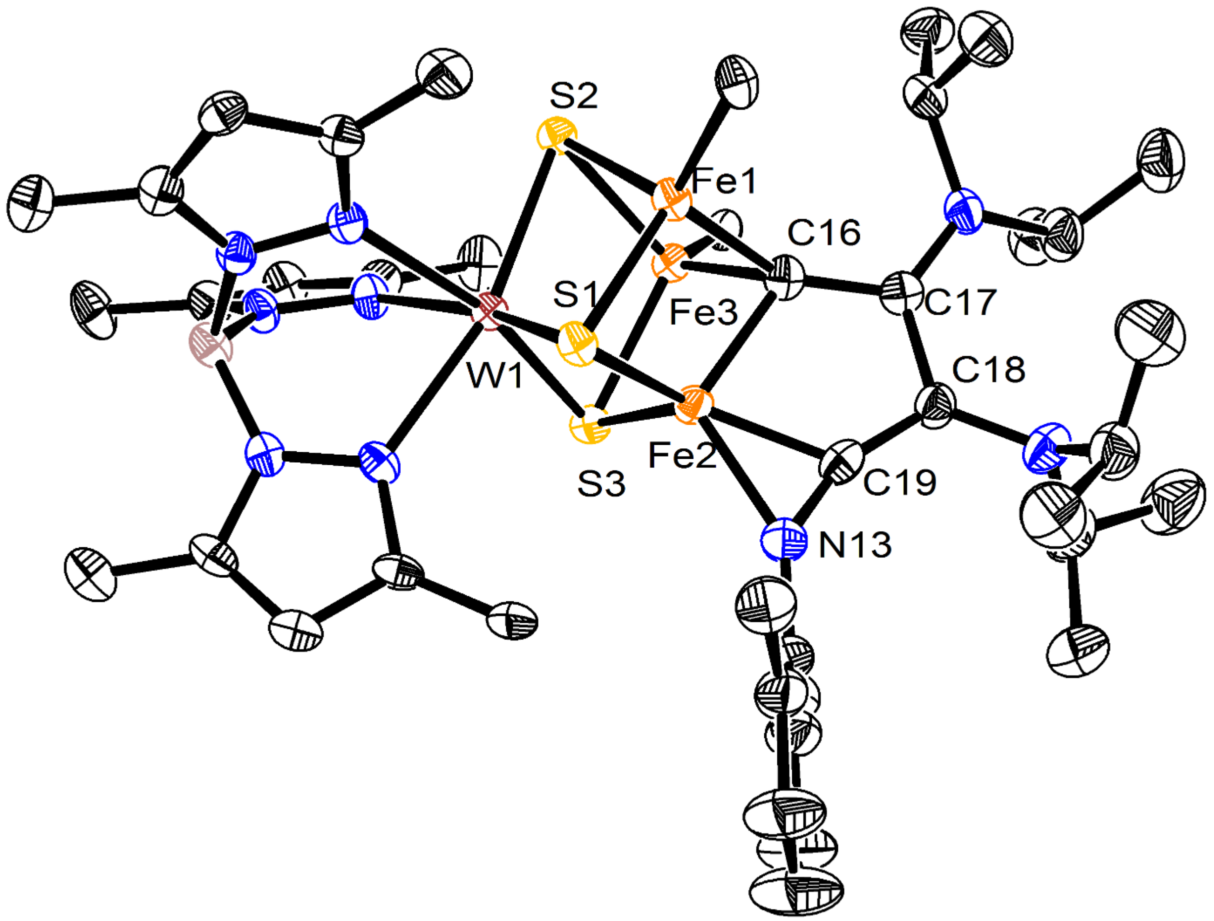

Figure S28: Crystal structure of **5**. Ellipsoids are shown at 50% probability level. Hydrogen atoms, counterions, solvent molecules, and the BAC ligand except for the carbene C are omitted for clarity.

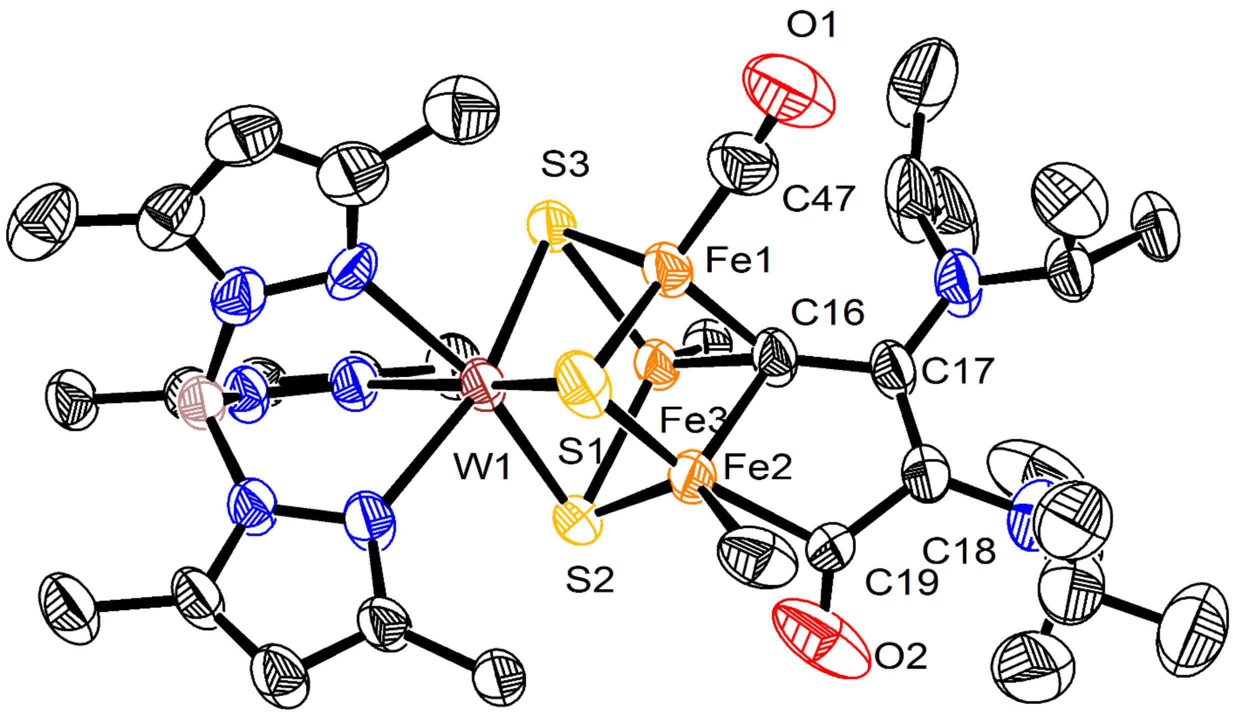

Figure S29: Crystal structure of **6**. Ellipsoids are shown at 50% probability level. Hydrogen atoms, solvent molecules, and the BAC ligand except for the carbene C are omitted for clarity.

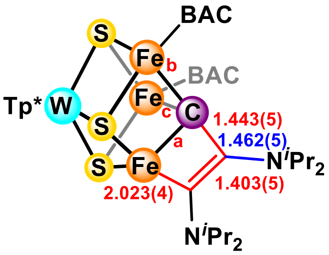

1

a = 1.943(3)
b = 1.958(4)
c = 1.945(3)

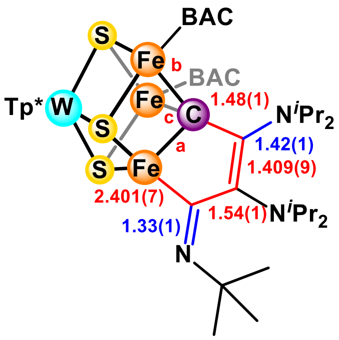

2-^{t}Bu

a = 1.919(6)
b = 1.944(8)
c = 1.953(7)

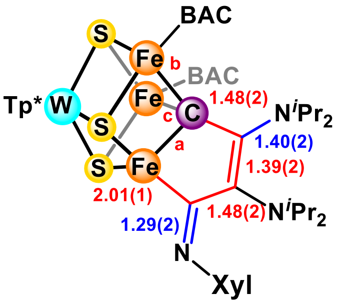

2-Xyl

a = 1.91(1)
b = 1.92(1)
c = 1.93(1)

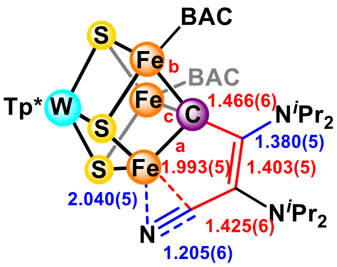

3

a = 1.988(4)
b = 1.939(4)
c = 1.930(4)

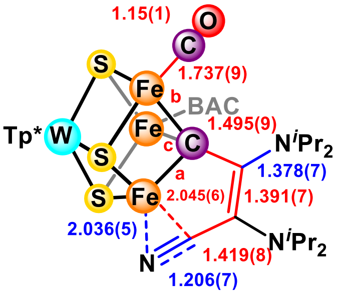

4

a = 2.009(6)
b = 1.880(8)
c = 1.953(8)

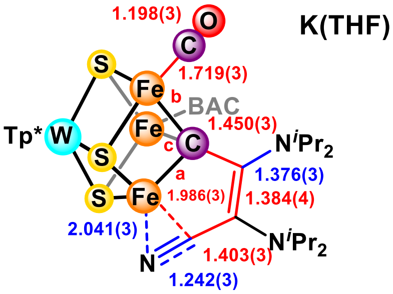

4-K

a = 2.046(3)
b = 1.908(2)
c = 1.955(4)

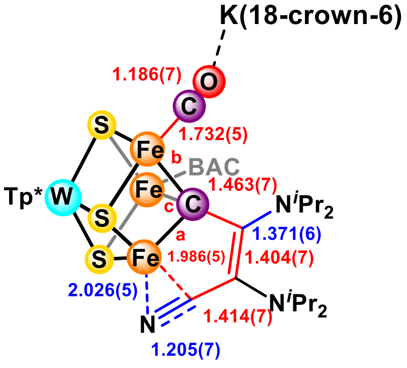

4-K(18-crown-6)

a = 2.021(5)
b = 1.900(7)
c = 1.961(4)

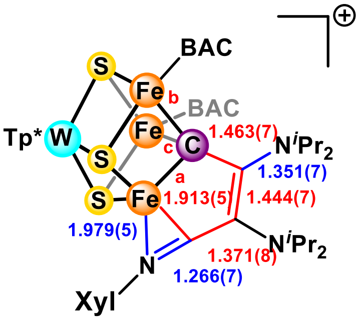

5

a = 1.945(5)
b = 1.961(5)
c = 1.948(5)

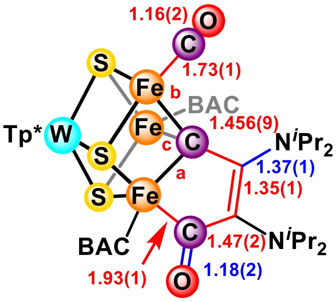

6

a = 1.943(7)
b = 1.956(8)
c = 1.907(8)

Figure S30: Bond length comparisons in Å for the clusters reported for selected bonds. The abbreviations a, b, and c refer to the three Fe-C(μ_3) distances as labeled in the structures.

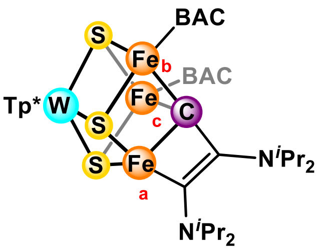

1

W-Fe(a) = 2.7203(5)	av. W-Fe = 2.7075
W-Fe(b) = 2.7089(5)	
W-Fe(c) = 2.6934(5)	
Fe(a)-Fe(b) = 2.5122(6)	
Fe(b)-Fe(c) = 2.5186(7)	av. Fe-Fe = 2.5054
Fe(c)-Fe(a) = 2.4854(7)	

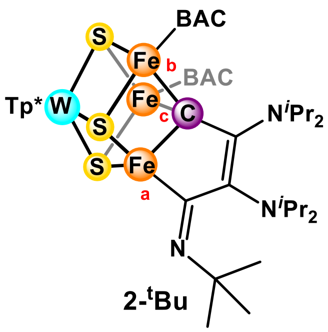

2-^{t}Bu

W-Fe(a) = 2.713(3)	av. W-Fe = 2.738
W-Fe(b) = 2.720(3)	
W-Fe(c) = 2.781(2)	
Fe(a)-Fe(b) = 2.514(2)	
Fe(b)-Fe(c) = 2.535(4)	av. Fe-Fe = 2.525
Fe(c)-Fe(a) = 2.526(3)	

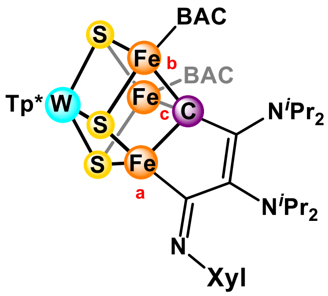

2-Xyl

W-Fe(a) = 2.687(2)	av. W-Fe = 2.700
W-Fe(b) = 2.708(2)	
W-Fe(c) = 2.705(2)	
Fe(a)-Fe(b) = 2.519(2)	
Fe(b)-Fe(c) = 2.528(3)	av. Fe-Fe = 2.511
Fe(c)-Fe(a) = 2.486(2)	

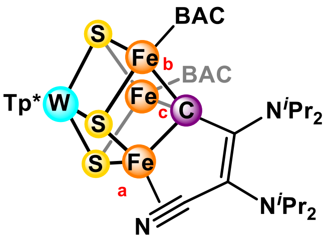

3

W-Fe(a) = 2.6790(9)	av. W-Fe = 2.705
W-Fe(b) = 2.7200(9)	
W-Fe(c) = 2.715(1)	
Fe(a)-Fe(b) = 2.5708(9)	
Fe(b)-Fe(c) = 2.5373(9)	av. Fe-Fe = 2.550
Fe(c)-Fe(a) = 2.541(1)	

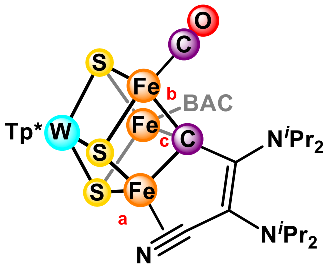

4

W-Fe(a) = 2.6786(9)	av. W-Fe = 2.688
W-Fe(b) = 2.657(1)	
W-Fe(c) = 2.727(1)	
Fe(a)-Fe(b) = 2.512(1)	
Fe(b)-Fe(c) = 2.501(2)	av. Fe-Fe = 2.526
Fe(c)-Fe(a) = 2.566(1)	

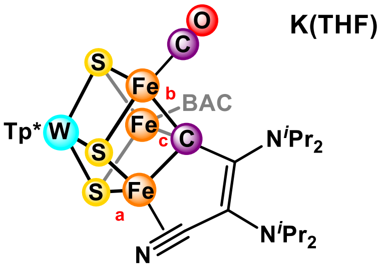

4-K

W-Fe(a) = 2.698(2)	av. W-Fe = 2.715
W-Fe(b) = 2.711(1)	
W-Fe(c) = 2.735(2)	
Fe(a)-Fe(b) = 2.549(2)	
Fe(b)-Fe(c) = 2.530(2)	av. Fe-Fe = 2.555
Fe(c)-Fe(a) = 2.587(3)	

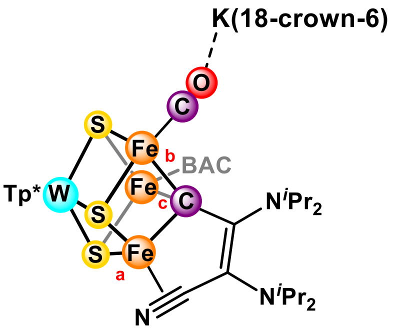

4-K(18-crown-6)

W-Fe(a) = 2.658(1)	av. W-Fe = 2.701
W-Fe(b) = 2.715(1)	
W-Fe(c) = 2.730(1)	
Fe(a)-Fe(b) = 2.541(1)	
Fe(b)-Fe(c) = 2.519(2)	av. Fe-Fe = 2.548
Fe(c)-Fe(a) = 2.585(1)	

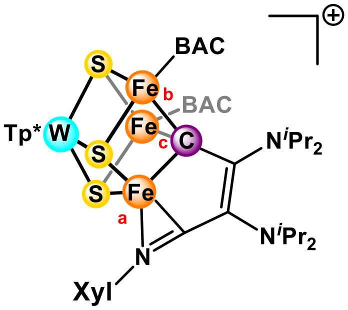

5

W-Fe(a) = 2.7263(9)	av. W-Fe = 2.7309
W-Fe(b) = 2.7421(9)	
W-Fe(c) = 2.7243(8)	
Fe(a)-Fe(b) = 2.520(1)	
Fe(b)-Fe(c) = 2.554(1)	av. Fe-Fe = 2.540
Fe(c)-Fe(a) = 2.546(1)	

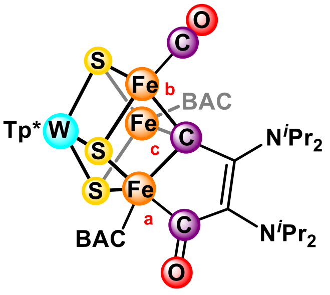

6

W-Fe(a) = 2.811(1)	av. W-Fe = 2.736
W-Fe(b) = 2.692(1)	
W-Fe(c) = 2.706(1)	
Fe(a)-Fe(b) = 2.498(2)	
Fe(b)-Fe(c) = 2.500(2)	av. Fe-Fe = 2.523
Fe(c)-Fe(a) = 2.571(2)	

Figure S31: Metal-metal distances in Å for the clusters reported.

Table S4: Summary of statistics for diffraction data for clusters **2** – **4**

**Cluster**	**2-^{t}Bu**	**2-Xyl**	**3**	**4**
**CCDC**	2130433	2233067	2233068	2130436
**Empirical formula**	$C_{83}H_{133}BFe_3N_{13}S_3W$	$C_{73}H_{125}BFe_3N_{13}OS_3W$	$C_{69}H_{122}BFe_3N_{13}O_2S_3W$	$C_{58}H_{101}BFe_3N_{11}O_3S_3W$
**Formula weight**	1771.45	1659.28	1624.21	1458.91
**Temperature/K**	100	100	100	100
**Crystal system**	Monoclinic	Triclinic	Monoclinic	Monoclinic
**Space group**	Cc	P-1	$P2_1/n$	C2/c
**a/Å**	23.8881(12)	14.394(2)	20.374(7)	40.500(3)
**b/Å**	15.1745(7)	16.501(2)	17.922(3)	16.2716(10)
**c/Å**	26.2682(14)	17.357(4)	21.852(6)	24.9399(16)
**α/°**	90	76.625(13)	90	90
**β/°**	111.144(5)	81.965(6)	101.54(2)	125.5559(16)
**γ/°**	90	87.939(10)	90	90
**Volume/Å^3**	8880.9(8)	3971.5(12)	7818(4)	13370.9(15)
**Z**	4	2	4	8
**ρ_{calc}/g cm^{-3}**	1.325	1.387	1.380	1.449
**μ/mm^{-1}**	7.209	8.030	8.155	2.496
**F(000)**	3708.0	1734.0	14202.0	6040.0
**Crystal size/mm^3**	0.05 × 0.06 × 0.10	0.01 × 0.05 × 0.20	0.02 × 0.08 × 0.16	0.05 × 0.05 × 0.10
**Radiation**	Cu Kα	Cu Kα	Cu Kα	Mo Kα
**θ_{max}/°**	80.685	74.820	74.820	33.976
**Index ranges**	$-30 \leq h \leq 27, -19 \leq k \leq 19, -32 \leq l \leq 32$	$-15 \leq h \leq 17, -20 \leq k \leq 20, -21 \leq l \leq 21$	$-25 \leq h \leq 25, -22 \leq k \leq 22, -27 \leq l \leq 27$	$-55 \leq h \leq 63, -25 \leq k \leq 25, -39 \leq l \leq 39$
**Reflections measured**	91969	91921	139311	136877
**Independent reflections**	17918	16152	15954	27087
**Restraints/Parameters**	324/1082	0/856	234/903	104/726
**GOF on F^2**	0.992	0.969	1.006	1.003
**R-factor**	0.0564	0.0941	0.0410	0.0783
**Weighted R-factor**	0.1463	0.2556	0.1078	0.1939
**Largest diff. peak/hole/e Å^{-3}**	0.79/-1.24	2.90/-4.85	1.79/-1.11	4.75/-6.37

Table S5: Summary of statistics for diffraction data for clusters **4-K** – **6**

Cluster	4-K	4-K(18-crown-6)	5	6
**CCDC**	2233070	2233072	2233069	2130434
**Empirical formula**	$C_{59}H_{106}BFe_3KN_{11}O_4S_3W$	$C_{59}H_{102}BFe_3KN_{11}O_7S_3W$	$C_{78}H_{131}BF_3Fe_3N_{13}O_5S_4W$	$C_{66}H_{116}BFe_3N_{12}O_3S_3W$
**Formula weight**	1531.06	1575.03	1878.44	1584.12
**Temperature/K**	100	100	100	100
**Crystal system**	Monoclinic	Triclinic	Monoclinic	Triclinic
**Space group**	$P2_1/c$	P-1	$P2_1/c$	P-1
**a/Å**	16.2062(5)	13.942(4)	15.131(3)	13.4439(6)
**b/Å**	31.3345(9)	16.201(5)	25.397(3)	16.4143(6)
**c/Å**	14.0576(5)	17.697(7)	23.235(6)	19.0773(7)
**α/°**	90	73.25(2)	90	97.603(2)
**β/°**	93.6639(18)	69.84(2)	95.143(12)	103.792(2)
**γ/°**	90	79.89(3)	90	97.819(3)
**Volume/Å^3**	7124.0(4)	3580(2)	8893(3)	3990.6(3)
**Z**	4	2	4	2
**ρ_{calc}/g cm^{-3}**	1.427	1.461	1.403	1.318
**μ/mm^{-1}**	9.441	9.444	7.538	7.981
**F(000)**	3172.0	1626.0	3912.0	1650.0
**Crystal size/mm^3**	0.02 × 0.09 × 0.12	0.02 × 0.05 × 0.06	0.08 × 0.12 × 0.20	0.05 × 0.10 × 0.15
**Radiation**	Cu Kα	Cu Kα	Cu Kα	Cu Kα
**θ_{max}/°**	74.616	76.224	74.636	80.292
**Index ranges**	$-20 \leq h \leq 19$, $-39 \leq k \leq 38$, $-17 \leq l \leq 17$	$-16 \leq h \leq 17$, $-19 \leq k \leq 20$, $-22 \leq l \leq 22$	$-18 \leq h \leq 18$, $-31 \leq k \leq 30$, $-28 \leq l \leq 29$	$-17 \leq h \leq 16$, $-20 \leq k \leq 20$, $-24 \leq l \leq 24$
**Reflections measured**	99392	93037	131294	76077
**Independent reflections**	14543	14706	18124	17199
**Restraints/Parameters**	758/803	0/775	65/973	64/802
**GOF on F^2**	1.054	0.971	1.025	0.978
**R-factor**	0.1067	0.0508	0.0559	0.0769
**Weighted R-factor**	0.2314	0.1513	0.1252	0.2179
**Largest diff. peak/hole/e Å^{-3}**	5.47/-3.19	5.02/-2.54	3.47/-1.98	4.25/-2.23

C) Computational details:

Computational procedure:

The r^2SCAN[20] density functional was used that has been benchmarked[21] with respect to the structural properties of analogous Fe-S containing systems. The r^2SCAN functional does not include Hartree-Fock exchange so that it is less costly and facilitates the characterization of large chemical systems. The defgrid2 integration grid in ORCA was used for the geometry optimizations. The D4 empirical dispersion developed by Grimme[22] with respect to parameters reported by Brandenburg.[23] The relativistically contracted ZORA-def2-TZVP[24,25] basis set was used for all eligible elements. The all-electron SARC-ZORA-TZVP[26] basis was used for W. The CPCM[27] solvation model was used with respect to the dielectric constant and refractive index of THF.

The structures were optimized with respect to the broken symmetry solution of the stated multiplicity. The six distinct broken symmetry solutions that can result from the magnetic coupling between four open-shell centers were considered with the SpinFlip module in ORCA. We found that all calculations converged to an identical wavefunction.

The local spin states and pairwise electronic interactions of the metal centers were assigned with interpretation of their Pipek-Mezey (PM) localization method.[28]

The CO vibrational frequencies were calculated from the partial Hessian of the CO and coordinating Fe center.

The Mössbauer isomer shift (δ) were calculated from the electron densities of the Fe nuclei (ρ_0) that have a linear relationship with respect to the empirical parameters α, β, and C.[29,30] The parameters for their linear relationship were calibrated with respect to Fe-carbonyl compounds whose experimental Mössbauer properties[31] are provided in Table S11. The optimized geometries were obtained from the same computational procedure and their coordinates are provided. The quadrupole splitting (ΔE_Q) was calculated separately with a CP(PPP) basis set[32] applied to the Fe-centers. The defgrid3 integration grid was used for calculating the Mössbauer parameters.

All calculations were done with the ORCA v5.03 quantum chemistry code.[33]

Geometry optimizations:

Table S6: The comparison of the experimental metal-metal bond lengths of **4-K(18-crown-6)** and the optimized quartet state its charged, **4**$^{-}$, and neutral, **4-K**, states. The RMSD is provided separately for the W-Fe and Fe-Fe bonds.

**Bond**	**4-K(18-crown-6) (Å)**	**4^{-} (Å)**	**4-K (Å)**
W-Fe(CO)	2.72	2.63	2.64
W-Fe(BAC)	2.73	2.66	2.67
W-Fe(CN)	2.66	2.62	2.62
Fe(CO)-Fe(BAC)	2.52	2.52	2.52
Fe(CO)-Fe(CN)	2.54	2.53	2.52
Fe(BAC)-Fe(CN)	2.59	2.57	2.57
RMSD(W-Fe)		0.07	0.07
RMSD(Fe-Fe)		0.01	0.02

Table S7: The comparison of the experimental metal-metal bond lengths of **4** and the optimized quintet and triplet state. The RMSD is provided separately for the W-Fe and Fe-Fe bonds.

**Bond**	**4 (Å)**	**M_s = 2 (Å)**	**M_s = 1 (Å)**
W-Fe(CO)	2.66	2.64	2.64
W-Fe(BAC)	2.73	2.67	2.64
W-Fe(CN)	2.68	2.61	2.61
Fe(CO)-Fe(BAC)	2.51	2.46	2.51
Fe(CO)-Fe(CN)	2.51	2.51	2.52
Fe(BAC)-Fe(CN)	2.57	2.53	2.45
RMSD(W-Fe)		0.05	0.06
RMSD(Fe-Fe)		0.03	0.07

CO vibrational mode:

Table S8: The experimental and calculated CO vibrational mode of **4**, **4**$^-$, and **4-K**. We consider both the K^+ and K(18-crown-6)$^+$ salts and the bare anion for **4**$^-$. The calculated vibrational mode is reported for both the quintet and triplet state for **4**. We do not apply a scaling factor to the calculated vibrational modes.

Cluster	Experimental ν_{CO} (cm^{-1})	Calculated ν_{CO} (cm^{-1})
**4-K(18-crown-6)**	1782	
**4-K**	1794<br>1751	1756
**4**$^-$		1802
**4**	1851	1880 (M_s = 2)<br>1866 (M_s = 1)

Mössbauer parameters:

Table S9. The experimental and calculated Mössbauer isomer shifts (δ) for **4**$^-$ and **4-K**. The experimental values are reported with respect to the K^+-salt at 80 K. The parameters for calculating the isomer shifts are collected in Figure S29.

Site	Experimental<br>4-K<br>δ (mm s^{-1})	Calculated<br>4$^-$<br>δ (mm s^{-1})	Calculated<br>4-K<br>δ(mm s^{-1})
**Fe(CO)**	0.17	0.12	0.12
**Fe(BAC)**	0.35	0.33	0.33
**Fe(CN)**	0.47	0.49	0.48
**Avg.**	0.33	0.32	0.31

Table S10. The experimental and calculated Mössbauer isomer shifts (δ) for **4**. The values are presented for both the quintet and triplet state. The parameters for calculating the isomer shifts are collected in Figure S29.

Site	Experimental 4 δ (mm s^{-1})	Calculated 4 (M_s = 2) δ (mm s^{-1})	Calculated 4 (M_s = 1) δ (mm s^{-1})
**Fe(CO)**	0.02	0.11	0.15
**Fe(BAC)**	0.33	0.39	0.26
**Fe(CN)**	0.65	0.56	0.40
**Avg.**	0.33	0.35	0.27

Table S11. The experimental and calculated absolute Mössbauer quadrupole splitting ($|\Delta E_Q|$) for $\mathbf{4}^-$. The experimental values are reported with respect to the K^+-salt at 80 K.

Site	Experimental 4-K $|\Delta E_Q|$ (mm s^{-1})	Calculated 4^- $|\Delta E_Q|$ (mm s^{-1})	Calculated 4-K $|\Delta E_Q|$ (mm s^{-1})
**Fe(CO)**	1.84	1.62	1.38
**Fe(BAC)**	1.47	1.57	1.61
**Fe(CN)**	0.87	0.60	0.61

Table S12. The experimental and calculated absolute Mössbauer quadrupole splitting ($|\Delta E_Q|$) for **4**. The values are presented for both the quintet and triplet state.

Site	Experimental 4 $|\Delta E_Q|$ (mm s^{-1})	Calculated 4 (M_s = 2) $|\Delta E_Q|$ (mm s^{-1})	Calculated 4 (M_s = 1) $|\Delta E_Q|$ (mm s^{-1})
**Fe(CO)**	1.57	1.63	2.69
**Fe(BAC)**	1.19	0.90	1.54
**Fe(CN)**	1.07	0.65	0.99

Table S13: The experimental Mössbauer parameters (δ and $|\Delta E_Q|$) for the Fe-carbonyl compounds[31] that set the empirical parameters for the linear relationship between the calculated Fe-nuclear electron densities and isomer shift.

**Compound**	**δ (mm s^{-1})**	**$|\Delta E_Q|$ (mm s^{-1})**
$Fe(CO)_5$	-0.09	2.57
$Fe_2(CO)_9$	0.16	0.42
$Fe_3(CO)_{12}$	0.11 (66 %)<br>0.05 (33 %)	1.13 (66 %)<br>0.13 (33 %)
$[Fe(CO)_4]^{2-}$	-0.18	0
$[Fe_2(CO)_8]^{2-}$	-0.08	2.22
$[Fe_4(CO)_{13}]^{2-}$	0.02	0.27
$[Fe(CO)_4H]^{2-}$	-0.17	1.36
$[Fe_2(CO)_8H]^{2-}$	0.07	0.50
$[Fe_3(CO)_{11}H]^{2-}$	0.04 (66 %)<br>0.02 (33 %)	1.41 (66 %)<br>0.16 (33 %)
$Fe(Cp)(CO)_2I$	0.23	1.83

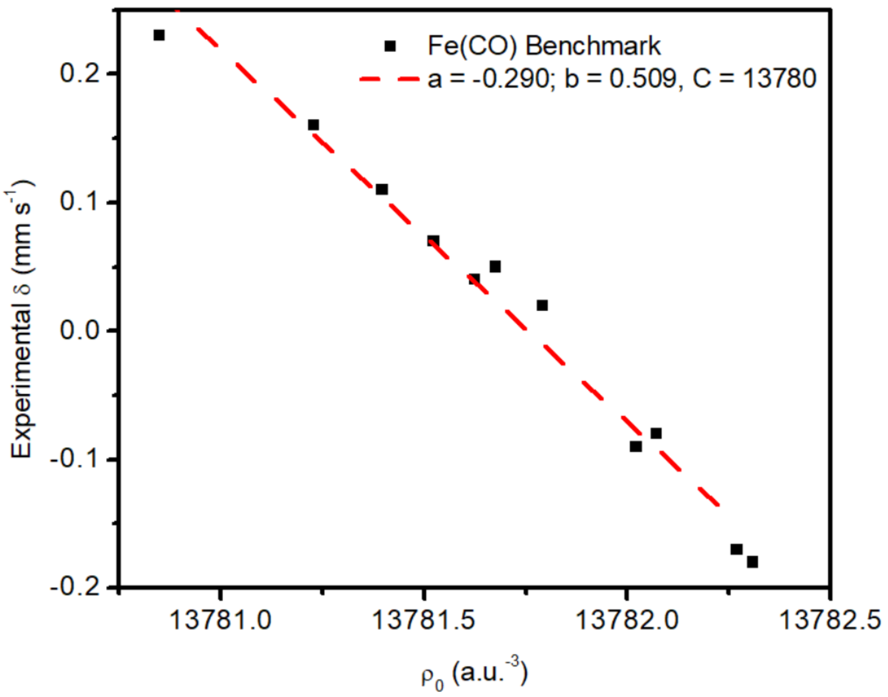

Figure S32. The linear relationship ($R^2 = 0.975$) between the calculated Fe-nuclear electron densities (ρ_0) and the experimental isomer shift (δ). The empirical parameters 'a', 'b', and 'C' are presented with respect to the detailed computational procedure.

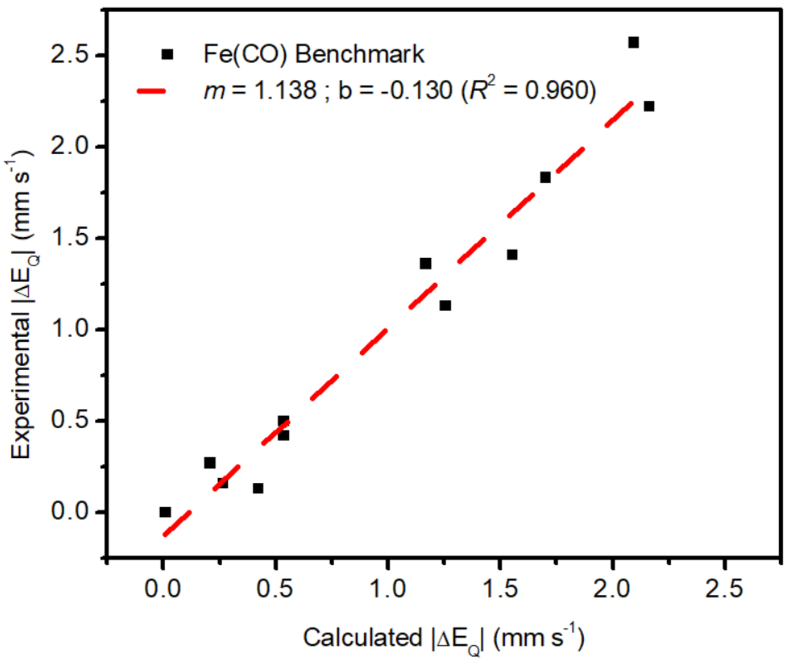

Figure S33. The linear relationship ($R^2 = 0.975$) between the experimental and calculated absolute quadrupole splitting ($|\Delta E_Q|$).

Localized orbital analysis of 4^-:

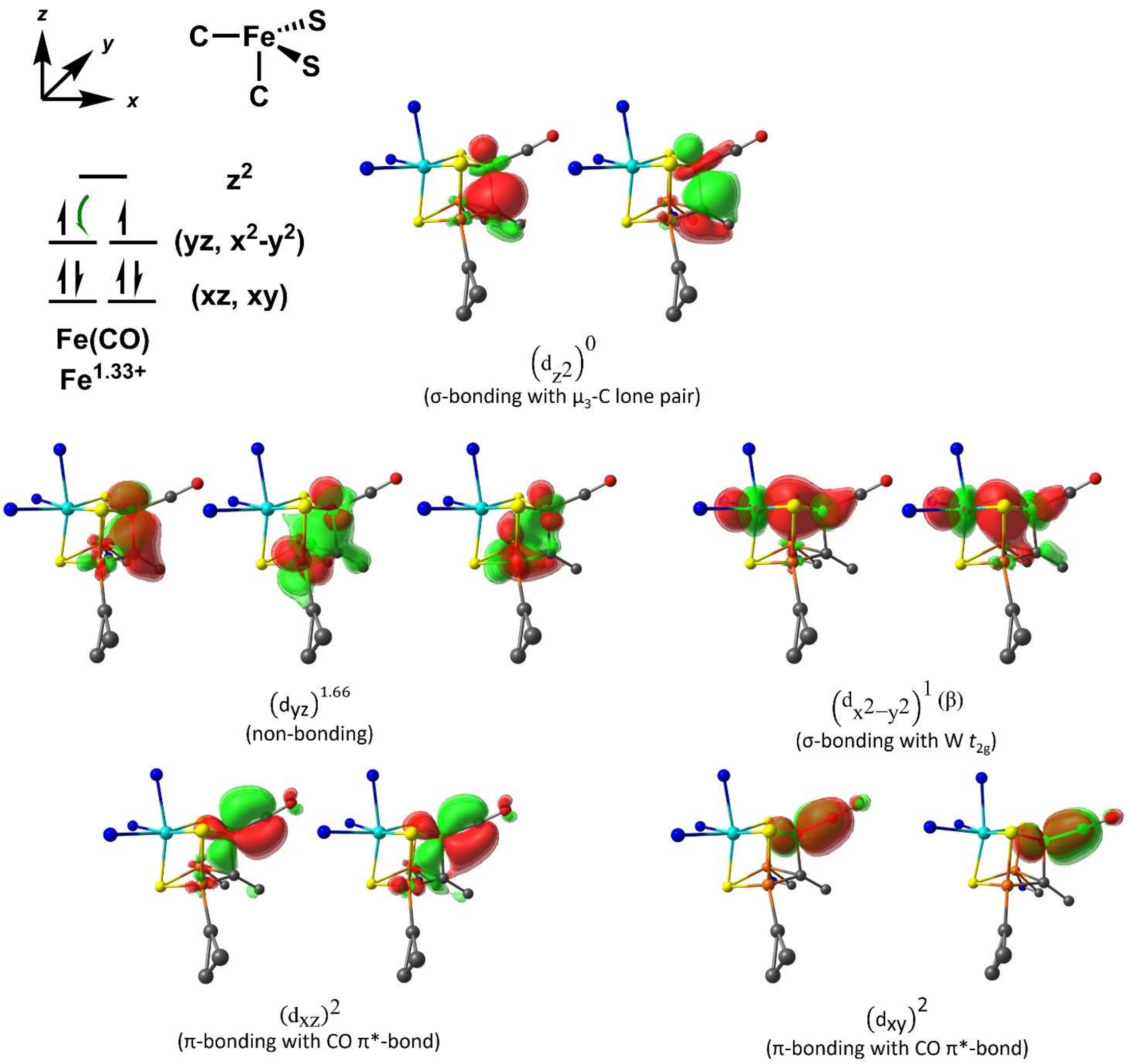

Figure S34. The PM localized orbitals for the Fe(CO) center in $\mathbf{4}^-$. The *z*-axis is oriented parallel to the Fe-carbyne bond. The electronic populations are specific to their α- or β-spin. The overall electronic configuration is included where the green arrow assigns 2/3 of an electron from delocalization.

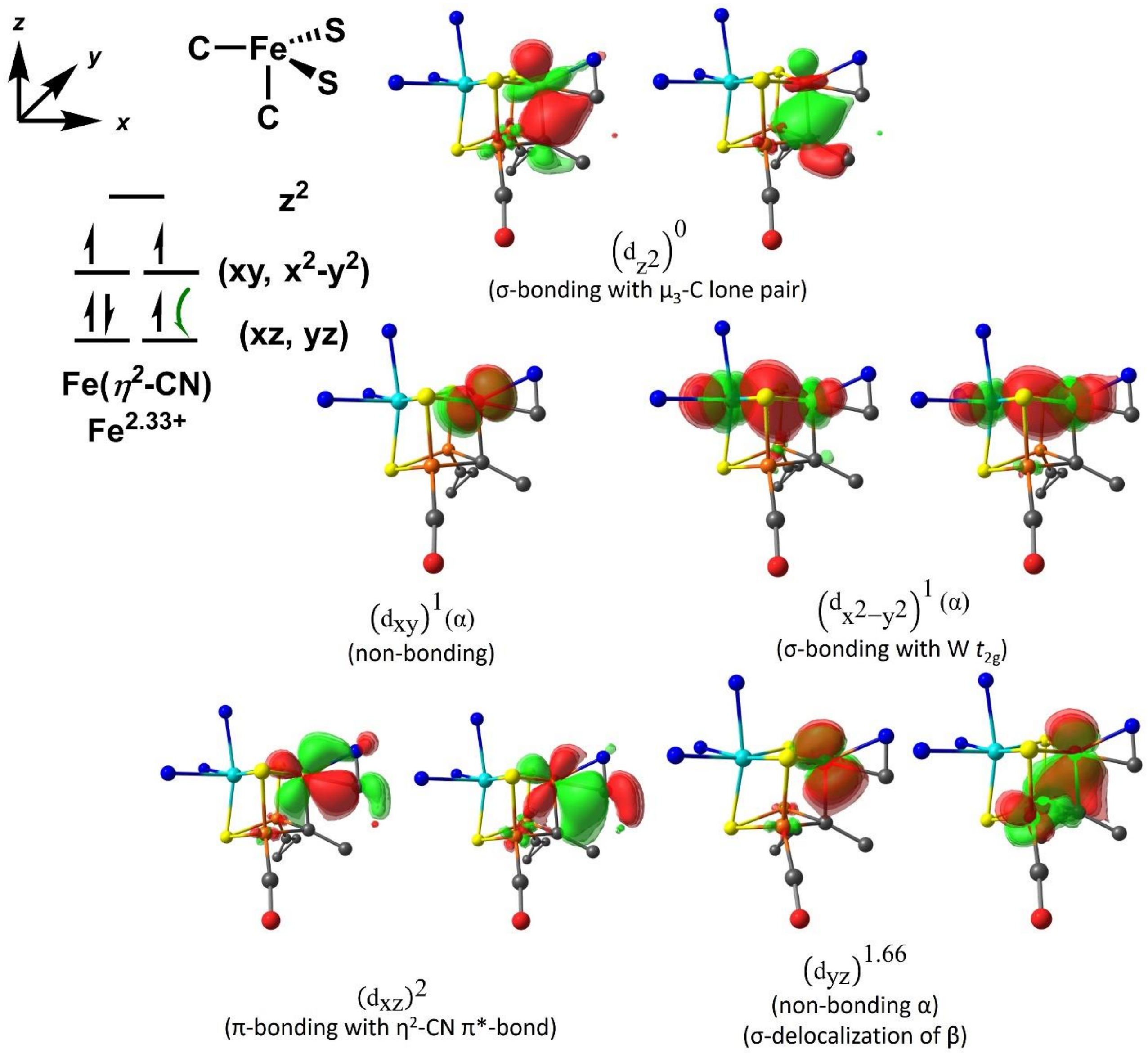

Figure S35. The PM localized orbitals for the Fe(η^2-CN) center in **4**$^-$. The *z*-axis is oriented parallel to the Fe-carbyne bond. The electronic populations are specific to their α- or β-spin. The overall electronic configuration is included where the green arrow assigns 2/3 of an electron from delocalization.

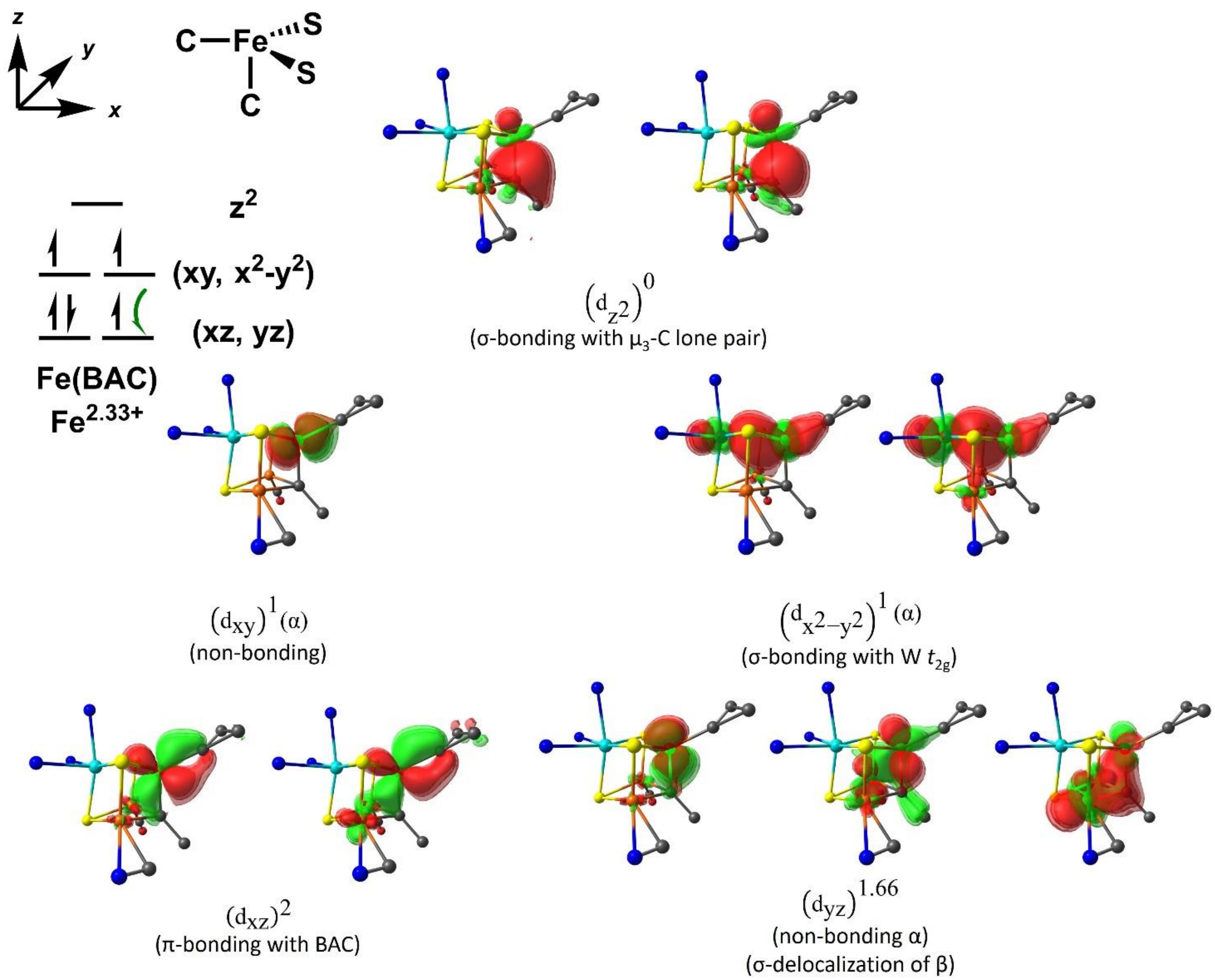

Figure S36. The PM localized orbitals for the Fe(BAC) center in **4**$^{-}$. The *z*-axis is oriented parallel to the Fe-carbyne bond. The electronic populations are specific to their α- or β-spin. The overall electronic configuration is included where the green arrow assigns 2/3 of an electron from delocalization.

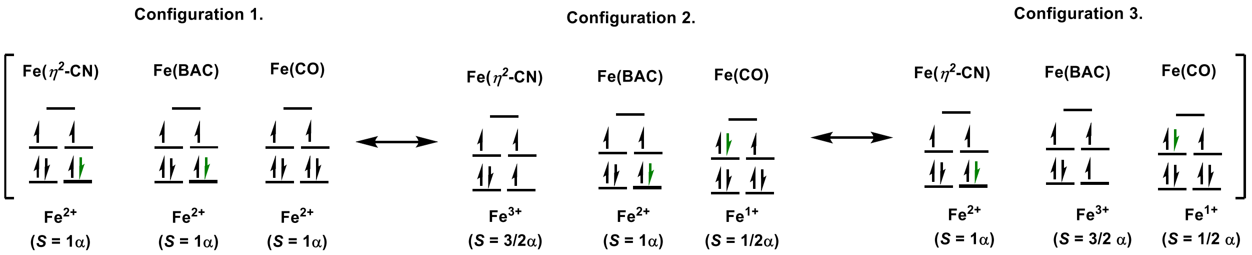

Figure S37. The three equivalent resonance structures associated with the ferromagnetic coupling between the three Fe-centers in **4**$^{-}$. The green arrow denotes the electrons that are delocalized between the resonance conformations. The oxidation and spin state are included.

D) References:

(1) Le, L. N. V.; Bailey, G. A.; Scott, A. G.; Agapie, T. Partial Synthetic Models of FeMoco with Sulfide and Carbyne Ligands: Effect of Interstitial Atom in Nitrogenase Active Site. *Proc. Natl. Acad. Sci. U.S.A.* **2021**, *118* (49), e2109241118. https://doi.org/10.1073/pnas.2109241118.

(2) Lis, Edward C.; Delafuente, D. A.; Lin, Y.; Mocella, C. J.; Todd, M. A.; Liu, W.; Sabat, M.; Myers, W. H.; Harman, W. D. The Uncommon Reactivity of Dihapto-Coordinated Nitrile, Ketone, and Alkene Ligands When Bound to a Powerful π-Base. *Organometallics* **2006**, *25* (21), 5051–5058. https://doi.org/10.1021/om060434o.

(3) Arikawa, Y.; Asayama, T.; Itatani, K.; Onishi, M. N−C Bond Formation of NO Ligands on Ruthenium Complexes with Concurrent Vinylic C−H Activation and Subsequent Proton-Induced Reactivities of the Resulting Nitrosovinyl Species. *J. Am. Chem. Soc.* **2008**, *130* (32), 10508–10509. https://doi.org/10.1021/ja804095u.

(4) Cowley, R. E.; Christian, G. J.; Brennessel, W. W.; Neese, F.; Holland, P. L. A Reduced (β-Diketiminato)Iron Complex with End-On and Side-On Nitriles: Strong Backbonding or Ligand Non-Innocence? *European Journal of Inorganic Chemistry* **2012**, *2012* (3), 479–483. https://doi.org/10.1002/ejic.201100787.

(5) Pyykkö, P.; Riedel, S.; Patzschke, M. Triple-Bond Covalent Radii. *Chemistry – A European Journal* **2005**, *11* (12), 3511–3520. https://doi.org/10.1002/chem.200401299.

(6) Chen, J.; Chen, T.; Norton, J. R.; Rauch, M. Insertion of Isonitriles into the Zr–CH3 Bond of Cp*2Zr(CH3)2 and Electrophilic Cleavage of the Remaining Methyl Group. *Organometallics* **2018**, *37* (23), 4424–4430. https://doi.org/10.1021/acs.organomet.8b00690.

(7) Valadez, T. N.; Norton, J. R.; Neary, M. C.; Quinlivan, P. J. Reaction of Cp*2Zr(2,3-Dimethylbutadiene) with Isonitriles and CO. *Organometallics* **2016**, *35* (18), 3163–3169. https://doi.org/10.1021/acs.organomet.6b00522.

(8) Klose, A.; Solari, E.; Ferguson, R.; Floriani, C.; Chiesi-Villa, A.; Rizzoli, C. Insertion Reactions of Isocyanides and Nitriles into Unsupported Iron-Aryl Bonds: The Synthesis of a Dimeric Iron(II) Homoleptic Iminoacyl Complex. *Organometallics* **1993**, *12* (7), 2414–2416. https://doi.org/10.1021/om00031a006.

(9) Klose, A.; Solari, E.; Floriani, C.; Chiesi-Villa, A.; Rizzoli, C.; Re, N. Magnetic Properties Diagnostic for the Existence of Iron(II)-Iron(II) Bonds in Dinuclear Complexes Which Derive from Stepwise Insertion Reactions on Unsupported Iron-Aryl Bonds. *J. Am. Chem. Soc.* **1994**, *116* (20), 9123–9135. https://doi.org/10.1021/ja00099a030.

(10) Boyarskiy, V. P.; Bokach, N. A.; Luzyanin, K. V.; Kukushkin, V. Yu. Metal-Mediated and Metal-Catalyzed Reactions of Isocyanides. *Chem. Rev.* **2015**, *115* (7), 2698–2779. https://doi.org/10.1021/cr500380d.

(11) Brown, A. C.; Thompson, N. B.; Suess, D. L. M. Evidence for Low-Valent Electronic Configurations in Iron–Sulfur Clusters. *J. Am. Chem. Soc.* **2022**, *144* (20), 9066–9073. https://doi.org/10.1021/jacs.2c01872.

(12) Brown, A. C.; Suess, D. L. M. Valence Localization in Alkyne and Alkene Adducts of Synthetic [Fe4S4]+ Clusters. *Inorg. Chem.* **2022**. https://doi.org/10.1021/acs.inorgchem.2c01353.

(13) Stoll, S.; Schweiger, A. EasySpin, a Comprehensive Software Package for Spectral Simulation and Analysis in EPR. *Journal of Magnetic Resonance* **2006**, *178* (1), 42–55. https://doi.org/10.1016/j.jmr.2005.08.013.

(14) Bruker. APEX3, 2012.
(15) Bruker. SADABS, 2001.
(16) Altomare, A.; Cascarano, G.; Giacovazzo, C.; Guagliardi, A.; Burla, M. C.; Polidori, G.; Camalli, M. SIR92 - a Program for Automatic Solution of Crystal Structures by Direct Methods. *Journal of Applied Crystallography* **1994**, *27* (3), 435.
(17) Palatinus, L.; Chapuis, G. SUPERFLIP - a Computer Program for the Solution of Crystal Structures by Charge Flipping in Arbitrary Dimensions. *Journal of Applied Crystallography* **2007**, *40* (4), 786–790.
(18) Betteridge, P. W.; Carruthers, J. R.; Cooper, R. I.; Prout, K.; Watkin, D. J. CRYSTALS Version 12: Software for Guided Crystal Structure Analysis. *Journal of Applied Crystallography* **2003**, *36* (6), 1487.
(19) Spek, A. PLATON SQUEEZE: A Tool for the Calculation of the Disordered Solvent Contribution to the Calculated Structure Factors. *Acta Crystallographica Section C* **2015**, *71* (1), 9–18.
(20) Furness, J. W.; Kaplan, A. D.; Ning, J.; Perdew, J. P.; Sun, J. Accurate and Numerically Efficient r2SCAN Meta-Generalized Gradient Approximation. *J. Phys. Chem. Lett.* **2020**, *11* (19), 8208–8215. https://doi.org/10.1021/acs.jpclett.0c02405.
(21) Benediktsson, B.; Bjornsson, R. Analysis of the Geometric and Electronic Structure of Spin-Coupled Iron–Sulfur Dimers with Broken-Symmetry DFT: Implications for FeMoco. *J. Chem. Theory Comput.* **2022**, *18* (3), 1437–1457. https://doi.org/10.1021/acs.jctc.1c00753.
(22) Caldeweyher, E.; Bannwarth, C.; Grimme, S. Extension of the D3 Dispersion Coefficient Model. *The Journal of Chemical Physics* **2017**, *147* (3), 034112. https://doi.org/10.1063/1.4993215.
(23) Ehlert, S.; Huniar, U.; Ning, J.; Furness, J. W.; Sun, J.; Kaplan, A. D.; Perdew, J. P.; Brandenburg, J. G. r2SCAN-D4: Dispersion Corrected Meta-Generalized Gradient Approximation for General Chemical Applications. *The Journal of Chemical Physics* **2021**, *154* (6), 061101. https://doi.org/10.1063/5.0041008.
(24) Weigend, F.; Ahlrichs, R. Balanced Basis Sets of Split Valence, Triple Zeta Valence and Quadruple Zeta Valence Quality for H to Rn: Design and Assessment of Accuracy. *Phys. Chem. Chem. Phys.* **2005**, *7* (18), 3297–3305. https://doi.org/10.1039/B508541A.
(25) Pantazis, D. A.; Chen, X.-Y.; Landis, C. R.; Neese, F. All-Electron Scalar Relativistic Basis Sets for Third-Row Transition Metal Atoms. *J. Chem. Theory Comput.* **2008**, *4* (6), 908–919. https://doi.org/10.1021/ct800047t.
(26) Rolfes, J. D.; Neese, F.; Pantazis, D. A. All-Electron Scalar Relativistic Basis Sets for the Elements Rb–Xe. *Journal of Computational Chemistry* **2020**, *41* (20), 1842–1849. https://doi.org/10.1002/jcc.26355.
(27) Barone, V.; Cossi, M. Quantum Calculation of Molecular Energies and Energy Gradients in Solution by a Conductor Solvent Model. *J. Phys. Chem. A* **1998**, *102* (11), 1995–2001. https://doi.org/10.1021/jp9716997.
(28) Pipek, J.; Mezey, P. G. A Fast Intrinsic Localization Procedure Applicable for Ab Initio and Semiempirical Linear Combination of Atomic Orbital Wave Functions. *The Journal of Chemical Physics* **1989**, *90* (9), 4916–4926. https://doi.org/10.1063/1.456588.
(29) Römelt, M.; Ye, S.; Neese, F. Calibration of Modern Density Functional Theory Methods for the Prediction of 57Fe Mössbauer Isomer Shifts: Meta-GGA and Double-Hybrid Functionals. *Inorg. Chem.* **2009**, *48* (3), 784–785. https://doi.org/10.1021/ic801535v.

(30) Neese, F. Prediction and Interpretation of the 57Fe Isomer Shift in Mössbauer Spectra by Density Functional Theory. *Inorganica Chimica Acta* **2002**, *337*, 181–192. https://doi.org/10.1016/S0020-1693(02)01031-9.
(31) Greatrex, R.; Greenwood, N. N. Mössbauer Spectra, Structure, and Bonding in Iron Carbonyl Derivatives. *Discuss. Faraday Soc.* **1969**, *47* (0), 126–135. https://doi.org/10.1039/DF9694700126.
(32) Sinnecker, S.; Slep, L. D.; Bill, E.; Neese, F. Performance of Nonrelativistic and Quasi-Relativistic Hybrid DFT for the Prediction of Electric and Magnetic Hyperfine Parameters in 57Fe Mössbauer Spectra. *Inorg. Chem.* **2005**, *44* (7), 2245–2254. https://doi.org/10.1021/ic048609e.
(33) Neese, F.; Wennmohs, F.; Becker, U.; Riplinger, C. The ORCA Quantum Chemistry Program Package. *The Journal of Chemical Physics* **2020**, *152* (22), 224108. https://doi.org/10.1063/5.0004608.
